# Superanionic DNA: enzymatic synthesis of hypermodified DNA bearing four different anionic substituents at all four nucleobases

**DOI:** 10.1093/nar/gkad893

**Published:** 2023-10-23

**Authors:** Natalia Kuprikova, Marek Ondruš, Lucie Bednárová, Miguel Riopedre-Fernandez, Lenka Poštová Slavětínská, Veronika Sýkorová, Michal Hocek

**Affiliations:** Institute of Organic Chemistry and Biochemistry, Czech Academy of Sciences, Flemingovo nam. 2, CZ-16000 Prague 6, Czech Republic; Department of Organic Chemistry, Faculty of Science, Charles University, Hlavova 8, CZ-12843 Prague 2, Czech Republic; Institute of Organic Chemistry and Biochemistry, Czech Academy of Sciences, Flemingovo nam. 2, CZ-16000 Prague 6, Czech Republic; Institute of Organic Chemistry and Biochemistry, Czech Academy of Sciences, Flemingovo nam. 2, CZ-16000 Prague 6, Czech Republic; Institute of Organic Chemistry and Biochemistry, Czech Academy of Sciences, Flemingovo nam. 2, CZ-16000 Prague 6, Czech Republic; Institute of Organic Chemistry and Biochemistry, Czech Academy of Sciences, Flemingovo nam. 2, CZ-16000 Prague 6, Czech Republic; Institute of Organic Chemistry and Biochemistry, Czech Academy of Sciences, Flemingovo nam. 2, CZ-16000 Prague 6, Czech Republic; Institute of Organic Chemistry and Biochemistry, Czech Academy of Sciences, Flemingovo nam. 2, CZ-16000 Prague 6, Czech Republic; Department of Organic Chemistry, Faculty of Science, Charles University, Hlavova 8, CZ-12843 Prague 2, Czech Republic

## Abstract

We designed and synthesized a set of four 2′-deoxyribonucleoside 5′-*O*-triphosphates (dNTPs) derived from 5-substituted pyrimidines and 7-substituted 7-deazapurines bearing anionic substituents (carboxylate, sulfonate, phosphonate, and phosphate). The anion-linked dNTPs were used for enzymatic synthesis of modified and hypermodified DNA using KOD XL DNA polymerase containing one, two, three, or four modified nucleotides. The polymerase was able to synthesize even long sequences of >100 modified nucleotides in a row by primer extension (PEX). We also successfully combined two anionic and two hydrophobic dNTPs bearing phenyl and indole moieties. In PCR, the combinations of one or two modified dNTPs gave exponential amplification, while most of the combinations of three or four modified dNTPs gave only linear amplification in asymmetric PCR. The hypermodified ONs were successfully re-PCRed and sequenced by Sanger sequencing. Biophysical studies including hybridization, denaturation, CD spectroscopy and molecular modelling and dynamics suggest that the presence of anionic modifications in one strand decreases the stability of duplexes while still preserving the B-DNA conformation, whilst the DNA hypermodified in both strands adopts a different secondary structure.

## Graphical Abstract

**Graphical Abstract:**
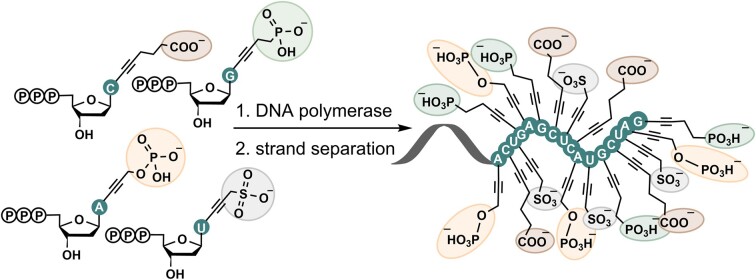

## Introduction

Base-modified DNA (1) find a plethora of applications in DNA enzymes (2–4), aptamers (5–8) and other functional (macro)molecules and supramolecular assemblies (9–11). They can be prepared either chemically using modified nucleoside phosphoramidites or enzymatically using DNA polymerase and modified 2′-deoxyribonucleoside 5′-*O*-triphosphates (dNTPs) (12,13). The latter approach can be also applied for *in vitro* selection experiments (i.e. SELEX). In most of these applications, the DNA contained one (14–16) or two modified nucleotides (17) that bring an additional function, while the other nucleotides were non-modified. There have been only several scattered reports (18–21) on fully-modified DNA where each nucleotide is bearing a different modification (further referred to as hypermodified DNA). Recently, we have reported (22) the enzymatic synthesis of hypermodified DNA bearing four different hydrophobic substituents as analogues of hydrophobic amino acid sidechains either through primer extension (PEX) or asymmetric PCR (aPCR). Later on, we developed (23) the synthesis of hypermodified hydrophobic single-stranded oligonucleotides (ssONs) using reverse transcription from RNA templates and ribonucleotide-containing primers.

Anionic (or dissociated acidic) substituents attached to anionic DNA lead to destabilization of duplexes due to increased repulsion of the hybridized strands (24). Nevertheless, several anionic groups are known and have been successfully incorporated into DNA and studied for some applications. 5-Carboxycytosine is a naturally occurring DNA base that is an intermediate in the active demethylation pathway (25). Several non-natural carboxylate- or phosphate-linked nucleotides were used in enzymatic synthesis of functionalized DNA (18,20,26) and studies of mechanical properties (24) or selection of DNAzymes (27), whereas dicarboxylate-linked 2′-deoxyuridine and -cytidine dNTPs were used for construction of polyanionic DNA for potential applications in Si-nanowire biosensors (28). Amino-acid-linked nucleotides (29) were used to form protein-like constructs, while boronic-acid-linked nucleotides were introduced (30,31) for interactions with carbohydrates. To the best of our knowledge, no example of hypermodified DNA containing multiple anionic nucleotides has been reported. Therefore, we report here on the design and synthesis of a complete set of all four nucleotides each containing a different anionic substituent and their use for polymerase synthesis of ‘superanionic’ hypermodified DNA.

## Materials and methods

### Synthesis of modified dN^R^TPs bearing acidic groups

**Scheme 1.**
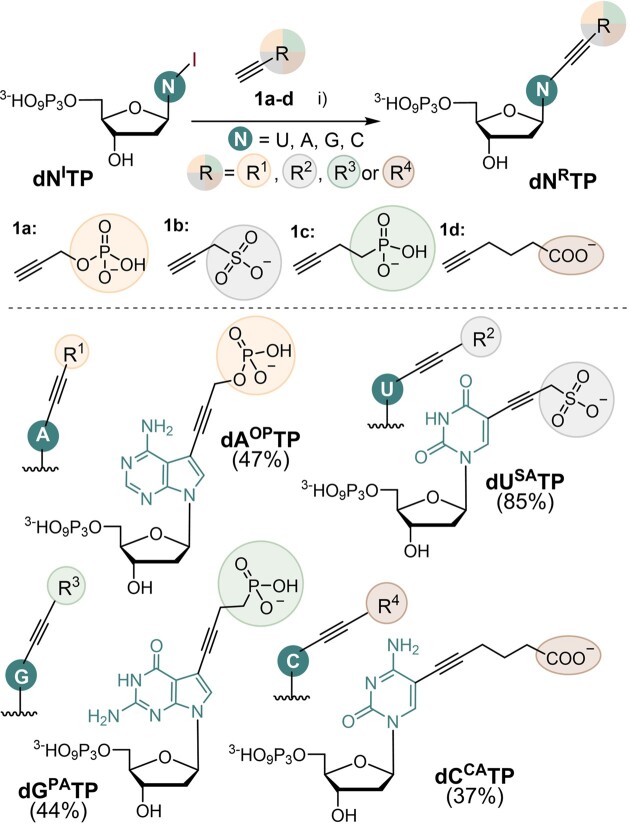
Design and synthesis of modified **dN^R^TP**s (R = SA, OP, PA, CA; N = U, A, G, C). Reagents and conditions: (i) R-C≡CH (1.5 equiv.), [Pd(PPh_3_)_2_Cl_2_] (5 mol%), CuI (10 mol%), PPh_3_ (2 mol%), Et_3_N (10 equiv.), MeCN/H_2_O 1:2, 60°C, 1 h, under Ar, 37–85%.

A 1:2 mixture of MeCN/H_2_O (0.5 mL) followed by Et_3_N (10 equiv.) were added through a septum to an argon-purged flask charged with a halogenated nucleoside triphosphate **dN^I^TP** (N = U, A, G, C) (32–35) (1 equiv.), a corresponding alkyne **1a–d** (Scheme 1; Supplementary Scheme S1 in SI) (1.5 equiv.), CuI (10 mol%), PPh_3_ (2 mol%) and [Pd(PPh_3_)_2_Cl_2_] (5 mol%). The mixture was stirred for 1 h at 60°C under argon atmosphere. Solvents were evaporated under vacuum. The product was purified by HPLC with linear gradient of 0.1 M TEAB (triethylammonium bicarbonate) in H_2_O to 0.1 M TEAB in H_2_O/MeOH (1:1) as eluent followed by lyophilization to obtain a solid product (more details in SI, section 1.1; information about the source of used substances can be found in General remarks in section 1 in Supporting Information).

### Multiple incorporation of four modified dN^R^TPs by PEX

**Figure 1.**
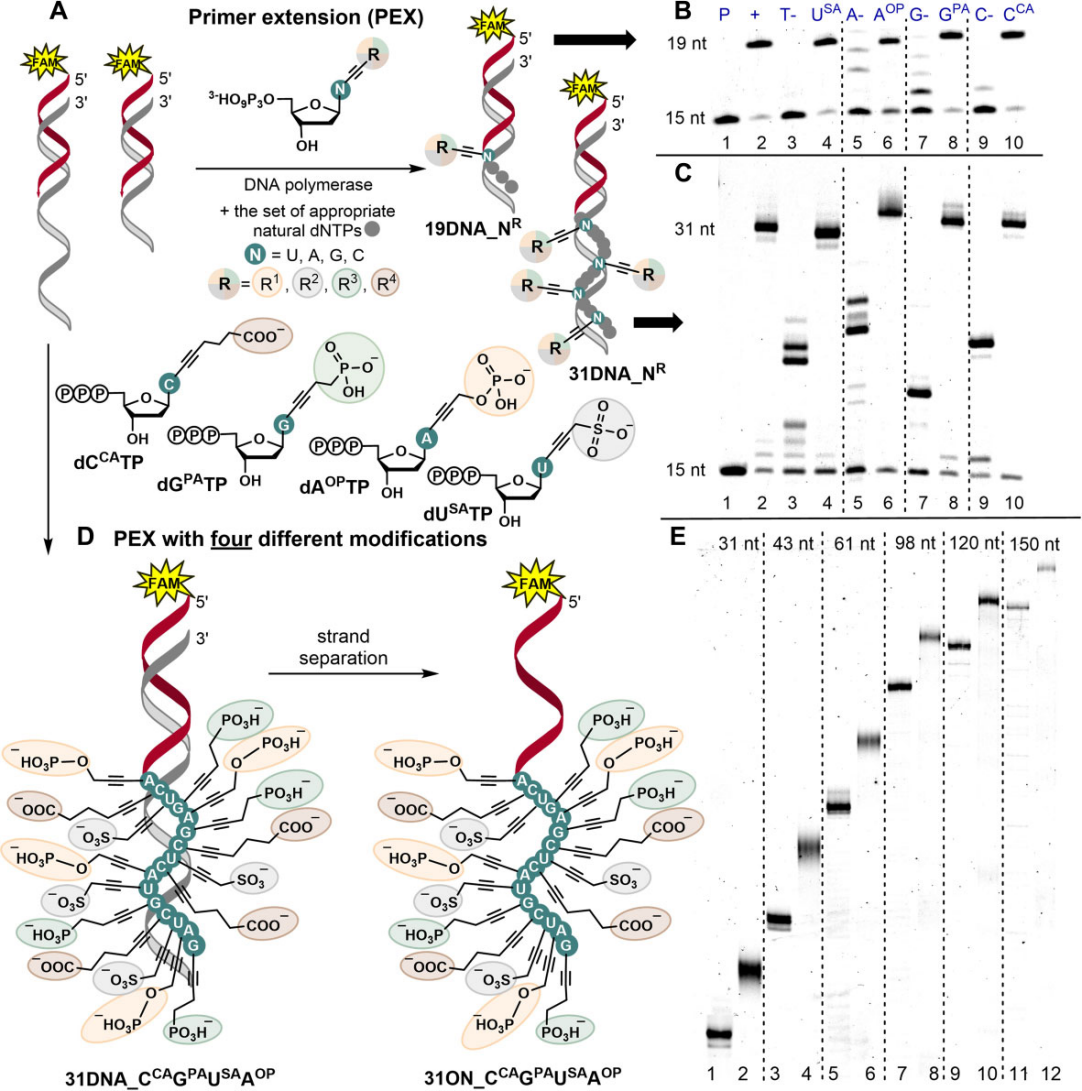
(**A**) PEX reaction using one modified **dN^R^TP** (R = SA, OP, PA, CA; N = U, A, G, C). (**B**) Denaturing PAGE analysis of PEX using Vent(exo^–^) DNA polymerase, 5′-(6-FAM)-labelled primer, and 19-mer appropriate template; (**C**) 31-mer template [lanes: (1) primer; (2) positive control; (4), (6), (8), (10) reactions containing either **dU^SA^TP**, **dA^OP^TP**, **dG^PA^TP** or **dC^CA^TP**; (3), (5), (7), (9) negative controls, in absence of either dUTP, dATP, dGTP or dCTP]. **(D**) PEX reaction using all four modified **dN^R^TP**s followed by strand separation. (**E)** Denaturing PAGE analysis of PEX using Vent(exo^–^) DNA polymerase, 5′-(6-FAM)-labelled primer, set of four modified **dN^R^TP**s, and 31-mer (lane 2), 43-mer (lane 4), 61-mer (lane 6), 98-mer (lane 8), 120-mer (lane 10), and 150-mer (lane 12) templates; lanes 1, 3, 5, 7, 9 and 11: positive controls with natural dNTPs.

The reaction mixture (10 μl) contained 31-mer template Temp^Prb4basII^ (3 μM, 0.75 μl), primer Prim^248short^-FAM (3 μM, 0.5 μl) (for sequences see Supplementary Table S1 in SI), **dC^CA^TP** (0.25 mM, 1 μl), **dG^PA^TP** (2 mM, 1 μl), **dU^SA^TP** (2 mM, 1 μl), **dA^OP^TP** (2 mM, 1 μl) (see structures in Scheme 1), Vent(exo^–^) DNA polymerase (1 U), and the enzyme reaction buffer (10×, 1 μl). The positive control contained 0.5 U of Vent(exo^–^) DNA polymerase and natural dNTPs (1 mM, 1 μl). The reaction mixture was incubated for 30 min at 60°C, stopped by addition of PAGE stop solution (10 μl) and denatured for 3 min at 95°C. Samples were analyzed by PAGE and visualized using fluorescence imaging (Figure 1E; section 2.3 in SI).

### Multiple incorporation of four modified dN^R^TPs by aPCR

The reaction mixture (10 μl) contained 98-mer template Temp^FVL-A^ (5 μM, 0.5 μl), primer Prim^LT25TH^-FAM (10 μM, 1 μl) (for sequences, see Supplementary Table S1 in SI), set of four modified **dN^R^TP**s (1 μl, conditions specified in Supplementary Table S11 in SI), KOD XL DNA polymerase (2.5 U) and the enzyme reaction buffer (10×, 1 μl). The positive control contained 0.5 U of KOD XL DNA polymerase and all four natural dNTPs (2 mM, 2 μl). All reaction mixtures were under cycling protocol: 94°C for 3 min, followed by 30 cycles at 94°C for 1 min, 53°C for 1 min, and 72°C for 6 min, followed by a final elongation step at 72°C for 5 min. Samples were analyzed by PAGE and visualized using fluorescence imaging (Figure 3D; Supplementary Figure S10 in SI).

### Re-PCR of a fully modified template to natural DNA

The reaction mixtures (20 μl) contained a modified template (either **118ON_C^CA^G^PA^U^SA^A^OP^**, **118ON_C^CA^G^PA^U^EPh^A^EIn^** or **118cON_C^CA^G^PA^U^SA^A^OP^**) (0.5 μM, 1 μl), forward primer Prim^Flank^-FAM and a reverse primer (Prim^L20^ in the case of **118ON_C^CA^G^PA^U^SA^A^OP^** and **118ON_C^CA^G^PA^U^EPh^A^EIn^**, Prim^LT25TH^ in the case of **118cON_C^CA^G^PA^U^SA^A^OP^**) (10 μM, 2 μl each) (for sequences see Supplementary Tables S1 and S2 in SI), all four natural dNTPs (1 mM, 4.5 μl), KOD XL DNA polymerase (1.25 U), and the enzyme reaction buffer (10X, 2 μl). All reaction mixtures were under cycling protocol: 94°C for 3 min, followed by 15 cycles at 94°C for 30 s, 50°C for 1 min, and 72°C for 1 min, followed by a final elongation step at 72°C for 5 min. The obtained DNA duplexes were purified using Agencourt AMPure XP magnetic particles, analyzed by PAGE and visualized using fluorescence imaging (Supplementary Figure S11 in SI).

### Preparation of 98DNA_dsC^CA^G^PA^U^SA^A^OP^ containing both strands fully modified with anionic modifications

Oligonucleotides (ONs) **98ON_C^CA^G^PA^U^SA^A^OP^** and **98cON_C^CA^G^PA^U^SA^A^OP^** (preparation described below) were annealed together in Tris-HCl buffer (10 mM, 1 mM EDTA, 100 mM NaCl, pH 7.5–8.0) under following protocol: 95°C for 5 min, followed by gradual cooling to 25°C for 90 min. The annealed product was analyzed by agarose gel containing GelRed intercalator and visualized using fluorescence imaging (Supplementary Figure S12 in SI).

Preparation of **98ON_C^CA^G^PA^U^SA^A^OP^** and **98cON_C^CA^G^PA^U^SA^A^OP^**:

1. Synthesis of double-stranded PEX products **98DNA_C^CA^G^PA^U^SA^A^OP^** and **98cDNA_C^CA^G^PA^U^SA^A^OP^**: The reaction mixture (10 μl) containing a template (100 μM, 0.5 μl) and a primer (100 μM, 0.5 μl) (Temp^FVL-A^ and Prim^LT25TH^ in the case of **98DNA_C^CA^G^PA^U^SA^A^OP^**, Temp^FVL-A_comp^ and Prim^L20^ in the case of **98cDNA_C^CA^G^PA^U^SA^A^OP^**) (for sequences see Supplementary Tables S1 and S2 in SI), set of four modified **dN^R^TP**s (R = SA, OP, PA, CA; N = U, A, G, C) (4 mM, 1.25 μl each), KOD XL DNA polymerase (1.25 U), and the enzyme reaction buffer (10×, 1 μl) was incubated for 40 min at 60°C and stopped by cooling to 8°C. The reactions were repeated 63 times to obtain sufficient DNA concentration for measurements.
2. The modified strands **98ON_C^CA^G^PA^U^SA^A^OP^** and **98cON_C^CA^G^PA^U^SA^A^OP^** (for sequences see Supplementary Table S2 in SI) were separated from the templates using gel extraction method: **98DNA_C^CA^G^PA^U^SA^A^OP^** and **98cDNA_C^CA^G^PA^U^SA^A^OP^** were mixed with PAGE stop solution and loaded on 12.5% preparative denaturing polyacrylamide gel (1.5 mm thick) under denaturing conditions (1 h, 50°C, 1× TBE buffer). After the run, modified ONs and templates were visualized by UV lamp, and the areas containing **98ON_C^CA^G^PA^U^SA^A^OP^** and **98cON_C^CA^G^PA^U^SA^A^OP^** were cut out. In order to extract the products, Pur-A-Lyze Maxi Dialysis Kit was used. The cut-out pieces of gel were placed into Pur-A-Lyze Maxi Dialysis Kits containing 3 ml of 1× TAE buffer. Further, the dialysis kit was placed into a horizontal electrophoretic system in a way that its membrane was in a perpendicular position to the current direction. Then, current was applied (45 min, 100 V) and the product was eluted from the piece of gel and trapped on the dialysis column membrane. To detach ONs from the dialysis column membrane, the current was applied in the reverse direction (1 min, 100 V). The obtained **98ON_C^CA^G^PA^U^SA^A^OP^** and **98cON_C^CA^G^PA^U^SA^A^OP^** were desalted and concentrated using Amicon Ultra-0.5 Centrifugal Filters.

## Results and discussion

We designed nucleotides bearing four different acidic functions that would be partially or fully deprotonated under neutral conditions forming the corresponding monoanions, namely carboxylate, sulfonate, phosphonate, and phosphate. The acidic groups were attached to position 5 of pyrimidines or 7 of 7-deazapurines through alkyne linker to facilitate their substrate activity with DNA polymerases (36,37). The synthesis of the target modified nucleoside triphosphates **dU^SA^TP**, **dC^CA^TP**, **dA^OP^TP**, **dG^PA^TP** was performed through direct aqueous-phase Sonogashira cross-coupling (32) of 5-iodopyrimidine- or 7-iodo-7-deazapurine 2′-deoxyribonucleoside triphosphates (**dN^I^TP**s) (32–35) with the corresponding functionalized alkynes **1a–d** (Scheme 1). The reactions were carried out under argon in presence of [Pd(PPh_3_)_2_Cl_2_] catalyst, CuI, Et_3_N and PPh_3_ in acetonitrile/water for 1 h at 60°C. The desired modified triphosphates were purified by HPLC and isolated in moderate to good yields (37–85%) (for details see section 1.1 in the Supporting Information; for copies of NMR spectra see section 7 in SI). This approach was shorter and gave higher total yields compared to previously reported (20,26) synthetic pathways used to obtain modified dNTPs bearing carboxylic groups.

The modified nucleotides **dU^SA^TP**, **dC^CA^TP**, **dA^OP^TP**, **dG^PA^TP** were tested as substrates for two DNA polymerases—Vent(exo–) and KOD XL. The primer extension (PEX) experiments were performed with either 19-mer or 31-mer templates (encoding for one or four modified nucleotides) and 15-mer primer using one modified nucleotide in presence of the other three natural dNTPs (Figure 1A, for lists of all oligonucleotides see Supplementary Tables S1 and S2 in the Supporting Information; for detailed procedure see sections 2.1 and 2.2 and Supplementary Tables S3, S4 in SI). KOD XL DNA polymerase (a mixture of KOD polymerase from *Thermococcus kodakaraensis* and its exonuclease-deficient mutant) (38) was selected since it has previously been reported to efficiently incorporate high density of modifications (21,22). Vent(exo^–^) DNA polymerase was chosen as an alternative, more affordable DNA polymerase without exonuclease activity. Figure 1B and C show PAGE analysis of the PEX products obtained with Vent(exo^–^) DNA polymerase (also Supplementary Figures S1B and S2B in SI). Similar results were observed with KOD XL DNA polymerase, see Supplementary Figures S1A and S2A in SI. In all cases, full-length PEX products containing either 1 or 4 modified nucleotides were formed. The identity of the products was confirmed by MALDI-TOF analysis of oligonucleotides (ONs) obtained by larger scale PEX with dual-biotinylated templates followed by magnetoseparation purification (for samples preparation procedures see section 2.6; for MALDI-TOF results see Supplementary Table S6 and Supplementary Figures S22–S29 in SI).

The next step of our work was to try different combinations of **dU^SA^TP**, **dC^CA^TP**, **dA^OP^TP**, **dG^PA^TP** in PEX. We proceeded with Vent(exo^–^) DNA polymerase since both polymerases had shown similar results in PEX with one modified **dN^R^TP**. We tried different combinations of two or three modified **dN^R^TP**s (always complemented with the remaining non-modified dNTPs) and in all cases we observed formation of full-length products (see section 2.3, Supplementary Table S5, and Supplementary Figure S3 in SI). Then, we performed PEX with the combination of all four modified **dN^R^TP**s using templates of different lengths (31, 43, 61, 98, 120, 150-mer) (for details see section 2.4 and Supplementary Figure S4 in SI). The PAGE analysis (Figure 1E) confirmed the formation of full-length hypermodified superanionic PEX products containing 16–125 modified nucleotides in a row. The electrophoretic mobility of the resulting modified PEX products significantly differed from the mobility of the same size non-modified DNAs apparently due to the increased anionic character and higher mass of the modified ONs. The smears, which were observed in some of the PEX product bands, may arise from secondary structures with different stability or higher aggregates that cannot be resolved under the conditions applied in standard denaturing PAGE electrophoretic analysis.

**Figure 2.**
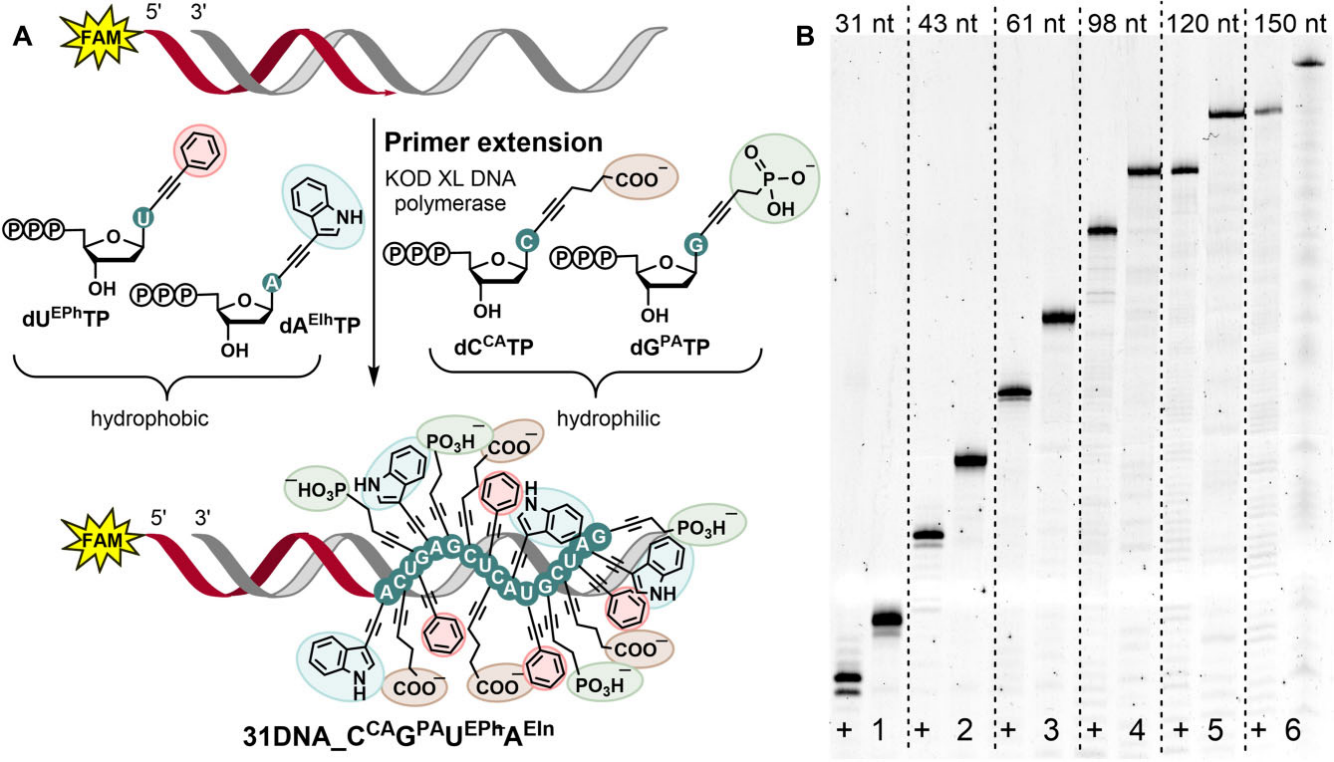
(**A**) PEX reaction with a set of four modified **dN^R^TP**s – **dC^CA^TP**, **dG^PA^TP**, **dA^EIn^TP**, and **dU^EPh^TP**. (**B**) Denaturing PAGE analysis of the PEX with KOD XL DNA polymerase, 5′-(6-FAM)-labelled primer, and 31-mer (lane 1), 43-mer (lane 2), 61-mer (lane 3), 98-mer (lane 4), 120-mer (lane 5) and 150-mer (lane 6) templates. (+): positive controls with natural dNTPs.

For prospective applications in selection of functional nucleic acids (NA), we wanted to test whether the anionically modified **dN^R^TP**s can be combined with hydrophobic nucleotides in PEX. Thus, we took previously reported (22) **dU^EPh^TP** and **dA^EIn^TP** and performed PEX reactions in combination with **dC^CA^TP** and **dG^PA^TP** (Figure 2A). The reactions were carried out using templates of different lengths (31, 43, 61, 98, 120, 150-mer) and KOD XL DNA or Vent(exo^–^) DNA polymerase (for details see section 2.5 and Supplementary Figure S5 in SI). The formation of full-length hypermodified PEX products was observed with KOD XL DNA polymerase (Figure 2B) but not with Vent(exo^–^). Similarly to the above-mentioned superanionic DNA, the different electrophoretic mobility of the modified PEX products in comparison to the natural ONs was observed, but the bands were not smeared. When using 5′-dual-biotinylated template in PEX followed by a magnetoseparation with streptavidin-coated magnetic beads, the hypermodified 31-mer ONs bearing either all four anionic or a combination of two anionic and two hydrophobic modifications were separated from the templates and characterized by MALDI-TOF analysis (Supplementary Table S6 and Supplementary Figures S30 and S31 in SI).

**Figure 3.**
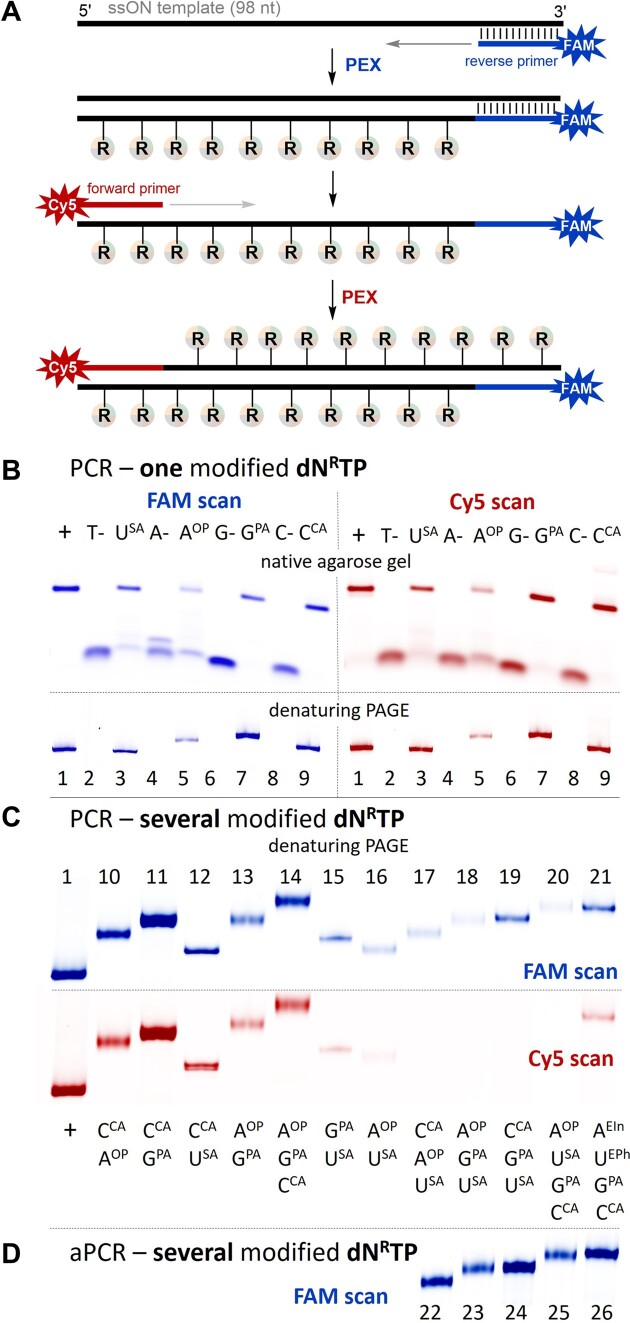
(**A**) Scheme of reverse and forward primers extension in PCR. (B–D) Analysis of PCR experiments with **dN^R^TP**s using 5′-(6-FAM)-labelled reverse primer, 5′-Cy5-labelled forward primer, 98-mer template, and KOD XL DNA polymerase. (**B**) Native agarose gel and denaturing PAGE of PCR products using one modified **dN^R^TP**: lanes (1) positive control; (3), (5), (7), (9) reactions using **dU^SA^TP**, **dA^OP^TP**, **dG^PA^TP** or **dC^CA^TP** in combination with the other three native dNTPs; (2), (4), (6), (8) negative controls, in absence of dTTP, dATP, dGTP or dCTP. (**C**) Denaturing PAGE of PCR products using combinations of modified **dN^R^TP**: lanes 10-21 (lane 11 – we used Vent(exo^–^) polymerase for **dC^CA^TP** and **dG^PA^TP** combination). (**D**) Denaturing PAGE of aPCR products using combinations of modified **dN^R^TP**: lanes 22-26.

After we confirmed that the modified anionic **dN^R^TP**s are suitable substrates for DNA polymerases in PEX, we studied their use in polymerase chain reaction (PCR). For successful exponential amplification not only the modified **dN^R^TP**s have to be good substrates for a DNA polymerase, but also the polymerase needs to be able to read through the modified template to synthetize another modified strand. This is challenging because the anionic modifications may strongly interact with a DNA polymerase. To investigate whether the modified nucleotides are proficient substrates in PCR, the reverse primer was labelled with 6-FAM, while the forward primer was labelled with Cy5 (Figure 3A). At first, each of the **dN^R^TP**s has been studied in PCR reactions using Vent(exo^–^) and KOD XL DNA polymerases in presence of the other three natural dNTPs and a single-stranded 98-mer template (for details see section 2.8 and Supplementary Table S7 in SI). After 30 cycles, PCR products were analyzed by native agarose gel and denaturing PAGE using either FAM or Cy5 scan (for uncropped gels see Supplementary Figure S6 in SI). As a result, all modified **dN^R^TP**s worked well with KOD XL DNA polymerase giving full-length amplicon products (Figure 3B). However, incorporation of **dU^SA^TP** and **dA^OP^TP** using Vent(exo^–^) DNA polymerase proceeded poorly (see Supplementary Figure S6B). Considering this, we used only KOD XL DNA polymerase for further PCR experiments involving **dU^SA^TP** and **dA^OP^TP**.

A PCR experiment with combinations of several modified **dN^R^TP**s is more challenging. We performed a systematic study of PCR reactions with different combinations of two, three, and all four **dN^R^TP**s using a single-stranded 98-mer template and KOD XL DNA polymerase (or Vent(exo^–^) polymerase; for details and unmodified gels see sections 2.9, 2.10, 2.11, Supplementary Tables S8–S10, and Supplementary Figures S7–S9 in SI). Figure 3C shows visualization of the products on denaturing PAGE. The 5′-(6-FAM)-labelled products of reverse primer extension have been obtained for all combinations, however, in a number of cases extension of 5′-(Cy5)-labelled forward primer did not proceed at all. Analyzing the gels, one can conclude that the worst results were observed in most experiments with **dU^SA^TP**. This can be related to inhibition of a DNA polymerase by the sulfonic group (39) attached to the modified triphosphate. Nevertheless, we succeeded to improve the yields of reverse primer extension for combinations of three and four **dN^R^TP**s through carefully optimized PCR conditions with the increased amount of template and in the absence of forward primer (for details, see section 2.12, Supplementary Table S11, and Supplementary Figure S10 in SI). The results of successful asymmetric PCR (aPCR) with linear amplification are shown in Figure 3D.

**Figure 4.**
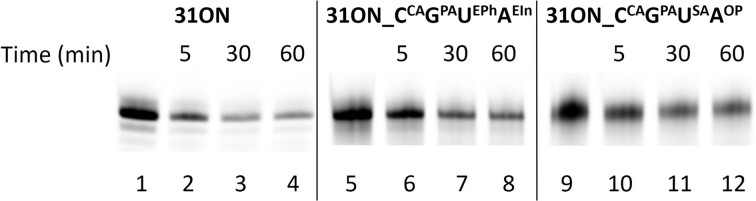
PAGE analysis of stability of natural **31ON** (lanes 2–4), mixed **31ON_C^CA^G^PA^U^EPh^A^EIn^** (lanes 6–8) and anionic **31ON_C^CA^G^PA^U^SA^A^OP^** (lanes 10–12) in the presence of 0.01U of DNase I for 5 min (lanes 2, 6, 10), 30 min (lanes 3, 7, 11), and 60 min (lanes 4, 8, 12). Lanes 1, 5 and 9 were not incubated with DNase I.

To investigate the influence of the high density of negatively charged groups on nuclease degradation of DNA, we have prepared four 5′-(6-FAM)-labelled 31-mer ONs: natural **31ON**, fully-modified **31ON_C^CA^G^PA^U^SA^A^OP^** bearing only anionic modifications, **31ON_C^CA^G^PA^U^EPh^A^EIn^** with both hydrophobic and anionic modifications (synthesis is described in sections 2.6 and 5 in SI; for sequences see Supplementary Table S2 in SI), as well as previously reported (22) hydrophobic-only hypermodified **31ON_C^EAlk^G^EiPr^U^EPh^A^EIn^**. The obtained ONs were incubated with DNase I for 5, 30, and 60 minutes at 37°C (for details see Section 5, Supplementary Table S14, and Supplementary Figure S21 in SI). The experiments were performed in triplicate and the reactions were analyzed by PAGE and visualized using fluorescence imaging (Figure 4) and the average values of intact DNA recovery are given in Table 1. The results clearly show that the anionic modifications enhance ON resistance towards DNase I digestion. The superanionic **31ON_C^CA^G^PA^U^SA^A^OP^** was found ca. twice more resistant compared to the natural **31ON**. The mixed anionic-hydrophobic **31ON_C^CA^G^PA^U^EPh^A^EIn^** was still significantly more stable towards degradation, whereas the hydrophobic-only **31ON_C^EAlk^G^EiPr^U^EPh^A^EIn^** showed similar low stability as the natural ON (for details, see Section 5, Supplementary Table S14, and Supplementary Figure S21 in SI).

**Table 1.** Recovery (expressed in percent) of natural and hypermodified DNA after incubation with 0.01U of DNase I

	Incubation time with DNase I (0.01U)		
DNA title	5 min	30 min	60 min
**31ON**	35 ± 3%	21 ± 1%	18 ± 1%
**31ON_C^EAlk^G^EiPr^U^EPh^A^EIn^**	38 ± 5%	16 ± 0%	12 ± 1%
**31ON_C^CA^G^PA^U^EPh^A^EIn^**	61 ± 2%	37 ± 2%	29 ± 5%
**31ON_C^CA^G^PA^U^SA^A^OP^**	64 ± 6%	50 ± 3%	50 ± 2%

**Scheme 2.**
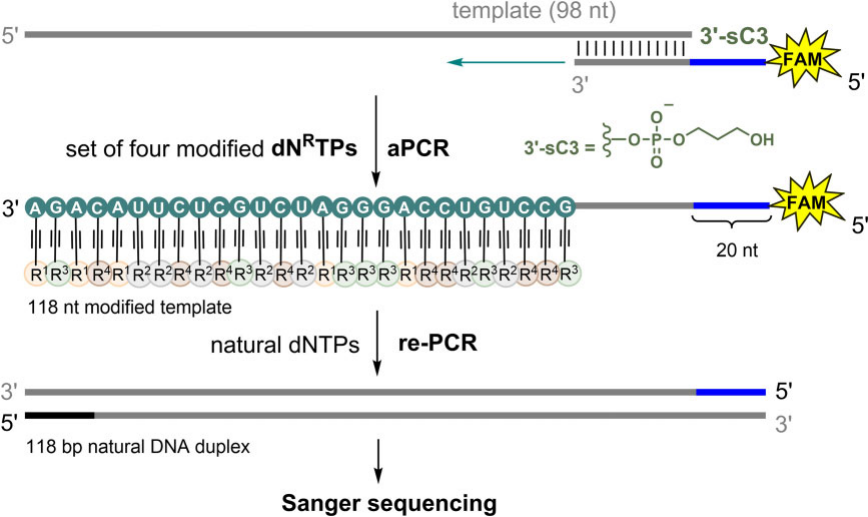
Asymmetric PCR synthesis with an extended primer and a set of four modified **dN^R^TP**s followed by re-PCR with natural dNTPs to prepare dsDNA for Sanger sequencing.

The hypermodified ONs need to be sequencable for any applications in selection of functional NA. Previously, we developed a method for sequencing hypermodified ONs bearing hydrophobic groups that ensures sequencing of the modified strand rather than of the unmodified template (Scheme 2; also Supplementary Schemes S2, S3 in SI) (22). The template was modified at 3′-end with three carbon spacer (sC3) preventing any undesirable extension during aPCR. The 5′-end of the FAM-labelled primer was extended with a 20-nt flanking sequence, which served as a new primer region for re-PCR (for sequences see Supplementary Table S1). Then the aPCR reaction with a 98-mer template, the extended primer, KOD XL DNA polymerase, and a set of four modified **dN^R^TP**s – either **dC^CA^TP**, **dG^PA^TP**, **dA^OP^TP**, and **dU^SA^TP** or **dC^CA^TP**, **dG^PA^TP**, **dA^EIn^TP** and **dU^EPh^TP** – was performed. To remove the remaining primers and **dN^R^TP**s, aPCR product was purified by Agencourt AMPure XP magnetic particles, and then the obtained fully-modified 118-mer ON was used as a template for re-PCR reaction in presence of KOD XL DNA polymerase (see section 2.13 and Supplementary Figure S11 in SI). The re-PCR product was then subjected to Sanger sequencing confirming the fidelity of the replication of a hypermodified ON and the possibility to correctly sequence the hypermodified superanionic as well as mixed anionic-hydrophobic DNA (for samples preparation see section 2.14 and Supplementary Table S12 in SI; for results of Sanger sequencing see section 2.15 in SI).

To study the influence of the anionic modifications on the properties and stability of DNA, temperature-dependent measurements were performed with DNA hypermodified in one or in both strands. 98bp DNA duplexes named **98DNA_C^CA^G^PA^U^SA^A^OP^** (**dC^CA^TP**, **dG^PA^TP**, **dA^OP^TP**, **dU^SA^TP** used as building blocks) and **98DNA_C^CA^G^PA^U^EPh^A^EIn^** (**dC^CA^TP**, **dG^PA^TP**, **dA^EIn^TP**, **dU^EPh^TP** used as building blocks) containing 73 modified bases in one strand were prepared using semipreparative PEX reactions and dissolved in a buffer (10 mM Tris, 65 mM NaCl, 1 mM EDTA, pH 7.5–8.0). In order to prepare 98bp DNA duplex **98DNA_dsC^CA^G^PA^U^SA^A^OP^** containing both strands fully-modified with anionic modifications, two complementary modified ssONs **98ON_C^CA^G^PA^U^SA^A^OP^** and **98cON_C^CA^G^PA^U^SA^A^OP^** were obtained through PEX followed by separation of the modified strands from templates using PAGE (as described in Materials and Methods). Further, **98ON_C^CA^G^PA^U^SA^A^OP^** and **98cON_C^CA^G^PA^U^SA^A^OP^** were dissolved in a buffer (10 mM Tris, 100 mM NaCl, 1 mM EDTA, pH 7.5–8.0) and annealed with each other giving **98DNA_dsC^CA^G^PA^U^SA^A^OP^**. An unmodified 98bp **98DNA** was obtained by PCR as a reference (for all procedures see section 3.1 in SI; for sequences see Supplementary Table S2 in SI).

Previously, a significant duplex stabilization has been reported for DNA containing 5-propynylpyrimidines (40) or all four alkyne-linked nucleobases (22) due to the increased polarizability and π–π-stacking effect of the modified nucleobases compared to natural counterpart. However, in both cases of anionic **98DNA_C^CA^G^PA^U^SA^A^OP^** and anionic-hydrophobic **98DNA_C^CA^G^PA^U^EPh^A^EIn^** the melting temperatures (*T*_m_) decreased from 78.6°C (obtained for natural **98DNA**) to 65.5°C in the case of **98DNA_C^CA^G^PA^U^SA^A^OP^** and to 67.7°C for **98DNA_C^CA^G^PA^U^EPh^A^EIn^** respectively (Table 2; Supplementary Figure S17 in SI). Apparently, the stabilizing effect of the ethynyl moieties is overbalanced by the repulsion of the negatively charged modifications and obviously, the effect is stronger in the superanionic DNA compared to the mixed anionic-hydrophobic one. The both-strand-hypermodified superanionic **98DNA_dsC^CA^G^PA^U^SA^A^OP^** appeared to be even less stable and showed a massive *T*_m_ value decrease by 33.4°C in comparison to natural **98DNA**. Nevertheless, despite of decreased stability, all studied samples can be denatured and rehybridized repeatedly. In addition, we have studied the effect of divalent cations (Mg^2+^, Mn^2+^, Zn^2^) on *T*_m_ values of the modified DNA. We found that Mg^2+^ can shield the negative charges more effectively than the other studied cations and increase the *T*_m_ of the duplexes (for details see Supplementary Figures S16, S18–S20 and Supplementary Table S13 in SI).

**Table 2.** Melting temperatures (*T*_m_) and hysteresis of natural and modified DNA determined by UV spectroscopy

DNA title	*T*_m_ (°C)	Hysteresis = *T*_m_– *T*_a_ (°C)	Δ*T*_m_/ modification
**98DNA**	78.6	3.7	–
**98DNA_C^CA^G^PA^U^SA^A^OP^**	65.5	1.7	–0.179
**98DNA_C^CA^G^PA^U^EPh^A^EIn^**	67.7	2.9	–0.149
**98DNA_dsC^CA^G^PA^U^SA^A^OP^**	45.2	3.28	–0.221

**Figure 5.**
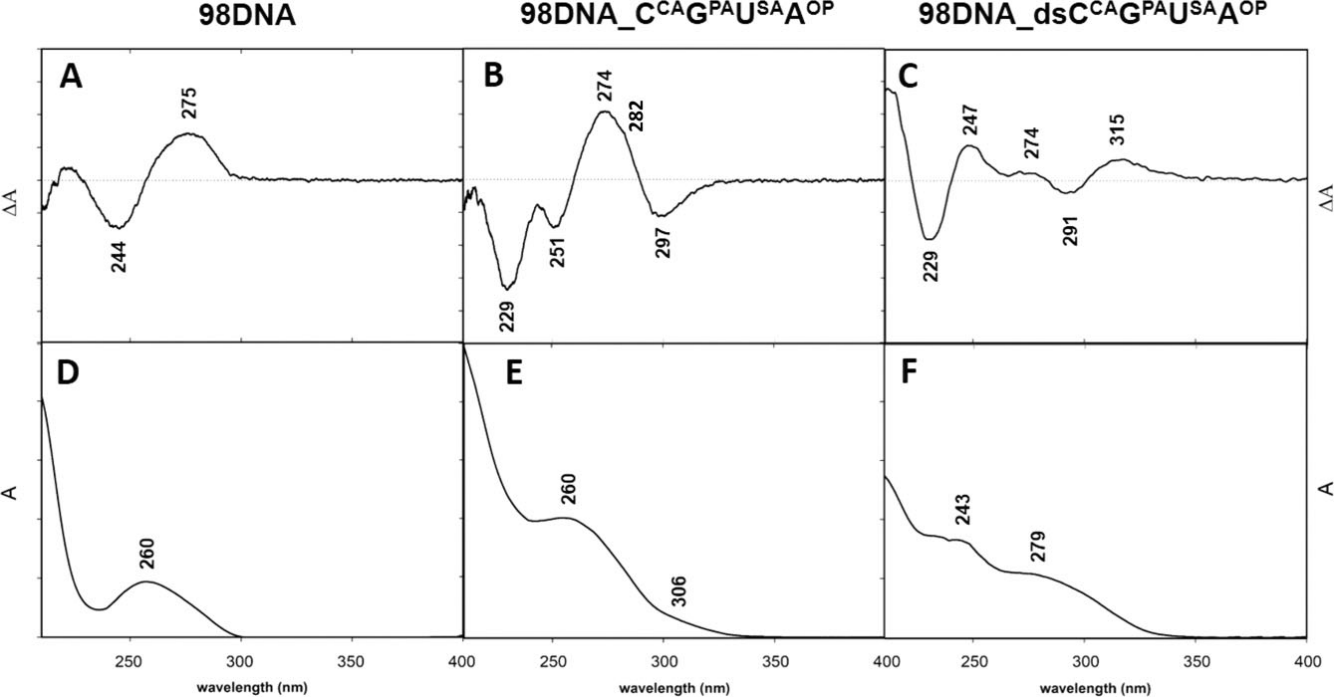
CD spectra (**A–C**) and UV absorption spectra (**D–F**) of **98DNA**, **98DNA_C^CA^G^PA^U^SA^A^OP^**, and **98DNA_dsC^CA^G^PA^U^SA^A^OP^** respectively.

To get deeper understanding of the structure and properties of superanionic DNA, we have performed UV-vis absorption and circular dichroism (CD) spectroscopy studies. The natural 98bp **98DNA** was characterized by the absorption maximum at ∼260 nm and by conservative CD spectrum with maxima at ∼244 nm (–) and ∼277 nm (+) typical for B-form (41) (Figure 5A, D). In the case of **98DNA_C^CA^G^PA^U^SA^A^OP^** the absorption maximum at ∼260 nm, typical for natural DNA, is accompanied by an additional absorption band with lower intensity at ∼306 nm (Figure 5E) that is probably caused by the increased conjugation of ethynyl-linked nucleotides similarly to the previously reported hydrophobic alkyne-linked DNA (22). CD spectrum of **98DNA_C^CA^G^PA^U^SA^A^OP^** was not significantly altered by modifications and showed a positive CD spectral band at ∼274 nm (+), typical for B-type DNA, accompanied by a shoulder at ∼282 nm (+) and by negative bands at ∼229 nm (–), ∼251 nm (–) and ∼297 nm (–) (Figure 5B). On the other hand, UV-vis absorption and CD spectra of the DNA duplex **98DNA_dsC^CA^G^PA^U^SA^A^OP^** containing both strands fully-modified with anionic modifications differed remarkably. The UV-vis spectrum showed absorption maxima at ∼243 nm and at ∼279 nm and no maximum at ∼260 nm (Figure 5F). The CD spectrum was characterized by the following spectral bands: 315 nm (+), 291 nm (–), 274 nm (+), 247 nm (+) and 229 nm (–) (Figure 5C) (CD spectra reflecting changes within **98DNA**, **98DNA_C^CA^G^PA^U^SA^A^OP^**, **98DNA_dsC^CA^G^PA^U^SA^A^OP^** duplexes with increasing temperature are shown in Supplementary Figure S15 in SI). Previously it has been reported that high-density of major-groove modifications into dsDNA can enforce a transition from the right-handed B-type DNA to a left-handed form similar to Z-DNA (19,42). However, accurate conformational analysis of such a heavily modified DNA cannot be based only on CD spectroscopy results. The spectral signature of the DNA backbone is biased by spectral contribution of chromophores (such as alkynes conjugated to nucleobases as well as carboxylic and sulfonic groups). Even though the chromophores are achiral themselves, their mutual orientation and arrangement with respect to the DNA backbone can cause changes in CD spectra (43). Additionally, we have measured CD and UV-vis spectra of mixed duplexes **98DNA_C^CA^G^PA^U^EPh^A^EIn^** and **98DNA_dsC^CA^G^PA^U^EPh^A^EIn^** containing the combination of two hydrophobic and two anionic modifications in either one or both strands (for sequences see Supplementary Table S2 in SI). The obtained CD spectra were similar to those of previously studied hypermodified hydrophobic DNA (22) (for samples preparation see section 3.1 and Supplementary Figure S13 in SI; CD spectra of **98DNA_C^CA^G^PA^U^EPh^A^EIn^** and **98DNA_dsC^CA^G^PA^U^EPh^A^EIn^** are shown in Supplementary Figure S14 in SI).

**Figure 6.**
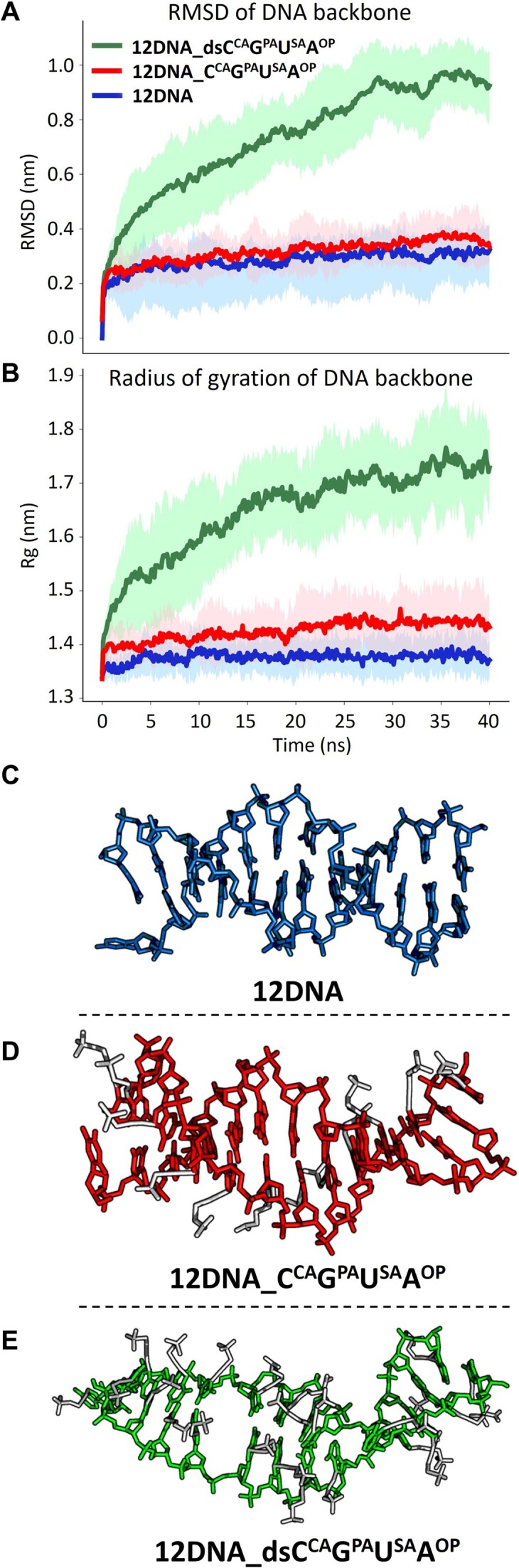
The time evolution of RMSD (**A**) and the radius of gyration (**B**) for **12DNA**, **12DNA_C^CA^G^PA^U^SA^A^OP^**, **12DNA_dsC^CA^G^PA^U^SA^A^OP^**. (C–E) Imaging of the built models in equilibrium: (**C**) **12DNA**; (**D**) **12DNA_C^CA^G^PA^U^SA^A^OP^**; (**E**) **12DNA_dsC^CA^G^PA^U^SA^A^OP^**.

To explain the above-mentioned effects of the anionic modifications on a DNA structure, we performed molecular dynamics (MD) simulation. Due to the limitations of the method, DNA models were built 12bp long. Three 12bp DNA duplexes were designed: a natural **12DNA**, **12DNA_C^CA^G^PA^U^SA^A^OP^** containing one strand fully-modified with anionic modifications, and **12DNA_dsC^CA^G^PA^U^SA^A^OP^** containing both strands fully-modified with anionic modifications (for details see section 4 in SI). In the case of one-strand-modified **12DNA_C^CA^G^PA^U^SA^A^OP^** the time evolution of the root mean square deviation (RMSD) and the radius of gyration (Rg) with respect to a B-DNA reference structure do not differ statistically from the natural **12DNA** (Figure 6A, B), meaning that DNA with one fully-modified strand preserves the B-conformation. Conversely, the RMSD and Rg values for **12DNA_dsC^CA^G^PA^U^SA^A^OP^** are significantly higher indicating that the molecule adopts a different conformation than unmodified **12DNA**. Moreover, a helical structure is considerably loosened in **12DNA_dsC^CA^G^PA^U^SA^A^OP^**, as shown in Figure 6E. Thus, according to the combination of MD simulation results in accord with the observed changes in CD spectra and the low *T*_m_ of the DNA duplex, **12DNA_dsC^CA^G^PA^U^SA^A^OP^** containing both strands fully-modified with anionic modifications adopts a conformation different from B-form DNA but still preserves a loosened right-handed helix.

## Conclusions

We designed and synthesized a full set of all four dNTPs bearing different anionic groups attached through an alkyne tether to position 5 of pyrimidines or to position 7 of 7-deazapurines. They were all good substrates for Vent(exo^–^) and KOD XL DNA polymerases in PEX and were used in the enzymatic synthesis of DNA strands containing one, two, three or even all four modified nucleotides with up to 125 anionic modifications in a row. Also, a combination of two anionic dNTPs (**dC^CA^TP** and **dG^PA^TP**) with two previously reported (22) dNTPs bearing hydrophobic moieties (**dU^EPh^TP** and **dA^EIn^TP**) was successfully used in PEX synthesis of mixed hypermodified DNA with the combination of anionic and hydrophobic substituents. In PCR, the exponential amplification through extension of both forward and reverse primer worked mostly for combinations of one or two modified nucleotides. However, for most combinations of three or four modified dNTPs, only linear amplification (aPCR) was observed indicating that the hypermodified superanionic DNA strand is not a good template for the polymerase synthesis of another hypermodified strand. On the other hand, the superanionic hypermodified strand can be replicated with natural nucleotides with good fidelity which allows its re-PCR and sequencing. The possibility to synthesize the hypermodified DNA by PEX or aPCR and to be able to perform its re-PCR followed by correct sequencing is of crucial importance for prospective applications of the anionic nucleotides and superanionic DNA in selection of functional nucleic acids.

The UV, CD, *T*_m_ and MD studies of the influence of the anionic modifications on the structure and stability of DNA revealed that despite the significant destabilization of the duplex due to the increased coulombic repulsion of the polyanionic strands, the hypermodified superanionic DNA strand can still hybridize with complementary natural or with another hypermodified strand. While the duplexes containing one hypermodified anionic and one natural strand still exist in B-form conformation (though with decreased denaturation temperature), the duplex composed of two complementary hypermodified anionic strands adopts a different conformation with still right-handed but loosened helix. These results show the scope and limitations of the enzymatic synthesis and hybridization properties of superanionic or mixed hypermodified oligonucleotides. Since some anionic polymers and nanomaterials have recently been reported to possess antimicrobial (44) or antiviral (45,46) properties and some anionic and amphiphilic polymers were used for delivery of drugs and genes (47–49), there might be potential of design and testing some superanionic nucleic acids in these applications. Moreover, the anionic nucleotides can be combined with other modified nucleotides for selection of functional NA (aptamers, DNAzymes etc.), construction of functionalized origami or other nanostructures or in medicinal chemistry as therapeutic NA. These applications will be further pursued in our labs and reported in due course.

## Supplementary Material

gkad893_Supplemental_File

## Data Availability

The data underlying this article are available in the article and in its online supplementary material. Further data underlying this article are available in Zenodo at https://zenodo.org/record/7998610.
