# Supplementary material for: Superanionic DNA: enzymatic synthesis of hypermodified DNA bearing four different anionic substituents at all four nucleobases
Source: Nucleic Acids Res. 2023 Oct 23;51(21):11428–38. doi: 10.1093/nar/gkad893 (PMC10681718; doi:10.1093/nar/gkad893)
Supplement: gkad893_Supplemental_File [file gkad893_supplemental_file.pdf]

Supporting Information

Superanionic DNA. Enzymatic Synthesis of Hypermodified DNA Bearing Four Different Anionic Substituents at all Four Nucleobases

Natalia Kuprikova,[a,b] Marek Ondruš,[a] Lucie Bednárová,[a] Miguel Riopedre-Fernandez,[a] Lenka Poštová Slavětínská,[a] Veronika Sýkorová,[a] Michal Hocek* [a,b]

a) Institute of Organic Chemistry and Biochemistry, Czech Academy of Sciences, Flemingovo nam. 2, CZ-16000 Prague 6, Czech Republic; hocek@uochb.cas.cz

b) Department of Organic Chemistry, Faculty of Science, Charles University, Hlavova 8, CZ-12843 Prague 2, Czech Republic

Table of Contents

1. Experimental section – organic chemistry 4

1.1. Synthesis of modified triphosphates – Sonogashira cross-coupling 5

2. Experimental section – biochemistry 9

2.1. PEX – Single incorporation (one modified **dN^{R}TP**) 19

2.2. PEX – Multiple incorporation (one modified **dN^{R}TP**) 20

2.3. PEX – Multiple incorporation (two, three and four modified **dN^{R}TP**s) 21

2.4. PEX – Multiple incorporation (**dC^{CA}TP**, **dG^{PA}TP**, **dU^{SA}TP**, **dA^{OP}TP** in various template length) 24

2.5. PEX – Multiple incorporation (**dC^{CA}TP**, **dG^{PA}TP**, **dU^{EPh}TP**, **dA^{EIn}TP** in various template length) 26

2.6. General procedure for ssDNA generation via magnetoseparation 28

2.7. MALDI-TOF measurements 29

2.8. PCR – Multiple incorporation (one modified **dN^{R}TP**) 29

2.9. PCR – Multiple incorporation (two modified **dN^{R}TP**s) 32

2.10. PCR – Multiple incorporation (three modified **dN^{R}TP**s) 33

2.11. PCR – Multiple incorporation (four modified **dN^{R}TP**s) 35

2.12. aPCR – Multiple incorporation (three and four modified **dN^{R}TP**s) 36

2.13. Application of fully-modified ssONs for sequencing 39

2.13.1. Re-PCR of fully-modified ssONs obtained by aPCR 39

2.13.2. aPCR – synthesis of **118ON_C^{CA}G^{PA}U^{SA}A^{OP}** and **118ON_C^{CA}G^{PA}U^{EPh}A^{EIn}** 40

2.13.3. Re-PCR of fully-modified ssONs obtained by PEX 40

2.13.4. PEX – synthesis of **118DNA_C^{CA}G^{PA}U^{SA}A^{OP}** and **118cDNA_C^{CA}G^{PA}U^{SA}A^{OP}** 41

2.13.5. Gel extraction method 42

2.13.6. Re-PCR – synthesis of **118PCR_natural** (for further sequencing) 42

2.14. Sanger sequencing 43

2.15. Results of Sanger sequencing 44

2.15.1. **118PCR_natural_1** 44

2.15.2. **118PCR_natural_2** 45

2.15.3. **118PCR_natural_3** 47

2.15.4. **118PCR_natural_4** 48

3. Experimental section – CD spectroscopy and melting temperatures determination 50
3.1. Preparation of the samples 50
3.1.1. Preparation of **98DNA** 50
3.1.2. Preparation of **98DNA_C^{CA}G^{PA}U^{SA}A^{OP}** 51
3.1.3. Preparation of **98DNA_dsC^{CA}G^{PA}U^{SA}A^{OP}** 51
3.1.4. Preparation of **98DNA_C^{CA}G^{PA}U^{EPh}A^{EIn}** 52
3.1.5. Preparation of **98DNA_dsC^{CA}G^{PA}U^{EPh}A^{EIn}** 52
3.2. Circular dichroism (CD) spectroscopy 54
3.3. UV-VIS spectroscopy 57
4. Molecular Dynamics 60
4.1. Computational models 60
4.2. Simulation protocol 60
4.3. Simulation analysis 61
5. Nuclease degradation experiments 61
6. Copies of MALDI-TOF mass spectra 64
7. Copies of NMR spectra 69
7.1. ^{1}H, ^{13}C and ^{31}P{^{1}H}NMR spectra of **dA^{OP}TP** 69
7.2. ^{1}H, ^{13}C and ^{31}P{^{1}H} NMR spectra of **dG^{PA}TP** 70
7.3. ^{1}H, ^{13}C and ^{31}P{^{1}H} NMR spectra of **dU^{SA}TP** 72
7.4. ^{1}H, ^{13}C and ^{31}P{^{1}H} NMR spectra of **dC^{CA}TP** 73
8. References 75

1. Experimental section – organic chemistry

General remarks

All solvents and reagents were purchased from commercial suppliers and used as received. NMR spectra were recorded on Bruker Avance 400 MHz (400.0 MHz for ^{1}H, 162 MHz for ^{31}P, 100 MHz for ^{13}C) and Bruker Avance 500 MHz (500 MHz for ^{1}H, 125.7 MHz for ^{13}C, 202.3 for ^{31}P) spectrometers from sample solutions in D_2O and CD_3OD. Chemical shifts (in ppm, δ scale) were referenced as follows: D_2O (referenced to t-BuOH as an external reference; 1.25 ppm for ^{1}H NMR and 31.6 ppm for ^{13}C NMR); CD_3OD (referenced to solvent signal: 3.31 ppm for ^{1}H NMR and 49.00 ppm for ^{13}C NMR). ^{31}P chemical shifts were referenced to H_3PO_4 as an external reference. Chemical shifts are given in ppm (δ scale), coupling constants (*J*) in Hz. Reactions were monitored by thin layer chromatography (TLC) on TLC silica gel 60 F254 (Merck Life Science) and detected by UV (254 nm) and by Advion Expression Compact Mass spectrometer connected with Plate Express® TLC Plate Reader using electrospray ionization (ESI). Low- and high-resolution mass spectra were measured on LTQ Orbitrap XL spectrometer (ESI ionization, Thermo Fisher Scientific). All mass spectra were acquired by the MS service at IOCB. Purification of nucleoside triphosphates was performed using HPLC (Waters modular HPLC system) on a column packed with 5 μM C18 reversed phase (XBridge, C18 OBD Prep Column, 130Å). Reactions with triphosphates were analyzed by TLC using IPAV (isopropyl alcohol /NH_4OH /H_2O, ratio 11/7/2) as mobile phase.

Chemicals were of analytical grade. 5-Hexynoic acid, 5-iodo-2'-deoxyuridine and 5-iodo-2'-deoxycytidine were purchased from Fluorochem Ltd. Synthesis and characterization data for 7-iodo-2’-deoxy-7-deazaadenosine 5’-*O*-triphosphate ($dA^{I}TP$)[1], 5-iodo-2’-deoxycytidine 5’-*O*-triphosphate ($dC^{I}TP$)[2], 5-iodo-2’-deoxyuridine 5’-*O*-triphosphate ($dU^{I}TP$)[3], 7-iodo-2’-deoxy-7-deazaguanosine 5’-*O*-triphosphate ($dG^{I}TP$)[4], 3-butynyl-C-phosphonate[5], propargyl sulfonate[6] and propargyl phosphate[7] were reported previously.

1.1. Synthesis of modified triphosphates – Sonogashira cross-coupling

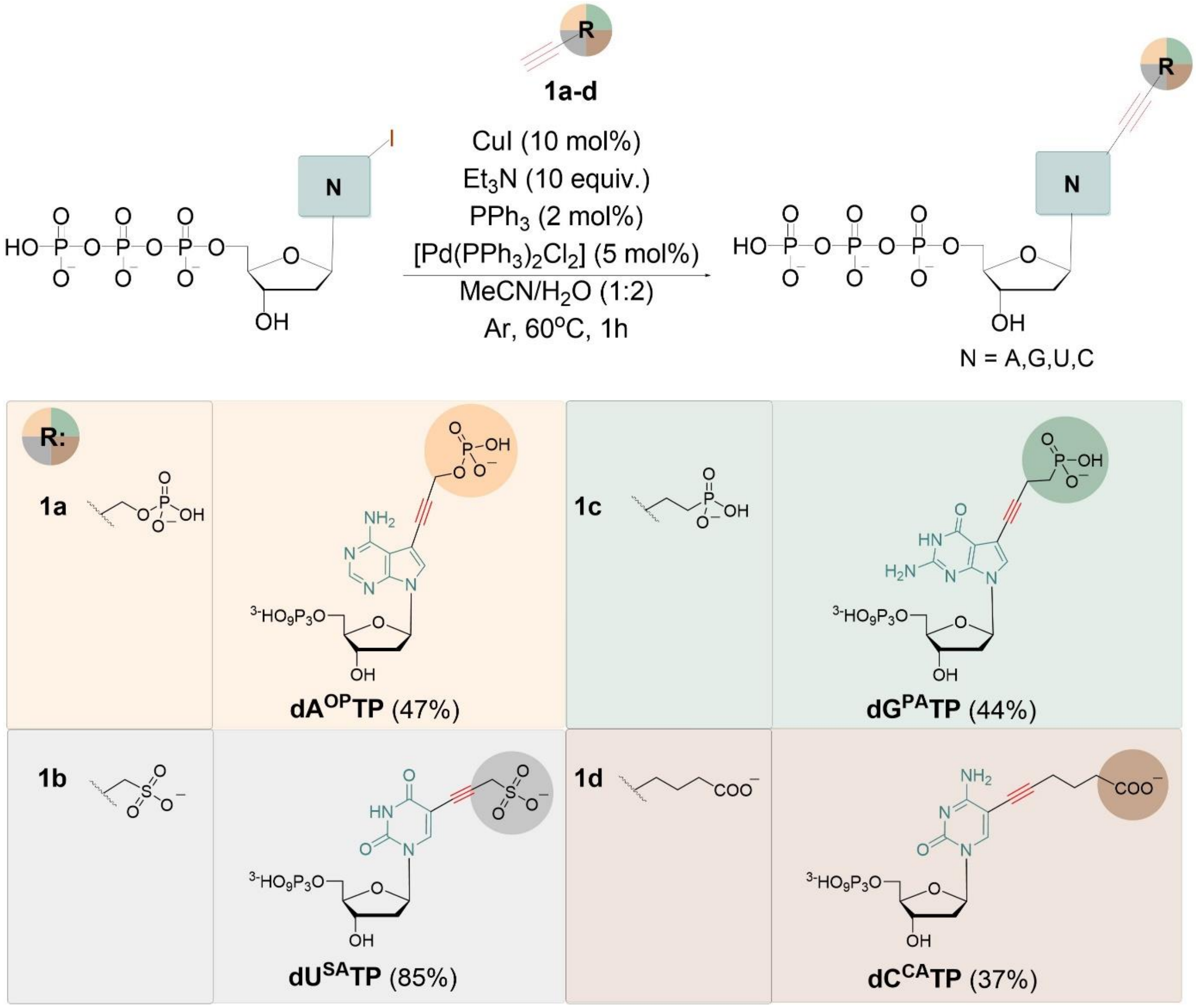

Scheme S1. Reaction scheme of Sonogashira cross-coupling.

Method A. 1:2 mixture of MeCN/H_2O (0.5 mL) followed by Et_3N (10 equiv.) were added through a septum to an argon-purged flask charged with a halogenated nucleoside triphosphate $dN^{I}TP$ (N = U, A, G, C) (1 equiv.), a corresponding alkyne (1.5 equiv.), CuI (10 mol%), PPh_3 (2 mol%) and $[Pd(PPh_3)_2Cl_2]$ (5 mol%). The mixture was stirred for 1 h at 60 °C under argon atmosphere. Solvents were evaporated under vacuum. The product was purified by HPLC with linear gradient of 0.1 M TEAB (triethylammonium bicarbonate) in H_2O to 0.1 M TEAB in H_2O/MeOH (1:1) as eluent in 60 min followed by lyophilization to obtain a solid product.

7-(3-*O*-phosphate-prop-1-yn-1-yl)-2'-deoxy-7-deazaadenosine 5'-*O*-triphosphate (dA^{OP}TP)

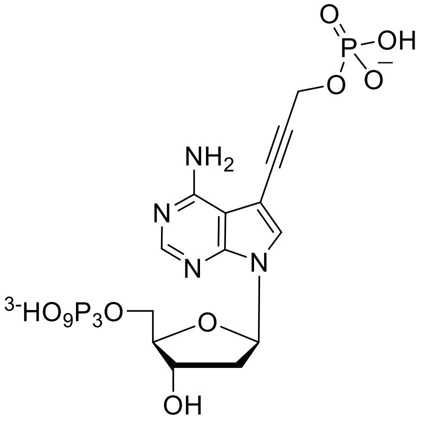

The compound **dA^{OP}TP** was prepared from dA^{I}TP by Method A as described above. The product was isolated as a colorless solid (36 mg, 47%); ^{1}H NMR (500.0 MHz, D_2O): 2.50 (ddd, 1H, J_{gem} = 14.0 Hz, $J_{2'a,1'}$ = 6.3 Hz, $J_{2'a,3'}$ = 3.4 Hz, H-2´a); 2.68 (ddd, 1H, J_{gem} = 14.0 Hz, $J_{2'b,1'}$ = 7.8 Hz, $J_{2'b,3'}$ = 6.2 Hz, H-2´b); 4.17 (ddd, 1H, J_{gem} = 11.3 Hz, $J_{5'a,P}$ = 5.6 Hz, $J_{5'a,4'}$ = 4.1 Hz, H-5´a); 4.21 (ddd, 1H, J_{gem} = 11.3 Hz, $J_{5'b,P}$ = 6.3 Hz, $J_{5'b,4'}$ = 4.1 Hz, H-5´b); 4.26 (m, 1H; H-4´); 4.75 (dt, 1H, $J_{3',2'b}$ = 6.3 Hz, $J_{3',2'a}$ = $J_{3',4'}$ = 3.2 Hz, H-3´); 4.79 (d, 2H, $J_{CH2,P}$ = 10.6 Hz, C≡CH_2); 6.62 (dd, 1H, $J_{1',2'b}$ = 7.8 Hz, $J_{1',2'a}$ = 6.2 Hz, H-1´); 7.82 (s, 1H, H-8); 8.24 (s, 1H, H-2).

^{13}C NMR (125.7 MHz, D_2O): 41.75 (CH_2-2´); 56.78 (d, $J_{C,P}$ = 4.9 Hz, C≡C**C**H_2); 68.46 (d, $J_{C,P}$ = 6.8 Hz, CH_2-5´); 73.99 (CH-3´); 80.33 (**C**≡CCH_2); 86.41 (CH-1´); 88.28 (d, $J_{C,P}$ = 8.9 Hz, CH-4´); 91.80 (d, $J_{C,P}$ = 6.2 Hz, C≡**C**CH_2); 99.91 (C-7); 105.31 (C-5); 130.78 (CH-8); 150.33 (C-4); 150.54 (CH-2); 156.78 (C-6).

^{31}P NMR (202.4 MHz, D_2O): -22.57 (bt, 1P, $J_{\beta,\alpha}$ = $J_{\beta,\gamma}$ = 19.8 Hz, P_β); -10.65 (d, 1P, $J_{\alpha,\beta}$ = 19.9 Hz, P_α); -10.19 (d, 1P, $J_{\gamma,\beta}$ = 19.7 Hz, P_γ); 1.30 (s, 1P, C≡CCH_2O**P**).

HR MS (ESI–) for $C_{14}H_{19}O_{16}N_4P_4$ $[M - H]^-$ calcd.: 622.97520 found: 622.97422.

7-(4-Phosphoryl-but-1-yn-1-yl)-2’-deoxy-7-deazaguanosine 5’-*O*-triphosphate (dG^{PA}TP)

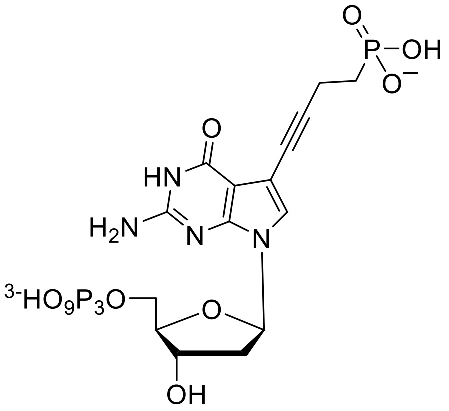

The compound **dG^{PA}TP** was prepared from dG^{I}TP by Method A as described above. The product was isolated as a colorless solid (21 mg, 44%); ^{1}H NMR (500.0 MHz, D_2O): 1.89 – 1.98 (m, 2H, CH_2P); 2.38 (ddd, 1H, J_{gem} = 14.0 Hz, $J_{2'a,1'}$ = 6.2 Hz, $J_{2'a,3'}$ = 3.1 Hz, H-2´a); 2.62 – 2.68 (m, 2H, C≡CCH_2); 2.68 (ddd, 1H, J_{gem} = 14.0 Hz, $J_{2'b,1'}$ = 8.2 Hz, $J_{2'b,3'}$ = 6.3 Hz, H-2´b); 4.11 - 4.17 (m, 2H, H-5´); 4.20 (tdd, 1H; $J_{4',5'}$ = 4.2 Hz, $J_{4',3'}$ = 2.9 Hz, $J_{4',P}$ = 1.0 Hz, H-4´); 4.71 (dt, 1H, $J_{3',2'b}$ = 6.2 Hz, $J_{3',2'a}$ = $J_{3',4'}$ = 3.0 Hz, H-3´); 6.39 (dd, 1H, $J_{1',2'b}$ = 8.2 Hz, $J_{1',2'a}$ = 6.1 Hz, H-1´); 7.27 (s, 1H, H-8).

^{13}C NMR (125.7 MHz, D_2O): 16.03 (d, $J_{C,P}$ = 2.8 Hz, C≡**C**CH_2); 29.34 (d, $J_{C,P}$ = 132.0 Hz, CH_2P); 39.97 (CH_2-2´); 67.72 (d, $J_{C,P}$ = 5.8 Hz, CH_2-5´); 73.31 (CH-3´); 74.98 (d, $J_{C,P}$ = 1.7 Hz, **C**≡CCH_2); 85.13 (CH-1´); 87.01 (d, $J_{C,P}$ = 9.0 Hz, CH-4´); 94.18 (d, $J_{C,P}$ = 20.5 Hz, C≡**C**CH_2); 101.55 (C-7); 102.21 (C-5); 124.89 (CH-8); 152.78 (C-4); 155.19 (C-2); 162.92 (C-6).

^{31}P NMR (202.4 MHz, D_2O): -22.66 (t, 1P, $J_{\beta,\alpha}$ = $J_{\beta,\gamma}$ = 20.1 Hz, P_β); -10.66 (d, 1P, $J_{\alpha,\beta}$ = 20.4 Hz, P_α); -10.20 (d, 1P, $J_{\gamma,\beta}$ = 19.8 Hz, P_γ); 23.75 (s, 1P, CH_2**P**).

HR MS (ESI–) for $C_{15}H_{18}O_{16}N_4Na_3P_4$ [M+3Na – 4H]$^-$ calcd.: 702.93668 found: 702.93555.

5-(3-Sulfo-prop-1-yn-1-yl)-2’-deoxyuridine 5’-*O*-triphosphate (dU^{SA}TP)

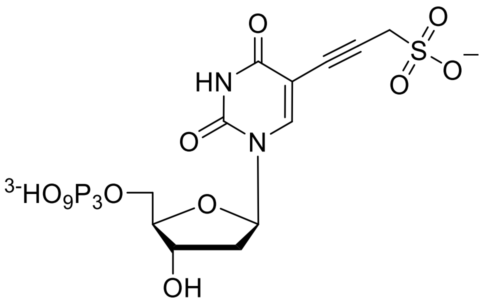

The compound **dU^{SA}TP** was prepared from dU^{I}TP by Method A as described above. The product was isolated as a colorless solid (44 mg, 85%); ^{1}H NMR (500.0 MHz, D_2O): 2.40 (ddd, 1H, J_{gem} = 14.2 Hz, $J_{2'a,1'}$ = 6.6 Hz, $J_{2'a,3'}$ = 4.5 Hz, H-2´a); 2.43 (ddd, 1H, J_{gem} = 14.2 Hz, $J_{2'b,1'}$ = 7.1 Hz, $J_{2'b,3'}$ = 6.0 Hz, H-2´b); 4.07 (s, 2H, CH_2SO_3H); 4.15 - 4.23 (m, 3H; H-4´,5´); 4.61 (ddd, 1H, $J_{3',2'b}$ = 5.9 Hz, $J_{3',2'a}$ = 4.5 Hz, $J_{3',4'}$ = 3.2 Hz, H-3´); 6.25 (t, 1H, $J_{1',2'}$ = 6.8 Hz, H-1´); 8.15 (s, 1H, H-6).

^{13}C NMR (125.7 MHz, D_2O): 40.21 (CH_2-2´); 45.08 (CH_2SO_3H); 67.45 (d, $J_{C,P}$ = 5.6 Hz, CH_2-5´); 72.62 (CH-3´); 77.38 (**C**≡CCH_2); 87.01 (C≡**C**CH_2); 87.38 (d, $J_{C,P}$ = 8.7 Hz, CH-4´); 88.00 (CH-1´); 101.14 (C-5); 147.40 (CH-6); 152.52 (C-2); 166.66 (C-4).

^{31}P NMR (202.4 MHz, D_2O): -22.63 (t, 1P, $J_{\beta,\alpha} = J_{\beta,\gamma}$ = 20.1 Hz, P_β); -10.73 (d, 1P, $J_{\alpha,\beta}$ = 20.3 Hz, P_α); -10.13 (d, 1P, $J_{\gamma,\beta}$ = 19.9 Hz, P_γ).

HR MS (ESI–) for $C_{12}H_{16}O_{17}N_2P_3S$ [M – H]$^{–}$ calcd.: 584.93880 found: 584.93835.

5-(5-Carboxy-pent-1-yn-1-yl)-2’-deoxycytidine 5’-*O*-triphosphate (dC^{CA}TP)

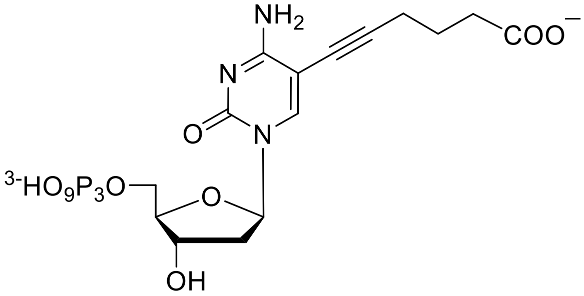

The compound **dC^{CA}TP** was prepared from dC^{I}TP by Method A as described above. The product was isolated as a colorless solid (14 mg, 37%); ^{1}H NMR (500.0 MHz, D_2O): 1.86 (pent, 2H, $J_{CH2,CH2}$ = 7.2 Hz, CH_2C**H**$_2CH_2COOH$); 2.31 (dt, 1H, J_{gem} = 14.1 Hz, $J_{2'a,1'} = J_{2'a,3'}$ = 6.8 Hz, H-2´a); 2.33 (t, 2H, $J_{CH2,CH2}$ = 7.3 Hz, CH_2CH_2C**H**$_2COOH$); 2.43 (ddd, 1H, J_{gem} = 14.1 Hz, $J_{2'b,1'}$ = 6.3 Hz, $J_{2'b,3'}$ = 3.9 Hz, H-2´b); 2.50 (d, 2H, $J_{CH2,CH2}$ = 7.1 Hz, C**H**$_2CH_2CH_2COOH$); 4.14 - 4.24 (m, 3H; H-4´,5´); 4.60 (dt, 1H, $J_{3',2'a}$ = 6.4 Hz, $J_{3',2'b} = J_{3',4'}$ = 3.5 Hz, H-3´); 6.27 (t, 1H, $J_{1',2'}$ = 6.7 Hz, H-1´); 8.03 (s, 1H, H-6).

^{13}C NMR (125.7 MHz, D_2O): 20.71 (**C**$H_2CH_2CH_2COOH$); 26.56 (CH_2**C**H_2CH_2COOH); 38.65 (CH_2CH_2**C**H_2COOH); 40.94 (CH_2-2´); 67.33 (d, $J_{C,P}$ = 5.4 Hz, CH_2-5´); 72.51 (CH-3´); 72.73 (**C**≡CCH_2); 87.33 (d, $J_{C,P}$ = 8.6 Hz, CH-4´); 87.13 (CH-1´); 95.37 (C-5); 99.37 (C≡**C**CH_2); 145.71 (CH-6); 158.03 (C-2); 167.26 (C-4); 184.68 ($CH_2CH_2CH_2$**C**OOH).

^{31}P NMR (202.4 MHz, D_2O): -22.57 (bt, 1P, $J_{\beta,\alpha} = J_{\beta,\gamma} = 19.5$ Hz, P_β); -10.70 (d, 1P, $J_{\alpha,\beta} = 20.0$ Hz, P_α); -9.76 (d, 1P, $J_{\gamma,\beta} = 19.0$ Hz, P_γ).
HR MS (ESI–) for $C_{15}H_{21}O_{15}N_3P_3$ [M – H]$^-$ calcd.: 576.01910 found: 576.01801.

7-(2-(1H-Indol-3-yl)-1-ethyn-1-yl)-2'-deoxyadenosine 5'-*O*-triphosphate (**dA^{EIn}TP**), **5-(2-phenyl-1-ethyn-1-yl)-2'-deoxyuridine 5'-*O*-triphosphate** (**dU^{EPh}TP**), **5-(Pent-1-yn-1-yl)-2'-deoxycytidine 5'-*O*-triphosphate** (**dC^{EAlk}TP**), and **7-(3-methylbut-1-yn-1-yl)-2'-deoxyguanosine 5'-*O*-triphosphate** (**dG^{EiPr}TP**) were synthesized according to the described protocol[8].

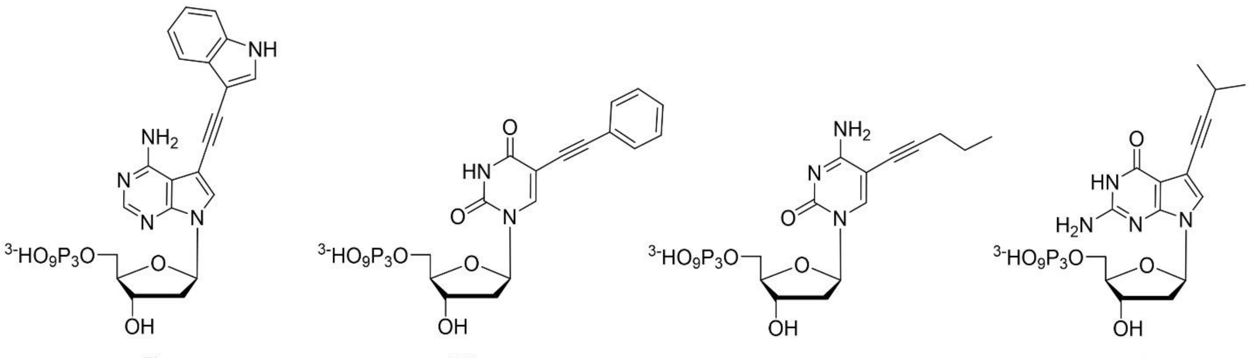

2. Experimental section – biochemistry

General remarks

The MALDI-TOF spectra were measured on a UltrafleXtreme MALDI-TOF/TOF (Bruker) mass spectrometer with a 1 kHz smart beam II laser. The matrix consisted of 3-hydroxypicolinic acid (HPA)/picolinic acid (PA)/ammonium tartrate in ratio 9:1:1. UV-Vis spectra (the concentration of products) were measured at room temperature on NanoDrop1000 (ThermoFisher Scientific). Samples were concentrated on CentriVap Vacuum Concentrator system (Labconco). Synthetic unmodified oligonucleotides (for sequences see Table S1) were purchased from Eurofins Genomics (Germany). Synthetic modified oligonucleotides (3'-sC3-modified templates and 5'-(6-FAM), 5'-Cy5, and Seq+-labelled primers) were purchased from Generi Biotech (Czech Republic). 5'-Dual-biotinylated (2xbio) templates were purchased from Biomers. Natural nucleoside triphosphates (dATP, dGTP, dTTP, dCTP) were purchased from ThermoScientific. KOD XL DNA polymerase and a corresponding polymerase reaction buffer were purchased from

Merck Life Science; Vent(exo-) DNA polymerase and a corresponding polymerase reaction buffer were purchased from New England Biolabs; DNase I and a corresponding polymerase reaction buffer were purchased from ThermoFisher Scientific; Streptavidin magnetic particles (Roche) were purchased from Merck Life Science. Samples for CD measurements and for sequencing were purified using Agencourt AMPure XP magnetic particles (Beckman Coulter Life science - GE Healthcare) and QIAquick PCR Purification Kit (Qiagen). Sanger sequencing was done by SeqMe (Czech Republic). Used kits for gel extraction Pur-A-Lyze Maxi Dialysis Kit (Sigma Aldrich) and Amicon Ultra-0.5 Centrifugal Filters were purchased from Merck Life Science. PCR reactions were performed in a thermal cycler Biometra Trio (Analytic Jena). Milli-Q water was used for all experiments. Samples after PEX and PCR were analyzed by a 12.5% denaturing PAGE (acrylamide/bisacrylamide 19:1, 25% urea) under denaturing conditions (1 h, 50 °C, 1X TBE). Samples after PCR were analyzed by a 2% agarose gel (Serva) containing Gel Red (Biotinum, 10000X in water) in 0.5X TBE buffer (120 V, 75 min) using 6X DNA Gel Loading Dye (60 mM EDTA, 10 mM Tris-HCl (pH 7.6), 60% glycerol, 0.03% bromophenol blue, 0.03% xylene cyanol FF, ThermoFisher Scientific). PAGE stop solution used after PEX reactions contained: 95% [v/v] formamide, 0.5 mM EDTA, 0.025% [w/v] bromophenol blue and 0.025% [w/v] xylene cyanol FF, 0.025% [w/v] SDS in Milli-Q water. PAGE stop solution used for analysis after PCR contained: 95% [v/v] formamide, 0.5 mM EDTA, 0.025% [w/v] bromophenol blue and 0.025% [w/v] SDS in Milli-Q water. All gels were analyzed by fluorescence imaging of 6-FAM and Cy5 labels at 5'-ends using Typhoon FLA 9500 (GE Healthcare Life Sciences). Other chemicals were of analytical grade.

Table S1. List of sequences of primers and templates.

Name	Length (nt)	Sequence (5'→ 3') [a]
Temp$^{\text{Oligo1A}}$	19	CCCTCCCATGCCGCCCATG
Temp$^{\text{Oligo1T}}$	19	CCCACCCATGCCGCCCATG
Temp$^{\text{Oligo1C}}$	19	CCCGCCCATGCCGCCCATG
Temp$^{\text{Oligo1G}}$	19	AAACCCCATGCCGCCCATG
Temp$^{\text{Oligo1A}}$-bio [b]	19	CCCTCCCATGCCGCCCATG
Temp$^{\text{Oligo1T}}$-bio [b]	19	CCCACCCATGCCGCCCATG
Temp$^{\text{Oligo1C}}$-bio [b]	19	CCCGCCCATGCCGCCCATG

Temp$^{\text{Oligo1G}}$-bio [b]	19	AAACCCCATGCCGCCCATG
Temp$^{\text{Prb4basII}}$	31	CTAGCATGAGCTCAGTCCCATGCCGCCCATG
Temp$^{\text{Prb4basII}}$-bio [b]	31	CTAGCATGAGCTCAGTCCCATGCCGCCCATG
Temp$^{\text{MO43}}$	43	CATGAGCTCAGTCTAGCATGAGCTCAGTCCC ATGCCGCCCATG
Temp$^{\text{MO61}}$	61	GACATCATGAGAGACATCGCCTAGCATGAG CTAGCATGAGCTCAGTCCCATGCCGCCCATG
Temp$^{\text{FVL-A}}$	98	GACATCATGAGAGACATCGCCTCTGGGCTA ATAGGACTACTTCTAATCTGTAAGAGCAGAT CCCTGGACAGGCAAGGAATACAGGTATTTT GTCCTTG
Temp$^{\text{FVL-A}}$-sC3 [c]	98	GACATCATGAGAGACATCGCCTCTGGGCTA ATAGGACTACTTCTAATCTGTAAGAGCAGAT CCCTGGACAGGCAAGGAATACAGGTATTTT GTCCTTG
Temp$^{\text{FVL-A_comp}}$	98	CAAGGACAAAATACCTGTATTCCTTGCCTGT CCAGGGATCTGCTCTTACAGATTAGAAGTAG TCCTATTAGCCCAGAGGCGATGTCTCTCATG ATGTC
Temp$^{\text{FVL-A_comp}}$-sC3 [c]	98	CAAGGACAAAATACCTGTATTCCTTGCCTGT CCAGGGATCTGCTCTTACAGATTAGAAGTAG TCCTATTAGCCCAGAGGCGATGTCTCTCATG ATGTC
Temp$^{\text{MO120}}$	120	GACATCATGAGAGACATCGCCTAGCATGAG CTCAGTCTAGCATGAGCTCAGTCTAGCATGA GCTCAGTCTAGCATGAGCTCAGTCTAGCATG AGCAAGGAATACAGGTATTTTGTCCTTG
Temp$^{\text{MO150}}$	150	GACATCATGAGAGACATCGCCTAGCATGAG CTCAGTCTAGCATGAGCTCAGTCTAGCATGA GCTCAGTCTAGCATGAGCTCAGTCTAGCATG

		AGCTCAGTCTAGCATGAGCTCAGTCTAGCAT GAAAGGAATACAGGTATTTTGTCCTTG
Prim$^{\text{248short}}$	15	CATGGGCGGCATGGG
Prim$^{\text{248short}}$-FAM [d]	15	CATGGGCGGCATGGG
Prim$^{\text{LT25TH}}$	25	CAAGGACAAAATACCTGTATTCCTT
Prim$^{\text{LT25TH}}$-FAM [d]	25	CAAGGACAAAATACCTGTATTCCTT
Prim$^{\text{L20}}$	20	GACATCATGAGAGACATCGC
Prim$^{\text{L20}}$-Cy5 [e]	20	GACATCATGAGAGACATCGC
Prim$^{\text{L20}}$-Seq+ [f]	20+	$(N)_x$GACATCATGAGAGACATCGC
Prim$^{\text{Flank-LT25TH}}$-FAM [d]	45	CATTCGGCTGCTCTTGATTTCAAGGACAAAA TACCTGTATTCCTT
Prim$^{\text{Flank-L20}}$-FAM [c]	40	CATTCGGCTGCTCTTGATTTGACATCATGAG AGACATCGC
Prim^{Flank}	20	CATTCGGCTGCTCTTGATTT
Prim^{Flank}-FAM [d]	20	CATTCGGCTGCTCTTGATTT
Prim^{Flank}-Seq+ [f]	20+	$(N)_x$GACATCATGAGAGACATCGC

[a] in the template ONs the segments forming duplex with the primer are underlined; [b] 5'-dual-biotinylated; [c] 3'-sC3 (three carbon spacer); [d] 5'-(6-FAM)-labelled, [e] 5'-Cy5-labelled, [f] extended at 5'-end with unknown sequence

Table S2. List of synthesized ssONs/dsDNAs.

Name	Sequence (5'→ 3') [a]
**19ON_G^{PA}**	CATGGGCGGCATGGGG^{PA}TTT
**19ON_A^{OP}**	CATGGGCGGCATGGGA^{OP}GGG
**19ON_U^{SA}**	CATGGGCGGCATGGGU^{SA}GGG
**19ON_C^{CA}**	CATGGGCGGCATGGGC^{CA}GGG
**19DNA_G^{PA}** [b]	CATGGGCGGCATGGGG^{PA}TTT
**19DNA_A^{OP}** [b]	CATGGGCGGCATGGGA^{OP}GGG
**19DNA_U^{SA}** [b]	CATGGGCGGCATGGGU^{SA}GGG
**19DNA_C^{CA}** [b]	CATGGGCGGCATGGGC^{CA}GGG

**31ON** [b]	CATGGGCGGCATGGGACTGAGCTCATGCTAG
**31ON_G^{PA}**	CATGGGCGGCATGGGACTG^{PA}AG^{PA}CTCATG^{PA}CTAG^{PA}
**31ON_A^{OP}**	CATGGGCGGCATGGGA^{OP}CTGA^{OP}GCTCA^{OP}TGCTA^{OP}G
**31ON_U^{SA}**	CATGGGCGGCATGGGACU^{SA}GAGCU^{SA}CAU^{SA}GCU^{SA}AG
**31ON_C^{CA}**	CATGGGCGGCATGGGAC^{CA}TGAGC^{CA}TC^{CA}ATGC^{CA}TAG
**31DNA_G^{PA}** [b]	CATGGGCGGCATGGGACTG^{PA}AG^{PA}CTCATG^{PA}CTAG^{PA}
**31DNA_A^{OP}** [b]	CATGGGCGGCATGGGA^{OP}CTGA^{OP}GCTCA^{OP}TGCTA^{OP}G
**31DNA_U^{SA}** [b]	CATGGGCGGCATGGGACU^{SA}GAGCU^{SA}CAU^{SA}GCU^{SA}AG
**31DNA_C^{CA}** [b]	CATGGGCGGCATGGGAC^{CA}TGAGC^{CA}TC^{CA}ATGC^{CA}TAG
**31DNA_C^{CA}A^{OP}** [b]	CATGGGCGGCATGGGA^{OP}C^{CA}TGA^{OP}GC^{CA}TC^{CA}A^{OP}TGC^{CA}TA^{OP}G
**31DNA_C^{CA}G^{PA}** [b]	CATGGGCGGCATGGGAC^{CA}TG^{PA}AG^{PA}C^{CA}TC^{CA}ATG^{PA}C^{CA}TAG^{PA}
**31DNA_C^{CA}U^{SA}** [b]	CATGGGCGGCATGGGAC^{CA}U^{SA}GAGC^{CA}U^{SA}C^{CA}AU^{SA}GC^{CA}U^{SA}AG
**31DNA_A^{OP}G^{PA}** [b]	CATGGGCGGCATGGGA^{OP}CTG^{PA}A^{OP}G^{PA}CTCA^{OP}TG^{PA}CTA^{OP}G^{PA}
**31DNA_A^{OP}U^{SA}** [b]	CATGGGCGGCATGGGA^{OP}CU^{SA}GA^{OP}GCU^{SA}CA^{OP}U^{SA}GCU^{SA}A^{OP}G
**31DNA_G^{PA}U^{SA}** [b]	CATGGGCGGCATGGGACU^{SA}G^{PA}AG^{PA}CU^{SA}CAU^{SA}G^{PA}CU^{SA}AG^{PA}
**31DNA_C^{CA}A^{OP}G^{PA}** [b]	CATGGGCGGCATGGGA^{OP}C^{CA}TG^{PA}A^{OP}G^{PA}C^{CA}TC^{CA}A^{OP}TG^{PA}C^{CA}TA^{OP}G^{PA}
**31DNA_C^{CA}A^{OP}U^{SA}** [b]	CATGGGCGGCATGGGA^{OP}C^{CA}U^{SA}GA^{OP}GC^{CA}U^{SA}C^{CA}A^{OP}U^{SA}GC^{CA}U^{SA}A^{OP}G
**31DNA_C^{CA}G^{PA}U^{SA}** [b]	CATGGGCGGCATGGGAC^{CA}U^{SA}G^{PA}AG^{PA}C^{CA}U^{SA}C^{CA}AU^{SA}G^{PA}C^{CA}U^{SA}AG^{PA}
**31DNA_A^{OP}G^{PA}U^{SA}** [b]	CATGGGCGGCATGGGA^{OP}CU^{SA}G^{PA}A^{OP}G^{PA}CU^{SA}CA^{OP}U^{SA}G^{PA}CU^{SA}A^{OP}G^{PA}
**31ON_C^{CA}G^{PA}U^{SA}A^{OP}**	CATGGGCGGCATGGGA^{OP}C^{CA}U^{SA}G^{PA}A^{OP}G^{PA}C^{CA}U^{SA}C^{CA}A^{OP}U^{SA}G^{PA}C^{CA}U^{SA}A^{OP}G^{PA}

**31DNA_C^{CA}G^{PA}U^{SA}A^{OP}**	CATGGGCGGCATGGGA^{OP}C^{CA}U^{SA}G^{PA}A^{OP}G^{PA}C^{CA}U^{SA}C^{CA}A^{OP}U^{SA}G^{PA}C^{CA}U^{SA}A^{OP}G^{PA}
**31DNA_C^{CA}G^{PA}U^{SA}A^{OP}** [b]	CATGGGCGGCATGGGA^{OP}C^{CA}U^{SA}G^{PA}A^{OP}G^{PA}C^{CA}U^{SA}C^{CA}A^{OP}U^{SA}G^{PA}C^{CA}U^{SA}A^{OP}G^{PA}
**31ON_C^{CA}G^{PA}U^{EPh}A^{EIn}**	CATGGGCGGCATGGGA^{EIn}C^{CA}U^{EPh}G^{PA}A^{EIn}G^{PA}C^{CA}U^{EPh}C^{CA}A^{EIn}U^{EPh}G^{PA}C^{CA}U^{EPh}A^{EIn}G^{PA}
**31DNA_C^{CA}G^{PA}U^{EPh}A^{EIn}**	CATGGGCGGCATGGGA^{EIn}C^{CA}U^{EPh}G^{PA}A^{EIn}G^{PA}C^{CA}U^{EPh}C^{CA}A^{EIn}U^{EPh}G^{PA}C^{CA}U^{EPh}A^{EIn}G^{PA}
**31DNA_C^{CA}G^{PA}U^{EPh}A^{EIn}** [b]	CATGGGCGGCATGGGA^{EIn}C^{CA}U^{EPh}G^{PA}A^{EIn}G^{PA}C^{CA}U^{EPh}C^{CA}A^{EIn}U^{EPh}G^{PA}C^{CA}U^{EPh}A^{EIn}G^{PA}
**31ON_C^{EAlk}G^{EiPr}U^{EPh}A^{EIn}** [b]	CATGGGCGGCATGGGA^{EIn}C^{EAlk}U^{EPh}G^{EiPr}A^{EIn}G^{EiPr}C^{EAlk}U^{EPh}C^{EAlk}A^{EIn}U^{EPh}G^{EiPr}C^{EAlk}U^{EPh}A^{EIn}G^{EiPr}
**43DNA_C^{CA}G^{PA}U^{SA}A^{OP}** [b]	CATGGGCGGCATGGGA^{OP}C^{CA}U^{SA}G^{PA}A^{OP}G^{PA}C^{CA}U^{SA}C^{CA}A^{OP}U^{SA}G^{PA}C^{CA}U^{SA}A^{OP}G^{PA}A^{OP}C^{CA}U^{SA}G^{PA}A^{OP}G^{PA}C^{CA}U^{SA}C^{CA}A^{OP}U^{SA}G^{PA}
**43DNA_C^{CA}G^{PA}U^{EPh}A^{EIn}** [b]	CATGGGCGGCATGGGA^{EIn}C^{CA}U^{EPh}G^{PA}A^{EIn}G^{PA}C^{CA}U^{EPh}C^{CA}A^{EIn}U^{EPh}G^{PA}C^{CA}U^{EPh}A^{EIn}G^{PA}A^{EIn}C^{CA}U^{EPh}G^{PA}A^{EIn}G^{PA}C^{CA}U^{EPh}C^{CA}A^{EIn}U^{EPh}G^{PA}
**61DNA_C^{CA}G^{PA}U^{SA}A^{OP}** [b]	CATGGGCGGCATGGGA^{OP}C^{CA}U^{SA}G^{PA}A^{OP}G^{PA}C^{CA}U^{SA}C^{CA}A^{OP}U^{SA}G^{PA}C^{CA}U^{SA}A^{OP}G^{PA}C^{CA}U^{SA}C^{CA}A^{OP}U^{SA}G^{PA}C^{CA}U^{SA}A^{OP}G^{PA}G^{PA}C^{CA}G^{PA}A^{OP}U^{SA}G^{PA}U^{SA}C^{CA}U^{SA}C^{CA}U^{SA}C^{CA}A^{OP}U^{SA}G^{PA}A^{OP}U^{SA}G^{PA}U^{SA}C^{CA}
**61DNA_C^{CA}G^{PA}U^{EPh}A^{EIn}** [b]	CATGGGCGGCATGGGA^{EIn}C^{CA}U^{EPh}G^{PA}A^{EIn}G^{PA}C^{CA}U^{EPh}C^{CA}A^{EIn}U^{EPh}G^{PA}C^{CA}U^{EPh}A^{EIn}G^{PA}C^{CA}U^{EPh}C^{CA}A^{EIn}U^{EPh}G^{PA}C^{CA}U^{EPh}A^{EIn}G^{PA}G^{PA}C^{CA}G^{PA}A^{EIn}U^{EPh}G^{PA}U^{EPh}C^{CA}U^{EPh}C^{CA}U^{EPh}C^{CA}A^{EIn}U^{EPh}G^{PA}A^{EIn}U^{EPh}G^{PA}U^{EPh}C^{CA}
**98ON_C^{CA}G^{PA}U^{SA}A^{OP}**	CAAGGACAAAATACCTGTATTCCTTG^{PA}C^{CA}C^{CA}U^{SA}G^{PA}U^{SA}C^{CA}C^{CA}A^{OP}G^{PA}G^{PA}G^{PA}A^{OP}U^{SA}C^{CA}U^{SA}G^{PA}C^{CA}U^{SA}C^{CA}U^{SA}U^{SA}A^{OP}C^{CA}A^{OP}G^{PA}A^{OP}U^{SA}U^{SA}A^{OP}G^{PA}A^{OP}A^{OP}G^{PA}U^{SA}A^{OP}G^{PA}U^{SA}C^{CA}C^{CA}U^{SA}A^{OP}U^{SA}U^{SA}A^{OP}G^{PA}C^{CA}C^{CA}C^{CA}A^{OP}G^{PA}A^{OP}G^{PA}G^{PA}C^{CA}G^{PA}A^{OP}U^{SA}G^{PA}U^{SA}C^{CA}U^{SA}C^{CA}U^{SA}C^{CA}A^{OP}U^{SA}G^{PA}A^{OP}U^{SA}G^{PA}U^{SA}C^{CA}
**98ON_C^{CA}G^{PA}U^{SA}A^{OP}** [b]	CAAGGACAAAATACCTGTATTCCTTG^{PA}C^{CA}C^{CA}U^{SA}G^{PA}U^{SA}C^{CA}C^{CA}A^{OP}G^{PA}G^{PA}G^{PA}A^{OP}U^{SA}C^{CA}U^{SA}G^{PA}C^{CA}U^{SA}C^{CA}U^{SA}U^{SA}A^{OP}C^{CA}A^{OP}G^{PA}A^{OP}U^{SA}U^{SA}A^{OP}G^{PA}A^{OP}A^{OP}G^{PA}U^{SA}A^{OP}G^{PA}U^{SA}C^{CA}C^{CA}U^{SA}A^{OP}U^{SA}U^{SA}A^{OP}G^{PA}C^{CA}C^{CA}C^{CA}A^{OP}G^{PA}A^{OP}G^{PA}G^{PA}C^{CA}G^{PA}A^{OP}U^{SA}G^{PA}U^{SA}C^{CA}U^{SA}C^{CA}U^{SA}C^{CA}A^{OP}U^{SA}G^{PA}A^{OP}U^{SA}G^{PA}U^{SA}C^{CA}
**98DNA_C^{CA}G^{PA}U^{SA}A^{OP}**	CAAGGACAAAATACCTGTATTCCTTG^{PA}C^{CA}C^{CA}U^{SA}G^{PA}U^{SA}C^{CA}C^{CA}A^{OP}G^{PA}G^{PA}G^{PA}A^{OP}U^{SA}C^{CA}U^{SA}G^{PA}C^{CA}U^{SA}C^{CA}U^{SA}U^{SA}A^{O}

^{P}C^{CA}A^{OP}G^{PA}A^{OP}U^{SA}U^{SA}A^{OP}G^{PA}A^{OP}A^{OP}G^{PA}U^{SA}A^{OP}G^{PA}U^{SA}C^{CA}C
^{CA}U^{SA}A^{OP}U^{SA}U^{SA}A^{OP}G^{PA}C^{CA}C^{CA}C^{CA}A^{OP}G^{PA}A^{OP}G^{PA}G^{PA}C^{CA}G^{PA}
A^{OP}U^{SA}G^{PA}U^{SA}C^{CA}U^{SA}C^{CA}U^{SA}C^{CA}A^{OP}U^{SA}G^{PA}A^{OP}U^{SA}G^{PA}U^{SA}C^{C}
^{A}

98DNA_C^{CA}G^{PA}U^{SA}A^{OP} [b]

$\underline{\text{CAAGGACAAAATACCTGTATTCCTT}}$G^{PA}C^{CA}C^{CA}U^{SA}G^{PA}U^{SA}
C^{CA}C^{CA}A^{OP}G^{PA}G^{PA}G^{PA}A^{OP}U^{SA}C^{CA}U^{SA}G^{PA}C^{CA}U^{SA}C^{CA}U^{SA}U^{SA}A^{O}
^{P}C^{CA}A^{OP}G^{PA}A^{OP}U^{SA}U^{SA}A^{OP}G^{PA}A^{OP}A^{OP}G^{PA}U^{SA}A^{OP}G^{PA}U^{SA}C^{CA}C
^{CA}U^{SA}A^{OP}U^{SA}U^{SA}A^{OP}G^{PA}C^{CA}C^{CA}C^{CA}A^{OP}G^{PA}A^{OP}G^{PA}G^{PA}C^{CA}G^{PA}
A^{OP}U^{SA}G^{PA}U^{SA}C^{CA}U^{SA}C^{CA}U^{SA}C^{CA}A^{OP}U^{SA}G^{PA}A^{OP}U^{SA}G^{PA}U^{SA}C^{C}
^{A}

98cON_C^{CA}G^{PA}U^{SA}A^{OP}

$\underline{\text{GACATCATGAGAGACATCGC}}$C^{CA}U^{SA}C^{CA}U^{SA}G^{PA}G^{PA}G^{PA}C^{CA}
U^{SA}A^{OP}A^{OP}U^{SA}A^{OP}G^{PA}G^{PA}A^{OP}C^{CA}U^{SA}A^{OP}C^{CA}U^{SA}U^{SA}C^{CA}U^{SA}A^{O}
^{P}A^{OP}U^{SA}C^{CA}U^{SA}G^{PA}U^{SA}A^{OP}A^{OP}G^{PA}A^{OP}G^{PA}C^{CA}A^{OP}G^{PA}A^{OP}U^{SA}C
^{CA}C^{CA}C^{CA}U^{SA}G^{PA}G^{PA}A^{OP}C^{CA}A^{OP}G^{PA}G^{PA}C^{CA}A^{OP}A^{OP}G^{PA}G^{PA}A^{OP}
A^{OP}U^{SA}A^{OP}C^{CA}A^{OP}G^{PA}G^{PA}U^{SA}A^{OP}U^{SA}U^{SA}U^{SA}U^{SA}G^{PA}U^{SA}C^{CA}C^{C}
^{A}U^{SA}U^{SA}G^{PA}

98cDNA_C^{CA}G^{PA}U^{SA}A^{OP}

$\underline{\text{GACATCATGAGAGACATCGC}}$C^{CA}U^{SA}C^{CA}U^{SA}G^{PA}G^{PA}G^{PA}C^{CA}
U^{SA}A^{OP}A^{OP}U^{SA}A^{OP}G^{PA}G^{PA}A^{OP}C^{CA}U^{SA}A^{OP}C^{CA}U^{SA}U^{SA}C^{CA}U^{SA}A^{O}
^{P}A^{OP}U^{SA}C^{CA}U^{SA}G^{PA}U^{SA}A^{OP}A^{OP}G^{PA}A^{OP}G^{PA}C^{CA}A^{OP}G^{PA}A^{OP}U^{SA}C
^{CA}C^{CA}C^{CA}U^{SA}G^{PA}G^{PA}A^{OP}C^{CA}A^{OP}G^{PA}G^{PA}C^{CA}A^{OP}A^{OP}G^{PA}G^{PA}A^{OP}
A^{OP}U^{SA}A^{OP}C^{CA}A^{OP}G^{PA}G^{PA}U^{SA}A^{OP}U^{SA}U^{SA}U^{SA}U^{SA}G^{PA}U^{SA}C^{CA}C^{C}
^{A}U^{SA}U^{SA}G^{PA}

98DNA_dsC^{CA}G^{PA}U^{SA}A^{OP} [c]

$\underline{\text{CAAGGACAAAATACCTGTATTCCTT}}$G^{PA}C^{CA}C^{CA}U^{SA}G^{PA}U^{SA}
C^{CA}C^{CA}A^{OP}G^{PA}G^{PA}G^{PA}A^{OP}U^{SA}C^{CA}U^{SA}G^{PA}C^{CA}U^{SA}C^{CA}U^{SA}U^{SA}A^{O}
^{P}C^{CA}A^{OP}G^{PA}A^{OP}U^{SA}U^{SA}A^{OP}G^{PA}A^{OP}A^{OP}G^{PA}U^{SA}A^{OP}G^{PA}U^{SA}C^{CA}C
^{CA}U^{SA}A^{OP}U^{SA}U^{SA}A^{OP}G^{PA}C^{CA}C^{CA}C^{CA}A^{OP}G^{PA}A^{OP}G^{PA}G^{PA}C^{CA}G^{PA}
A^{OP}U^{SA}G^{PA}U^{SA}C^{CA}U^{SA}C^{CA}U^{SA}C^{CA}A^{OP}U^{SA}G^{PA}A^{OP}U^{SA}G^{PA}U^{SA}C^{C}
^{A}

98ON_C^{CA}G^{PA}U^{EPh}A^{EIn}

$\underline{\text{CAAGGACAAAATACCTGTATTCCTT}}$G^{PA}C^{CA}C^{CA}U^{EPh}G^{PA}U^{E}
^{Ph}C^{CA}C^{CA}A^{EIn}G^{PA}G^{PA}G^{PA}A^{EIn}U^{EPh}C^{CA}U^{EPh}G^{PA}C^{CA}U^{EPh}C^{CA}U^{EPh}U
^{EPh}A^{EIn}C^{CA}A^{EIn}G^{PA}A^{EIn}U^{EPh}U^{EPh}A^{EIn}G^{PA}A^{EIn}A^{EIn}G^{PA}U^{EPh}A^{EIn}G^{PA}
U^{EPh}C^{CA}C^{CA}U^{EPh}A^{EIn}U^{EPh}U^{EPh}A^{EIn}G^{PA}C^{CA}C^{CA}C^{CA}A^{EIn}G^{PA}A^{EIn}G^{P}
^{A}G^{PA}C^{CA}G^{PA}A^{EIn}U^{EPh}G^{PA}U^{EPh}C^{CA}U^{EPh}C^{CA}U^{EPh}C^{CA}A^{EIn}U^{EPh}G^{PA}A
^{EIn}U^{EPh}G^{PA}U^{EPh}C^{CA}

98DNA_C^{CA}G^{PA}U^{EPh}A^{EIn}

$\underline{\text{CAAGGACAAAATACCTGTATTCCTT}}$G^{PA}C^{CA}C^{CA}U^{EPh}G^{PA}U^{E}
^{Ph}C^{CA}C^{CA}A^{EIn}G^{PA}G^{PA}G^{PA}A^{EIn}U^{EPh}C^{CA}U^{EPh}G^{PA}C^{CA}U^{EPh}C^{CA}U^{EPh}U
^{EPh}A^{EIn}C^{CA}A^{EIn}G^{PA}A^{EIn}U^{EPh}U^{EPh}A^{EIn}G^{PA}A^{EIn}A^{EIn}G^{PA}U^{EPh}A^{EIn}G^{PA}
U^{EPh}C^{CA}C^{CA}U^{EPh}A^{EIn}U^{EPh}U^{EPh}A^{EIn}G^{PA}C^{CA}C^{CA}C^{CA}A^{EIn}G^{PA}A^{EIn}G^{P}
^{A}G^{PA}C^{CA}G^{PA}A^{EIn}U^{EPh}G^{PA}U^{EPh}C^{CA}U^{EPh}C^{CA}U^{EPh}C^{CA}A^{EIn}U^{EPh}G^{PA}A
^{EIn}U^{EPh}G^{PA}U^{EPh}C^{CA}

98DNA_C^{CA}G^{PA}U^{EPh}A^{EIn} [b]

$\underline{\text{CAAGGACAAAATACCTGTATTCCTT}}$G^{PA}C^{CA}C^{CA}U^{EPh}G^{PA}U^{E}
^{Ph}C^{CA}C^{CA}A^{EIn}G^{PA}G^{PA}G^{PA}A^{EIn}U^{EPh}C^{CA}U^{EPh}G^{PA}C^{CA}U^{EPh}C^{CA}U^{EPh}U
^{EPh}A^{EIn}C^{CA}A^{EIn}G^{PA}A^{EIn}U^{EPh}U^{EPh}A^{EIn}G^{PA}A^{EIn}A^{EIn}G^{PA}U^{EPh}A^{EIn}G^{PA}
U^{EPh}C^{CA}C^{CA}U^{EPh}A^{EIn}U^{EPh}U^{EPh}A^{EIn}G^{PA}C^{CA}C^{CA}C^{CA}A^{EIn}G^{PA}A^{EIn}G^{P}
^{A}G^{PA}C^{CA}G^{PA}A^{EIn}U^{EPh}G^{PA}U^{EPh}C^{CA}U^{EPh}C^{CA}U^{EPh}C^{CA}A^{EIn}U^{EPh}G^{PA}A
^{EIn}U^{EPh}G^{PA}U^{EPh}C^{CA}

**98cON_$\mathrm{C^{CA}G^{PA}U^{EPh}A^{EIn}}$**	$\mathrm{GACATCATGAGAGACATCGCC^{CA}U^{EPh}C^{CA}U^{EPh}G^{PA}G^{PA}G^{PA}C^{CA}U^{EPh}A^{EIn}A^{EIn}U^{EPh}A^{EIn}G^{PA}G^{PA}A^{EIn}C^{CA}U^{EPh}A^{EIn}C^{CA}U^{EPh}U^{EPh}C^{CA}U^{EPh}A^{EIn}A^{EIn}U^{EPh}C^{CA}U^{EPh}G^{PA}U^{EPh}A^{EIn}A^{EIn}G^{PA}A^{EIn}G^{PA}C^{CA}A^{EIn}G^{PA}A^{EIn}U^{EPh}C^{CA}C^{CA}C^{CA}U^{EPh}G^{PA}G^{PA}A^{EIn}C^{CA}A^{EIn}G^{PA}G^{PA}C^{CA}A^{EIn}A^{EIn}G^{PA}G^{PA}A^{EIn}A^{EIn}U^{EPh}A^{EIn}C^{CA}A^{EIn}G^{PA}G^{PA}U^{EPh}A^{EIn}U^{EPh}U^{EPh}U^{EPh}U^{EPh}G^{PA}U^{EPh}C^{CA}C^{CA}U^{EPh}U^{EPh}G^{PA}}$
**98cDNA_$\mathrm{C^{CA}G^{PA}U^{EPh}A^{EIn}}$**	$\mathrm{GACATCATGAGAGACATCGCC^{CA}U^{EPh}C^{CA}U^{EPh}G^{PA}G^{PA}G^{PA}C^{CA}U^{EPh}A^{EIn}A^{EIn}U^{EPh}A^{EIn}G^{PA}G^{PA}A^{EIn}C^{CA}U^{EPh}A^{EIn}C^{CA}U^{EPh}U^{EPh}C^{CA}U^{EPh}A^{EIn}A^{EIn}U^{EPh}C^{CA}U^{EPh}G^{PA}U^{EPh}A^{EIn}A^{EIn}G^{PA}A^{EIn}G^{PA}C^{CA}A^{EIn}G^{PA}A^{EIn}U^{EPh}C^{CA}C^{CA}C^{CA}U^{EPh}G^{PA}G^{PA}A^{EIn}C^{CA}A^{EIn}G^{PA}G^{PA}C^{CA}A^{EIn}A^{EIn}G^{PA}G^{PA}A^{EIn}A^{EIn}U^{EPh}A^{EIn}C^{CA}A^{EIn}G^{PA}G^{PA}U^{EPh}A^{EIn}U^{EPh}U^{EPh}U^{EPh}U^{EPh}G^{PA}U^{EPh}C^{CA}C^{CA}U^{EPh}U^{EPh}G^{PA}}$
**98DNA_ds$\mathrm{C^{CA}G^{PA}U^{EPh}A^{EIn}}$** *c*	$\mathrm{CAAGGACAAAATACCTGTATTCCTTG^{PA}C^{CA}C^{CA}U^{EPh}G^{PA}U^{EPh}C^{CA}C^{CA}A^{EIn}G^{PA}G^{PA}G^{PA}A^{EIn}U^{EPh}C^{CA}U^{EPh}G^{PA}C^{CA}U^{EPh}C^{CA}U^{EPh}U^{EPh}A^{EIn}C^{CA}A^{EIn}G^{PA}A^{EIn}U^{EPh}U^{EPh}A^{EIn}G^{PA}A^{EIn}A^{EIn}G^{PA}U^{EPh}A^{EIn}G^{PA}U^{EPh}C^{CA}C^{CA}U^{EPh}A^{EIn}U^{EPh}U^{EPh}A^{EIn}G^{PA}C^{CA}C^{CA}C^{CA}A^{EIn}G^{PA}A^{EIn}G^{PA}G^{PA}C^{CA}G^{PA}A^{EIn}U^{EPh}G^{PA}U^{EPh}C^{CA}U^{EPh}C^{CA}U^{EPh}C^{CA}A^{EIn}U^{EPh}G^{PA}A^{EIn}U^{EPh}G^{PA}U^{EPh}C^{CA}}$
**120DNA_$\mathrm{C^{CA}G^{PA}U^{SA}A^{OP}}$** *b*	$\mathrm{CAAGGACAAAATACCTGTATTCCTTG^{PA}C^{CA}U^{SA}C^{CA}A^{OP}U^{SA}G^{PA}C^{CA}U^{SA}A^{OP}G^{PA}A^{OP}C^{CA}U^{SA}G^{PA}A^{OP}G^{PA}C^{CA}U^{SA}C^{CA}A^{OP}U^{SA}G^{PA}C^{CA}U^{SA}A^{OP}G^{PA}A^{OP}C^{CA}U^{SA}G^{PA}A^{OP}G^{PA}C^{CA}U^{SA}C^{CA}A^{OP}U^{SA}G^{PA}C^{CA}U^{SA}A^{OP}G^{PA}A^{OP}C^{CA}U^{SA}G^{PA}A^{OP}G^{PA}C^{CA}U^{SA}C^{CA}A^{OP}U^{SA}G^{PA}C^{CA}U^{SA}A^{OP}G^{PA}A^{OP}C^{CA}U^{SA}G^{PA}A^{OP}G^{PA}C^{CA}U^{SA}C^{CA}A^{OP}U^{SA}G^{PA}C^{CA}U^{SA}A^{OP}G^{PA}G^{PA}C^{CA}G^{PA}A^{OP}U^{SA}G^{PA}U^{SA}C^{CA}U^{SA}C^{CA}U^{SA}C^{CA}A^{OP}U^{SA}G^{PA}A^{OP}U^{SA}G^{PA}U^{SA}C^{CA}}$
**120DNA_$\mathrm{C^{CA}G^{PA}U^{EPh}A^{EIn}}$** *b*	$\mathrm{CAAGGACAAAATACCTGTATTCCTTG^{PA}C^{CA}U^{EPh}C^{CA}A^{EIn}U^{EPh}G^{PA}C^{CA}U^{EPh}A^{EIn}G^{PA}A^{EIn}C^{CA}U^{EPh}G^{PA}A^{EIn}G^{PA}C^{CA}U^{EPh}C^{CA}A^{EIn}U^{EPh}G^{PA}C^{CA}U^{EPh}A^{EIn}G^{PA}A^{EIn}C^{CA}U^{EPh}G^{PA}A^{EIn}G^{PA}C^{CA}U^{EPh}C^{CA}A^{EIn}U^{EPh}G^{PA}C^{CA}U^{EPh}A^{EIn}G^{PA}A^{EIn}C^{CA}U^{EPh}G^{PA}A^{EIn}G^{PA}C^{CA}U^{EPh}C^{CA}A^{EIn}U^{EPh}G^{PA}C^{CA}U^{EPh}A^{EIn}G^{PA}A^{EIn}C^{CA}U^{EPh}G^{PA}A^{EIn}G^{PA}C^{CA}U^{EPh}C^{CA}A^{EIn}U^{EPh}G^{PA}C^{CA}U^{EPh}A^{EIn}G^{PA}G^{PA}C^{CA}G^{PA}A^{EIn}U^{EPh}G^{PA}U^{EPh}C^{CA}U^{EPh}C^{CA}U^{EPh}C^{CA}A^{EIn}U^{EPh}G^{PA}A^{EIn}U^{EPh}G^{PA}U^{EPh}C^{CA}}$
**150DNA_$\mathrm{C^{CA}G^{PA}U^{SA}A^{OP}}$** *b*	$\mathrm{CAAGGACAAAATACCTGTATTCCTTU^{SA}C^{CA}A^{OP}U^{SA}G^{PA}C^{CA}U^{SA}A^{OP}G^{PA}A^{OP}C^{CA}U^{SA}G^{PA}A^{OP}G^{PA}C^{CA}U^{SA}C^{CA}A^{OP}U^{SA}G^{PA}C^{CA}U^{SA}A^{OP}G^{PA}A^{OP}C^{CA}U^{SA}G^{PA}A^{OP}G^{PA}C^{CA}U^{SA}C^{CA}A^{OP}U^{SA}G^{PA}C^{CA}U^{SA}A^{OP}G^{PA}A^{OP}C^{CA}U^{SA}G^{PA}A^{OP}G^{PA}C^{CA}U^{SA}C^{CA}A^{OP}U^{SA}G^{PA}C^{CA}U^{SA}A^{OP}G^{PA}A^{OP}C^{CA}U^{SA}G^{PA}A^{OP}G^{PA}C^{CA}U^{SA}C^{CA}A^{OP}U^{SA}G^{PA}C^{CA}U^{SA}A^{OP}G^{PA}A^{OP}C^{CA}U^{SA}G^{PA}A^{OP}G^{PA}C^{CA}U^{SA}C^{CA}A^{OP}U^{SA}G^{PA}C^{CA}U^{SA}A^{OP}G^{PA}A^{OP}C^{CA}U^{SA}G^{PA}A^{OP}G^{PA}C^{CA}U^{SA}C^{CA}A^{OP}U^{SA}G^{PA}C^{CA}U^{SA}A^{OP}G^{PA}G^{PA}C^{CA}G^{PA}A^{OP}U^{SA}G^{PA}U^{SA}C^{CA}U^{SA}C^{CA}U^{SA}C^{CA}A^{OP}U^{SA}G^{PA}A^{OP}U^{SA}G^{PA}U^{SA}C^{CA}}$
**150DNA_$\mathrm{C^{CA}G^{PA}U^{EPh}A^{EIn}}$** *b*	$\mathrm{CAAGGACAAAATACCTGTATTCCTTU^{EPh}C^{CA}A^{EIn}U^{EPh}G^{PA}C^{CA}U^{EPh}A^{EIn}G^{PA}A^{EIn}C^{CA}U^{EPh}G^{PA}A^{EIn}G^{PA}C^{CA}U^{EPh}C^{CA}A^{EIn}U^{EPh}G^{PA}C^{CA}U^{EPh}A^{EIn}G^{PA}A^{EIn}C^{CA}U^{EPh}G^{PA}A^{EIn}G^{PA}C^{CA}U^{EPh}C^{CA}A^{EIn}U^{EPh}G^{PA}C^{CA}U^{EPh}A^{EIn}G^{PA}A^{EIn}C^{CA}U^{EPh}G^{PA}A^{EIn}G^{PA}C^{CA}U^{EPh}C^{CA}A^{EIn}U^{EPh}G^{PA}C^{CA}U^{EPh}A^{EIn}G^{PA}A^{EIn}C^{CA}U^{EPh}G^{PA}A^{EIn}G^{PA}C^{CA}U^{EPh}C^{CA}A^{EIn}U^{EPh}}$

	G^{PA}C^{CA}U^{EPh}A^{EIn}G^{PA}A^{EIn}C^{CA}U^{EPh}G^{PA}A^{EIn}G^{PA}C^{CA}U^{EPh}C^{CA}A^{EIn}U^{EPh}G^{PA}C^{CA}U^{EPh}A^{EIn}G^{PA}A^{EIn}C^{CA}U^{EPh}G^{PA}A^{EIn}G^{PA}C^{CA}U^{EPh}C^{CA}A^{EIn}U^{EPh}G^{PA}C^{CA}U^{EPh}A^{EIn}G^{PA}G^{PA}C^{CA}G^{PA}A^{EIn}U^{EPh}G^{PA}U^{EPh}C^{CA}U^{EPh}C^{CA}U^{EPh}C^{CA}A^{EIn}U^{EPh}G^{PA}A^{EIn}U^{EPh}G^{PA}U^{EPh}C^{CA}
**118ON_C^{CA}G^{PA}U^{SA}A^{OP}** [b]	CATTCGGCTGCTCTTGATTTCAAGGACAAAATACCTGTATTCCTTG^{PA}C^{CA}C^{CA}U^{SA}G^{PA}U^{SA}C^{CA}C^{CA}A^{OP}G^{PA}G^{PA}G^{PA}A^{OP}U^{SA}C^{CA}U^{SA}G^{PA}C^{CA}U^{SA}C^{CA}U^{SA}U^{SA}A^{OP}C^{CA}A^{OP}G^{PA}A^{OP}U^{SA}U^{SA}A^{OP}G^{PA}A^{OP}A^{OP}G^{PA}U^{SA}A^{OP}G^{PA}U^{SA}C^{CA}C^{CA}U^{SA}A^{OP}U^{SA}U^{SA}A^{OP}G^{PA}C^{CA}C^{CA}C^{CA}A^{OP}G^{PA}A^{OP}G^{PA}G^{PA}C^{CA}G^{PA}A^{OP}U^{SA}G^{PA}U^{SA}C^{CA}U^{SA}C^{CA}U^{SA}C^{CA}A^{OP}U^{SA}G^{PA}A^{OP}U^{SA}G^{PA}U^{SA}C^{CA}
**118ON_C^{CA}G^{PA}U^{EPh}A^{EIn}** [b]	CATTCGGCTGCTCTTGATTTCAAGGACAAAATACCTGTATTCCTTG^{PA}C^{CA}C^{CA}U^{EPh}G^{PA}U^{EPh}C^{CA}C^{CA}A^{EIn}G^{PA}G^{PA}G^{PA}A^{EIn}U^{EPh}C^{CA}U^{EPh}G^{PA}C^{CA}U^{EPh}C^{CA}U^{EPh}U^{EPh}A^{EIn}C^{CA}A^{EIn}G^{PA}A^{EIn}U^{EPh}U^{EPh}A^{EIn}G^{PA}A^{EIn}A^{EIn}G^{PA}U^{EPh}A^{EIn}G^{PA}U^{EPh}C^{CA}C^{CA}U^{EPh}A^{EIn}U^{EPh}U^{EPh}A^{EIn}G^{PA}C^{CA}C^{CA}C^{CA}A^{EIn}G^{PA}A^{EIn}G^{PA}G^{PA}C^{CA}G^{PA}A^{EIn}U^{EPh}G^{PA}U^{EPh}C^{CA}U^{EPh}C^{CA}U^{EPh}C^{CA}A^{EIn}U^{EPh}G^{PA}A^{EIn}U^{EPh}G^{PA}U^{EPh}C^{CA}
**98PCR_G^{PA}** [c]	CAAGGACAAAATACCTGTATTCCTTG^{PA}CCTG^{PA}TCCAG^{PA}G^{PA}G^{PA}ATCTG^{PA}CTCTTACAG^{PA}ATTAG^{PA}AAG^{PA}TAG^{PA}TCCTATTAG^{PA}CCCAG^{PA}AG^{PA}G^{PA}CG^{PA}ATG^{PA}TCTCTCATG^{PA}ATG^{PA}TC
**98PCR_A^{OP}** [c]	CAAGGACAAAATACCTGTATTCCTTGCCTGTCCA^{OP}GGGA^{OP}TCTGCTCTTA^{OP}CA^{OP}GA^{OP}TTA^{OP}GA^{OP}A^{OP}GTA^{OP}GTCCTA^{OP}TTA^{OP}GCCCA^{OP}GA^{OP}GGCGA^{OP}TGTCTCTCA^{OP}TGA^{OP}TGTC
**98PCR_U^{SA}** [c]	CAAGGACAAAATACCTGTATTCCTTGCCU^{SA}GU^{SA}CCAGGGAU^{SA}CU^{SA}GCU^{SA}CU^{SA}U^{SA}ACAGAU^{SA}U^{SA}AGAAGU^{SA}AGU^{SA}CCU^{SA}AU^{SA}U^{SA}AGCCCAGAGGCGAU^{SA}GU^{SA}CU^{SA}CU^{SA}CAU^{SA}GAU^{SA}GU^{SA}C
**98PCR_C^{CA}** [c]	CAAGGACAAAATACCTGTATTCCTTGC^{CA}C^{CA}TGTC^{CA}C^{CA}AGGGATC^{CA}TGC^{CA}TC^{CA}TTAC^{CA}AGATTAGAAGTAGTC^{CA}C^{CA}TATTAGC^{CA}C^{CA}C^{CA}AGAGGC^{CA}GATGTC^{CA}TC^{CA}TC^{CA}ATGATGTC^{CA}
**98PCR_A^{OP}C^{CA}** [c]	CAAGGACAAAATACCTGTATTCCTTGC^{CA}C^{CA}TGTC^{CA}C^{CA}A^{OP}GGGA^{OP}TC^{CA}TGC^{CA}TC^{CA}TTA^{OP}C^{CA}A^{OP}GA^{OP}TTA^{OP}GA^{OP}A^{OP}GTA^{OP}GTC^{CA}C^{CA}TA^{OP}TTA^{OP}GC^{CA}C^{CA}C^{CA}A^{OP}GA^{OP}GGC^{CA}GA^{OP}TGTC^{CA}TC^{CA}TC^{CA}A^{OP}TGA^{OP}TGTC^{CA}
**98PCR_G^{PA}C^{CA}** [c]	CAAGGACAAAATACCTGTATTCCTTG^{PA}C^{CA}C^{CA}TG^{PA}TC^{CA}C^{CA}AG^{PA}G^{PA}G^{PA}ATC^{CA}TG^{PA}C^{CA}TC^{CA}TTAC^{CA}AG^{PA}ATTAG^{PA}AAG^{PA}TAG^{PA}TC^{CA}C^{CA}TATTAG^{PA}C^{CA}C^{CA}C^{CA}AG^{PA}AG^{PA}G^{PA}C^{CA}G^{PA}ATG^{PA}TC^{CA}TC^{CA}TC^{CA}ATG^{PA}ATG^{PA}TC^{CA}
**98PCR_U^{SA}C^{CA}** [c]	CAAGGACAAAATACCTGTATTCCTTGC^{CA}C^{CA}U^{SA}GU^{SA}C^{CA}C^{CA}AGGGAU^{SA}C^{CA}U^{SA}GC^{CA}U^{SA}C^{CA}U^{SA}U^{SA}AC^{CA}AGAU^{SA}U^{SA}AGAAGU^{SA}AGU^{SA}C^{CA}C^{CA}U^{SA}AU^{SA}U^{SA}AGC^{CA}C^{CA}C^{CA}AGAGGC^{CA}GAU^{SA}GU^{SA}C^{CA}U^{SA}C^{CA}U^{SA}C^{CA}AU^{SA}GAU^{SA}GU^{SA}C^{CA}
**98PCR_A^{OP}G^{PA}** [c]	CAAGGACAAAATACCTGTATTCCTTG^{PA}CCTG^{PA}TCCA^{OP}G^{PA}G^{PA}G^{PA}A^{OP}TCTG^{PA}CTCTTA^{OP}CA^{OP}G^{PA}A^{OP}TTA^{OP}G^{PA}A^{OP}A^{O}

	^{P}G^{PA}TA^{OP}G^{PA}TCCTA^{OP}TTA^{OP}G^{PA}CCCA^{OP}G^{PA}A^{OP}G^{PA}G^{PA}CG^{PA}A^{OP}TG^{PA}TCTCTCA^{OP}TG^{PA}A^{OP}TG^{PA}TC
**98PCR_U^{SA}A^{OP}** ^{c}	CAAGGACAAAATACCTGTATTCCTTGCCU^{SA}GU^{SA}CCA^{OP}GGGA^{OP}U^{SA}CU^{SA}GCU^{SA}CU^{SA}U^{SA}A^{OP}CA^{OP}GA^{OP}U^{SA}U^{SA}A^{OP}GA^{OP}A^{OP}GU^{SA}A^{OP}GU^{SA}CCU^{SA}A^{OP}U^{SA}U^{SA}A^{OP}GCCCA^{OP}GA^{OP}GGCGA^{OP}U^{SA}GU^{SA}CU^{SA}CU^{SA}CA^{OP}U^{SA}GA^{OP}U^{SA}GU^{SA}C
**98PCR_G^{PA}U^{SA}** ^{c}	CAAGGACAAAATACCTGTATTCCTTG^{PA}CCU^{SA}G^{PA}U^{SA}CCAG^{PA}G^{PA}G^{PA}AU^{SA}CU^{SA}G^{PA}CU^{SA}CU^{SA}U^{SA}ACAG^{PA}AU^{SA}U^{SA}AG^{PA}AAG^{PA}U^{SA}AG^{PA}U^{SA}CCU^{SA}AU^{SA}U^{SA}AG^{PA}CCCAG^{PA}AG^{PA}G^{PA}CG^{PA}AU^{SA}G^{PA}U^{SA}CU^{SA}CU^{SA}CAU^{SA}G^{PA}AU^{SA}G^{PA}U^{SA}C
**98PCR_C^{CA}G^{PA}A^{OP}** ^{c}	CAAGGACAAAATACCTGTATTCCTTG^{PA}C^{CA}C^{CA}TG^{PA}TC^{CA}C^{CA}A^{OP}G^{PA}G^{PA}G^{PA}A^{OP}TC^{CA}TG^{PA}C^{CA}TC^{CA}TTA^{OP}C^{CA}A^{OP}G^{PA}A^{OP}TTA^{OP}G^{PA}A^{OP}A^{OP}G^{PA}TA^{OP}G^{PA}TC^{CA}C^{CA}TA^{OP}TTA^{OP}G^{PA}C^{CA}C^{CA}C^{CA}A^{OP}G^{PA}A^{OP}G^{PA}G^{PA}C^{CA}G^{PA}A^{OP}TG^{PA}TC^{CA}TC^{CA}TC^{CA}A^{OP}TG^{PA}A^{OP}TG^{PA}TC^{CA}
**98ON_A^{OP}U^{SA}C^{CA}** ^{b}	CAAGGACAAAATACCTGTATTCCTTGC^{CA}C^{CA}U^{SA}GU^{SA}C^{CA}C^{CA}A^{OP}GGGA^{OP}U^{SA}C^{CA}U^{SA}GC^{CA}U^{SA}C^{CA}U^{SA}U^{SA}A^{OP}C^{CA}A^{OP}GA^{OP}U^{SA}U^{SA}A^{OP}GA^{OP}A^{OP}GU^{SA}A^{OP}GU^{SA}C^{CA}C^{CA}U^{SA}A^{OP}U^{SA}U^{SA}A^{OP}GC^{CA}C^{CA}C^{CA}A^{OP}GA^{OP}GGC^{CA}GA^{OP}U^{SA}GU^{SA}C^{CA}U^{SA}C^{CA}U^{SA}C^{CA}A^{OP}U^{SA}GA^{OP}U^{SA}GU^{SA}C^{CA}
**98ON_A^{OP}U^{SA}G^{PA}** ^{b}	CAAGGACAAAATACCTGTATTCCTTGCCU^{SA}G^{PA}U^{SA}CCA^{OP}G^{PA}G^{PA}G^{PA}A^{OP}U^{SA}CU^{SA}G^{PA}CU^{SA}CU^{SA}U^{SA}A^{OP}CA^{OP}G^{PA}A^{OP}U^{SA}U^{SA}A^{OP}G^{PA}A^{OP}A^{OP}G^{PA}U^{SA}A^{OP}G^{PA}U^{SA}CCU^{SA}A^{OP}U^{SA}U^{SA}A^{OP}G^{PA}CCCA^{OP}G^{PA}A^{OP}G^{PA}G^{PA}CG^{PA}A^{OP}U^{SA}G^{PA}U^{SA}CU^{SA}CU^{SA}CA^{OP}U^{SA}G^{PA}A^{OP}U^{SA}G^{PA}U^{SA}C
**98ON_C^{CA}G^{PA}U^{SA}** ^{b}	CAAGGACAAAATACCTGTATTCCTTG^{PA}C^{CA}C^{CA}U^{SA}G^{PA}U^{SA}C^{CA}C^{CA}AG^{PA}G^{PA}G^{PA}AU^{SA}C^{CA}U^{SA}G^{PA}C^{CA}U^{SA}C^{CA}U^{SA}U^{SA}AC^{CA}AG^{PA}AU^{SA}U^{SA}AG^{PA}AAG^{PA}U^{SA}AG^{PA}U^{SA}C^{CA}C^{CA}U^{SA}AU^{SA}U^{SA}AG^{PA}C^{CA}C^{CA}C^{CA}AG^{PA}AG^{PA}G^{PA}C^{CA}G^{PA}AU^{SA}G^{PA}U^{SA}C^{CA}U^{SA}C^{CA}U^{SA}C^{CA}AU^{SA}G^{PA}AU^{SA}G^{PA}U^{SA}C^{CA}
**98ON_ C^{CA}G^{PA}U^{EPh}A^{EIn}** ^{b}	CAAGGACAAAATACCTGTATTCCTTG^{PA}C^{CA}C^{CA}U^{EPh}G^{PA}U^{EPh}C^{CA}C^{CA}A^{EIn}G^{PA}G^{PA}G^{PA}A^{EIn}U^{EPh}C^{CA}U^{EPh}G^{PA}C^{CA}U^{EPh}C^{CA}U^{EPh}U^{EPh}A^{EIn}C^{CA}A^{EIn}G^{PA}A^{EIn}U^{EPh}U^{EPh}A^{EIn}G^{PA}A^{EIn}A^{EIn}G^{PA}U^{EPh}A^{EIn}G^{PA}U^{EPh}C^{CA}C^{CA}U^{EPh}A^{EIn}U^{EPh}U^{EPh}A^{EIn}G^{PA}C^{CA}C^{CA}C^{CA}A^{EIn}G^{PA}A^{EIn}G^{PA}G^{PA}C^{CA}G^{PA}A^{EIn}U^{EPh}G^{PA}U^{EPh}C^{CA}U^{EPh}C^{CA}U^{EPh}C^{CA}A^{EIn}U^{EPh}G^{PA}A^{EIn}U^{EPh}G^{PA}U^{EPh}C^{CA}
**98PCR_ C^{CA}G^{PA}U^{EPh}A^{EIn}** ^{c}	CAAGGACAAAATACCTGTATTCCTTG^{PA}C^{CA}C^{CA}U^{EPh}G^{PA}U^{EPh}C^{CA}C^{CA}A^{EIn}G^{PA}G^{PA}G^{PA}A^{EIn}U^{EPh}C^{CA}U^{EPh}G^{PA}C^{CA}U^{EPh}C^{CA}U^{EPh}U^{EPh}A^{EIn}C^{CA}A^{EIn}G^{PA}A^{EIn}U^{EPh}U^{EPh}A^{EIn}G^{PA}A^{EIn}A^{EIn}G^{PA}U^{EPh}A^{EIn}G^{PA}U^{EPh}C^{CA}C^{CA}U^{EPh}A^{EIn}U^{EPh}U^{EPh}A^{EIn}G^{PA}C^{CA}C^{CA}C^{CA}A^{EIn}G^{PA}A^{EIn}G^{PA}G^{PA}C^{CA}G^{PA}A^{EIn}U^{EPh}G^{PA}U^{EPh}C^{CA}U^{EPh}C^{CA}U^{EPh}C^{CA}A^{EIn}U^{EPh}G^{PA}A^{EIn}U^{EPh}G^{PA}U^{EPh}C^{CA}

Note: ON – single-stranded DNA; DNA – double-stranded DNA (a complementary strand is an unmodified template); PCR – a double-stranded PCR product

[a] primer region underlined; [b] 5'-(6-FAM)-labelled extended strand after PEX or PCR; [c] a complementary strand is modified in the same manner using modification under study (primer regions are not modified)

2.1. PEX – Single incorporation (one modified dN^{R}TP)

The reaction mixture (10 µL) contained one of 19-mer templates Temp^{Oligo1A} / Temp^{Oligo1T} / Temp^{Oligo1C} / Temp^{Oligo1G} (3 µM, 0.75 µL), primer Prim^{248short}-FAM (3 µM, 0.5 µL), dGTP (dTTP in the case of Temp^{Oligo1G}) (0.25 mM, 1 µL), modified **dN^{R}TP** of study (R = SA, OP, PA, CA; N = U, A, G, C) (2 mM, 1 µL each), either KOD XL (0.1 U) or Vent(exo-) DNA polymerase (units specification in Table S3) and a corresponding reaction buffer (10X, 1 µL) as supplied by the manufacturer. The reaction mixture was incubated for 30 min at 60 °C, stopped by addition of PAGE stop solution (10 µL) and denatured for 3 min at 95 °C. Samples were analyzed by PAGE and visualized using fluorescence imaging (Figure S1).

Table S3. Reaction condition specifications for single incorporation.

Template	Vent(exo-)	dN^{R}TP
Temp^{Oligo1T}	0.1 U	**dU^{SA}TP**
Temp^{Oligo1A}	0.1 U	**dA^{OP}TP**
Temp^{Oligo1G}	0.2 U	**dG^{PA}TP**
Temp^{Oligo1C}	0.2 U	**dC^{CA}TP**

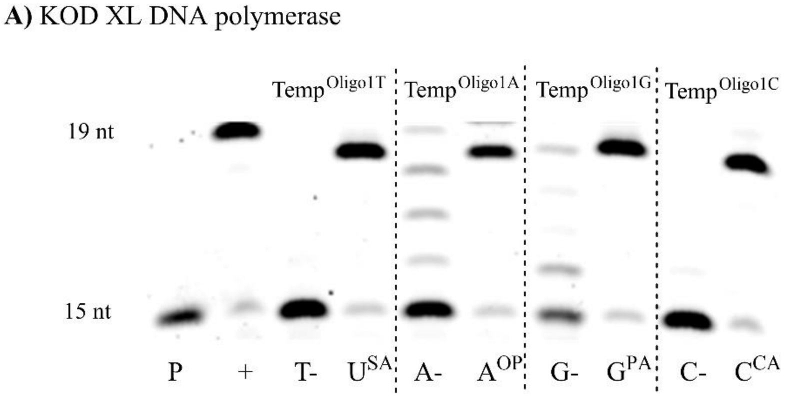

B) Vent(exo-) DNA polymerase

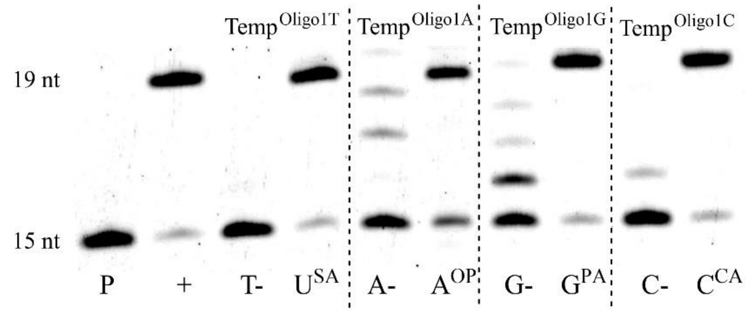

Figure S1. Denaturing PAGE analysis of PEX reaction with one modified **dN^{R}TP** using: A) KOD XL DNA polymerase, B) Vent(exo-) DNA polymerase: (P) primer Prim^{248short}-FAM; template Temp^{Oligo1T}: (+) dTTP, dGTP; (T-) dGTP; (U^{SA}) **dU^{SA}TP**, dGTP; template Temp^{Oligo1A}: (A-) dGTP; (A^{OP}) **dA^{OP}TP**, dGTP; template Temp^{Oligo1G}: (G-) dTTP; (G^{PA}) **dG^{PA}TP**, dTTP; template Temp^{Oligo1C}: (C-) dGTP; (C^{CA}) **dC^{CA}TP**, dGTP.

2.2. PEX – Multiple incorporation (one modified dN^{R}TP)

The reaction mixture (10 µL) contained 31-mer template Temp^{Prb4basII} (3 µM, 0.75 µL), primer Prim^{248short}-FAM (3 µM, 0.5 µL), appropriate natural dNTPs (0.25 mM, 1 µL each), modified **dN^{R}TP** of study (R = SA, OP, PA, CA; N = U, A, G, C) (2 mM, 1 µL each), either KOD XL (0.3 U) or Vent(exo-) DNA polymerase (units specified in Table S4) and a corresponding reaction buffer (10X, 1 µL) as supplied by the manufacturer. The positive control contained 0.5 U of Vent(exo-) DNA polymerase and natural dNTPs (1 mM, 1 µL). The reaction mixture was incubated for 30 min at 60 °C, stopped by addition of PAGE stop solution (10 µL) and denatured for 3 min at 95 °C. Samples were analyzed by PAGE and visualized using fluorescence imaging (Figure S2).

Table S4. Reaction condition specifications for multiple incorporation.

**dN^{R}TP**s	Vent(exo-)
**dC^{CA}TP**	1 U
**dG^{PA}TP**	0.4 U
**dA^{OP}TP**	0.5 U
**dU^{SA}TP**	1.2 U

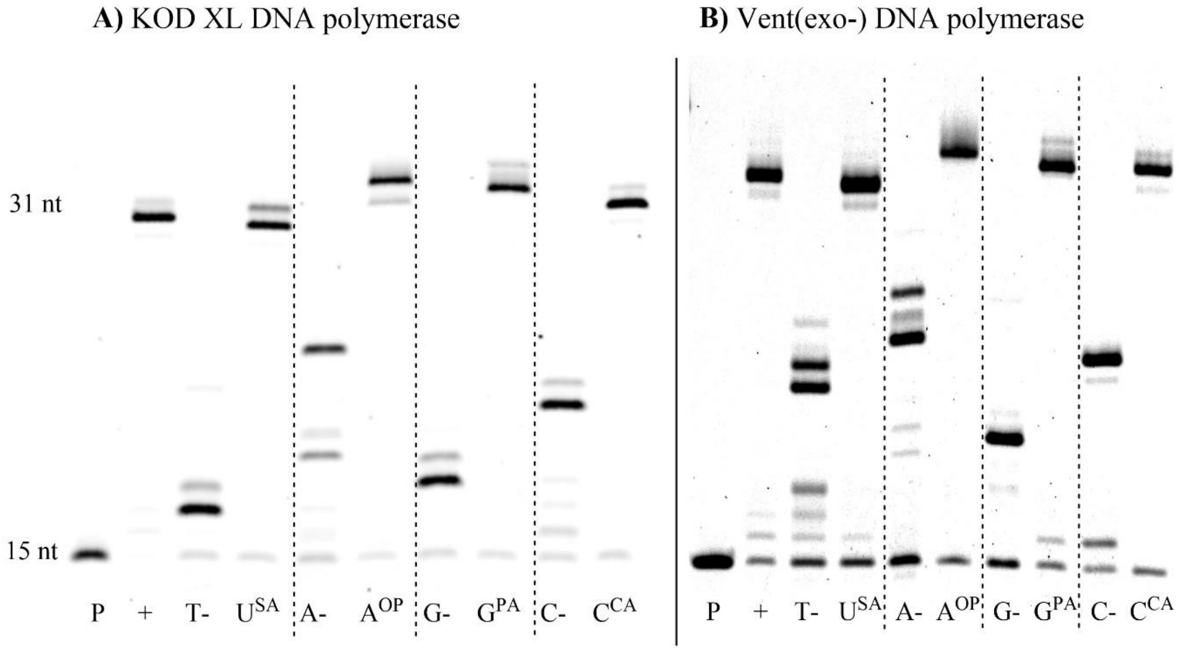

Figure S2. Denaturing PAGE analysis of PEX reaction with one modified **dN^{R}TP** using: A) KOD XL DNA polymerase, B) Vent(exo-) DNA polymerase: (P) primer Prim^{248short}-FAM; template Temp^{Prb4basII}: (+) natural dNTPs; (T-) dCTP, dATP, dGTP; (U^{SA}) dCTP, dATP, dGTP, **dU^{SA}TP**; (A-) dCTP, dGTP, dTTP; (A^{OP}) dCTP, **dA^{OP}TP**, dGTP, dTTP; (G-) dCTP, dATP, dTTP; (G^{PA}) dCTP, dATP, **dG^{PA}TP**, dTTP; (C-) dATP, dGTP, dTTP; (C^{CA}) **dC^{CA}TP**, dATP, dGTP, dTTP.

2.3. PEX – Multiple incorporation (two, three and four modified dN^{R}TPs)

The reaction mixture (10 µL) contained 31-mer template Temp^{Prb4basII} (3 µM, 0.75 µL), primer Prim^{248short}-FAM (3 µM, 0.5 µL), appropriate natural dNTPs (0.25 mM, 1 µL) or none in case of four modifications, modified **dN^{R}TP**s of study (R = SA, OP, PA, CA; N = U, A, G, C) (1 µL, see specifications in Table S5), Vent(exo-) DNA polymerase (Table S5), and the enzyme reaction buffer (10X, 1 µL) as supplied by the manufacturer. The reaction mixture was incubated for 30 min at 60 °C, stopped by addition of PAGE stop solution (10 µL) and denatured for 3 min at 95 °C. Samples were analyzed by PAGE and visualized using fluorescence imaging (Figure S3).

Table S5. Reaction condition specifications for multiple incorporation of combinations of **dN^{R}TP**s.

Lanes in Figure S3	Natural dNTPs	Vent(exo-)	**dC^{CA}TP**	**dG^{PA}TP**	**dA^{OP}TP**	**dU^{SA}TP**
4	dGTP, dTTP	0.5 U	0.25 mM	–	2 mM	–
7	dATP, dTTP	0.5 U	2 mM	2 mM	–	–
10	dATP, dGTP	0.5 U	2 mM	–	–	2 mM
13	dCTP, dTTP	0.5 U	–	2 mM	2 mM	–
16	dCTP, dGTP	1.5 U	–	–	2 mM	2 mM
19	dCTP, dATP	0.5 U	–	2 mM	–	2 mM
23	dTTP	0.5 U	0.25 mM	2 mM	2 mM	–
27	dGTP	0.5 U	0.25 mM	–	2 mM	2 mM
31	dATP	0.5 U	2 mM	2 mM	–	2 mM
35	dCTP	0.5 U	–	2 mM	2 mM	2 mM
40	–	1.0 U	0.25 mM	2 mM	2 mM	2 mM

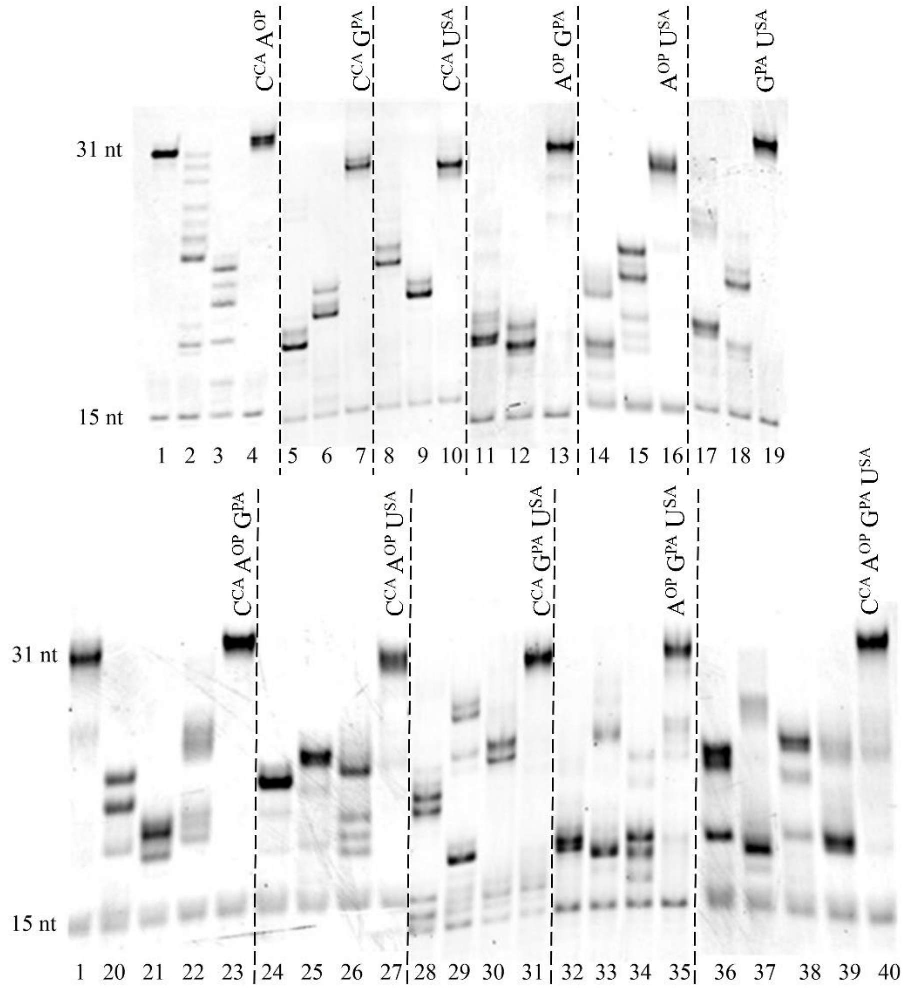

Figure S3. Denaturing PAGE analysis of PEX reaction with combinations of modified **dN^{R}TP**s using Vent(exo-) DNA polymerase, primer Prim^{248short}-FAM, and template Temp^{Prb4basII}: (lanes 1) natural dNTPs; (lane 2) **dC^{CA}TP**, dGTP, dTTP; (lane 3) **dA^{OP}TP**, dGTP, dTTP; (lane 4) **dC^{CA}TP**, **dA^{OP}TP**, dGTP, dTTP; (lane 5) **dC^{CA}TP**, dATP, dTTP; (lane 6) dATP, **dG^{PA}TP**, dTTP; (lane 7) **dC^{CA}TP**, dATP, **dG^{PA}TP**, dTTP; (lane 8) **dC^{CA}TP**, dATP, dGTP; (lane 9) dATP, dGTP, **dU^{SA}TP**; (lane 10) **dC^{CA}TP**, dATP, dGTP, **dU^{SA}TP**; (lane 11) dCTP, **dG^{PA}TP**, dTTP; (lane 12) dCTP, **dA^{OP}TP**, dTTP; (lane 13) dCTP, **dA^{OP}TP**, **dG^{PA}TP**, dTTP; (lane 14) dCTP, **dA^{OP}TP**, dGTP; (lane 15) dCTP, dGTP, **dU^{SA}TP**; (lane 16) dCTP, **dA^{OP}TP**, dGTP, **dU^{SA}TP**; (lane 17) dCTP, dATP, **dU^{SA}TP**; (lane 18) dCTP, dATP, **dG^{PA}TP**; (lane 19) dCTP, dATP, **dG^{PA}TP**, **dU^{SA}TP**; (lane 20) **dA^{OP}TP**, **dG^{PA}TP**, dTTP; (lane 21) **dC^{CA}TP**, **dA^{OP}TP**, dTTP; (lane 22) **dC^{CA}TP**, **dG^{PA}TP**, dTTP; (lane 23) **dC^{CA}TP**, **dA^{OP}TP**, **dG^{PA}TP**, dTTP; (lane 24) **dA^{OP}TP**, dGTP, **dU^{SA}TP**; (lane 25) **dC^{CA}TP**, **dA^{OP}TP**,

dGTP; (lane 26) **dC^{CA}TP**, dGTP, **dU^{SA}TP**; (lane 27) **dC^{CA}TP**, **dA^{OP}TP**, dGTP, **dU^{SA}TP**; (lane 28) dATP, **dG^{PA}TP**, **dU^{SA}TP**; (lane 29) **dC^{CA}TP**, dATP, **dU^{SA}TP**; (lane 30) **dC^{CA}TP**, dATP, **dG^{PA}TP**; (lane 31) **dC^{CA}TP**, dATP, **dG^{PA}TP**, **dU^{SA}TP**; (lane 32) dCTP, **dG^{PA}TP**, **dU^{SA}TP**; (lane 33) dCTP, **dA^{OP}TP**, **dU^{SA}TP**; (lane 34) dCTP, **dA^{OP}TP**, **dG^{PA}TP**; (lane 35) dCTP, **dA^{OP}TP**, **dG^{PA}TP**, **dU^{SA}TP**; (lane 36) **dA^{OP}TP**, **dG^{PA}TP**, **dU^{SA}TP**; (lane 37) **dC^{CA}TP**, **dA^{OP}TP**, **dU^{SA}TP**; (lane 38) **dC^{CA}TP**, **dA^{OP}TP**, **dG^{PA}TP**; (lane 39) **dC^{CA}TP**, **dG^{PA}TP**, **dU^{SA}TP**; (lane 40) **dC^{CA}TP**, **dA^{OP}TP**, **dG^{PA}TP**, **dU^{SA}TP**.

2.4. PEX – Multiple incorporation (dC^{CA}TP, dG^{PA}TP, dU^{SA}TP, dA^{OP}TP in various template length)

Method B. The reaction mixture (10 µL) contained one of two templates (Temp^{MO43}/ Temp^{MO61}) (3 µM, 0.75 µL), primer Prim^{248short}-FAM (3 µM, 0.5 µL), **dC^{CA}TP** (0.5 mM, 1 µL), **dG^{PA}TP** (2 mM, 1 µL), **dU^{SA}TP** (2 mM, 1 µL), **dA^{OP}TP** (4 mM, 1 µL), Vent(exo-) DNA polymerase (1 U), and the enzyme reaction buffer (10X, 1 µL) as supplied by the manufacturer. Positive controls contained 1 U of Vent(exo-) DNA polymerase and natural dNTPs (1 mM, 1 µL). The reaction mixture was incubated for 40 min at 60 °C, stopped by addition of PAGE stop solution (10 µL) and denatured for 5 min at 95 °C. Samples were analyzed by PAGE and visualized using fluorescence imaging (Figure S4 – lanes 1-4).

Method C. The reaction mixture (10 µL) contained one of three templates (Temp$^{FVL\text{-}A}$/Temp^{MO120}/Temp^{MO150}) (3 µM, 0.75 µL), primer Prim^{LT25TH}-FAM (3 µM, 0.5 µL), **dC^{CA}TP** (1 mM, 1 µL), **dG^{PA}TP** (2 mM, 1 µL), **dU^{SA}TP** (2 mM, 1 µL), **dA^{OP}TP** (4 mM, 1 µL), Vent(exo-) DNA polymerase (1 U), and the enzyme reaction buffer (10X, 1 µL) as supplied by the manufacturer. Positive controls contained 0.5 U of Vent(exo-) DNA polymerase and natural dNTPs (1 mM, 1 µL). The reaction mixture was incubated for 40 min at 60 °C, stopped by addition of PAGE stop solution (10 µL) and denatured for 5 min at 95 °C. Samples were analyzed by PAGE and visualized using fluorescence imaging (Figure S4 – lanes 5-10).

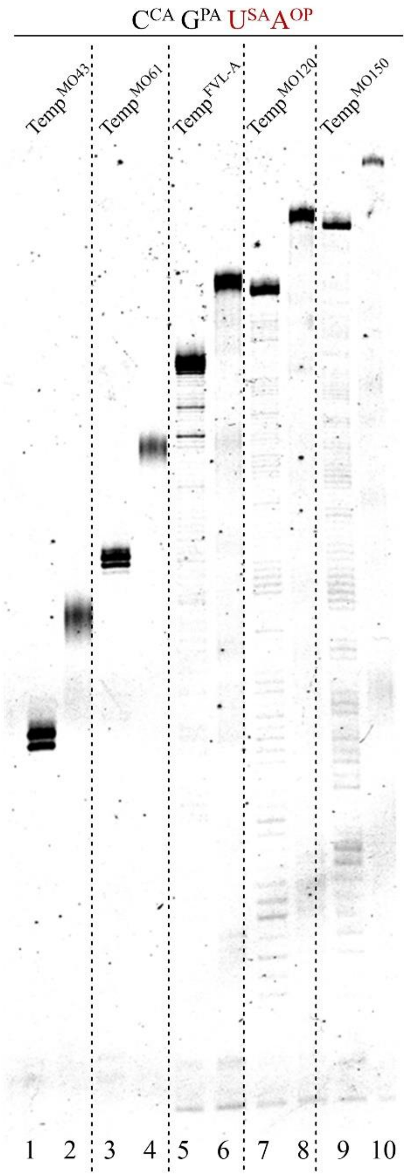

Figure S4. Denaturing PAGE analysis of PEX reactions with the set of four modified **dN^{R}TP**s (R = SA, OP, PA, CA; N = U, A, G, C) using Vent(exo-) DNA polymerase and various templates. For PEX with primer Prim^{248short}-FAM: template Temp^{MO43}: (lane 1) natural dNTPs; (lane 2) **dC^{CA}TP**, **dA^{OP}TP**, **dG^{PA}TP**, **dU^{SA}TP**; template Temp^{MO61}: (lane 3) natural dNTPs; (lane 4) **dC^{CA}TP**, **dA^{OP}TP**, **dG^{PA}TP**, **dU^{SA}TP**. For PEX with primer Prim^{LT25TH}-FAM: template Temp^{FVL-A}: (lane 5) natural dNTPs; (lane 6) **dC^{CA}TP**, **dA^{OP}TP**, **dG^{PA}TP**, **dU^{SA}TP**; template Temp^{MO120}: (lane 7) natural dNTPs; (lane 8) **dC^{CA}TP**, **dA^{OP}TP**, **dG^{PA}TP**, **dU^{SA}TP**; template Temp^{MO150}: (lane 9) natural dNTPs; (lane 10) **dC^{CA}TP**, **dA^{OP}TP**, **dG^{PA}TP**, **dU^{SA}TP**.

2.5. PEX – Multiple incorporation (dC^{CA}TP, dG^{PA}TP, dU^{EPh}TP, dA^{EIn}TP in various template length)

Method D. The reaction mixture (10 µL) contained one of three templates (Temp^{Prb4basII}/Temp^{MO43}/ Temp^{MO61}) (3 µM, 0.75 µL), primer Prim^{248short}-FAM (3 µM, 0.5 µL), **dC^{CA}TP** (0.1 mM, 0.5 µL for Temp^{Prb4basII}; 0.5 mM, 0.5 µL for Temp^{MO43} and Temp^{MO61}), **dG^{PA}TP** (2 mM, 1 µL), **dU^{EPh}TP** (2 mM, 1 µL), **dA^{EIn}TP** (2 mM, 1 µL), KOD XL DNA polymerase (0.25 U), and the enzyme reaction buffer (10X, 1 µL) as supplied by the manufacturer. Positive controls contained 0.3 U of KOD XL DNA polymerase and natural dNTPs (1 mM, 1 µL). The reaction mixture was incubated for 40 min at 60 °C, stopped by addition of PAGE stop solution (10 µL) and denatured for 5 min at 95 °C. Samples were analyzed by PAGE and visualized using fluorescence imaging (Figure S5 – lanes 1-6).

Method E. The reaction mixture (10 µL) contained one of three templates (Temp^{FVL-A}/Temp^{MO120}/Temp^{MO150}) (3 µM, 0.75 µL), primer Prim^{LT25TH}-FAM (3 µM, 0.5 µL), **dC^{CA}TP** (1 mM, 0.5 µL), **dG^{PA}TP** (2 mM, 1 µL), **dU^{EPh}TP** (2 mM, 1 µL), **dA^{EIn}TP** (2 mM, 1 µL), KOD XL DNA polymerase (0.5 U), and the enzyme reaction buffer (10X, 1 µL) as supplied by the manufacturer. Positive controls contained 0.3 U of KOD XL DNA polymerase and natural dNTPs (1 mM, 1 µL). The reaction mixture was incubated for 40 min at 60 °C, stopped by addition of PAGE stop solution (10 µL) and denatured for 5 min at 95 °C. Samples were analyzed by PAGE and visualized using fluorescence imaging (Figure S5 – lanes 7-12).

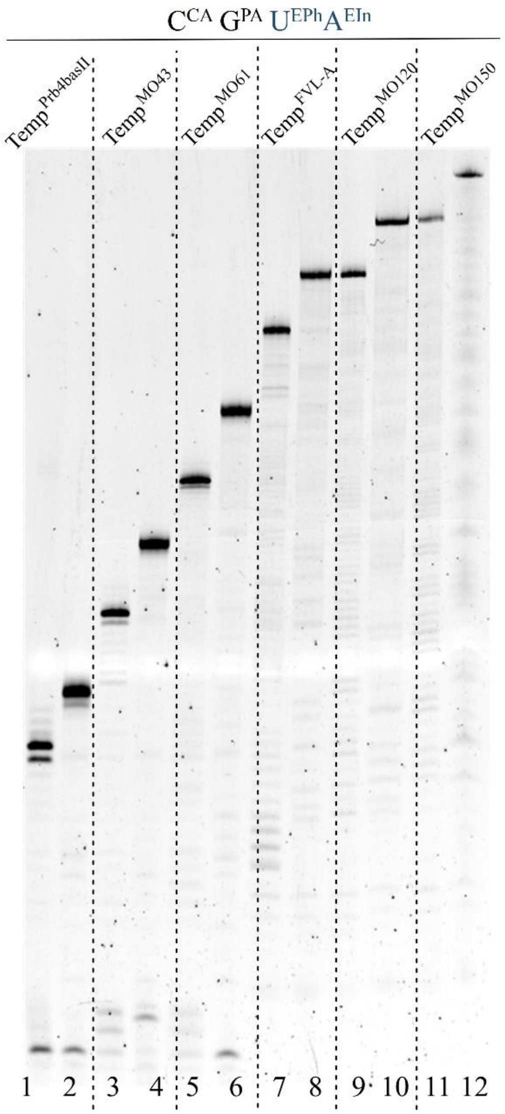

Figure S5. Denaturing PAGE analysis of PEX reactions with the set of four modified **dN^R^TP**s (R = EPh, EIn, PA, CA; N = U, A, G, C) using KOD XL DNA polymerase and various templates. For PEX with primer $\text{Prim}^{248\text{short}}$-FAM: template $\text{Temp}^{\text{Prb4basII}}$: (lane 1) natural dNTPs; (lane 2) $\mathbf{dC^{CA}TP}$, $\mathbf{dA^{EIn}TP}$, $\mathbf{dG^{PA}TP}$, $\mathbf{dU^{EPh}TP}$; template $\text{Temp}^{\text{MO43}}$: (lane 3) natural dNTPs; (lane 4) $\mathbf{dC^{CA}TP}$, $\mathbf{dA^{EIn}TP}$, $\mathbf{dG^{PA}TP}$, $\mathbf{dU^{EPh}TP}$; template $\text{Temp}^{\text{MO61}}$: (lane 5) natural dNTPs; (lane 6) $\mathbf{dC^{CA}TP}$, $\mathbf{dA^{EIn}TP}$, $\mathbf{dG^{PA}TP}$, $\mathbf{dU^{EPh}TP}$. For PEX with primer $\text{Prim}^{\text{LT25TH}}$-FAM: template $\text{Temp}^{\text{FVL-A}}$: (lane 7) natural dNTPs; (lane 8) $\mathbf{dC^{CA}TP}$, $\mathbf{dA^{EIn}TP}$, $\mathbf{dG^{PA}TP}$, $\mathbf{dU^{EPh}TP}$; template $\text{Temp}^{\text{MO120}}$: (lane 9) natural dNTPs; (lane 10) $\mathbf{dC^{CA}TP}$, $\mathbf{dA^{EIn}TP}$, $\mathbf{dG^{PA}TP}$, $\mathbf{dU^{EPh}TP}$; template $\text{Temp}^{\text{MO150}}$: (lane 11) natural dNTPs; (lane 12) $\mathbf{dC^{CA}TP}$, $\mathbf{dA^{EIn}TP}$, $\mathbf{dG^{PA}TP}$, $\mathbf{dU^{EPh}TP}$.

2.6. General procedure for ssDNA generation via magnetoseparation

Method G. In order to obtain sufficient amounts of **19ON_G^{PA}**, **19ON_A^{OP}**, **19ON_U^{SA}**, **19ON_C^{CA}** and **31ON_C^{CA}**, corresponding PEX reactions (described in sections 2.1, 2.2) containing primer Prim^{248short} and appropriate dual-biotinylated templates (for sequences see Table S1) were ten times scaled up. The reactions were stopped by cooling to 8 °C. Joined fractions were purified using the DBStv magnetoseparation procedure (described further in this section) followed by MALDI-TOF analysis (Table S6). For copies of MALDI-TOF spectra go to section 5, Figures S22-S26.

Method H. In order to obtain sufficient amounts of **31ON_A^{OP}**, **31ON_U^{SA}**, **31ON_G^{PA}**, **31ON_C^{CA}G^{PA}U^{SA}A^{OP}**, the reaction mixture (50 µL) contained template Temp^{Prb4basII}-bio (100 µM, 3.2 µL), primer Prim^{248short} (100 µM, 2.13 µL), appropriate natural dNTPs and modified **dN^{R}TP**s (4 mM, 5.2 µL each), Vent(exo-) DNA polymerase (3 U; only in the case of **dG^{PA}TP** 2.6 U), and the enzyme reaction buffer (10X, 5 µL) as supplied by the manufacturer and was incubated for 40 min at 60 °C. The reaction was stopped by cooling to 8 °C. Products were purified using the DBStv magnetoseparation procedure followed by MALDI-TOF analysis (Table S6). For copies of MALDI-TOF spectra go to section 5, Figures S27-S30.

Method I. In order to obtain sufficient amounts of **31ON_C^{CA}G^{PA}U^{EPh}A^{EIn}**, the reaction mixture (50 µL) containing template Temp^{Prb4basII}-bio (100 µM, 1.5 µL), primer Prim^{248short} (100 µM, 1.5 µL), modified **dN^{R}TP**s (4 mM, 4 µL each), KOD XL DNA polymerase (3 U), and the enzyme reaction buffer (10X, 5 µL) as supplied by the manufacturer was incubated for 40 min at 60 °C. The reaction was stopped by cooling to 8 °C. Product was purified using the DBStv magnetoseparation procedure followed by MALDI-TOF analysis (Table S6). For copies of MALDI-TOF spectra go to section 5, Figures S31.

DBStv magnetoseparation procedure. Streptavidin particles (50 µL) were washed with binding buffer (3 × 200 µL; 10 mM Tris, 1 mM EDTA, 100 mM NaCl, pH 7.5). 50 µL (Methods H, I) or 100 µL (Method G) of PEX solutions were mixed with binding buffer (200 µL) and incubated for 30 min at 15 °C and 1400 rpm. The magnetic beads were collected on a magnet (DynaMagTM-2, Invitrogen), and washed with washing buffer (3 × 300 µL; 10 mM Tris, 1 mM EDTA, 500 mM NaCl, pH 7.5) and water (4 × 300 µL). Then water (50 µL) was added and the

sample was denatured for 2 min at 900 rpm and 75 °C. The beads were collected on a magnet and the solution was transferred into a clean vial. The product was concentrated and analyzed by MALDI-TOF analysis (Table S6).

2.7. MALDI-TOF measurements

Table S6. Overview of modified ssONs and their masses after magnetoseparation.

ON name	Mass calculated [Da]	Mass found [Da]	Δ [Da]	Figure number
**19ON_C^{CA}**	6060.9	6062.1	1.2	S22
**19ON_G^{PA}**	6046.9	6047.8	0.9	S23
**19ON_A^{OP}**	6107.9	6108.3	0.4	S24
**19ON_U^{SA}**	6069.9	6071.0	1.1	S25
**31ON_C^{CA}**	10057.3	10058.4	1.1	S26
**31ON_G^{PA}**	10141.3	10142.4	1.1	S27
**31ON_A^{OP}**	10149.3	10150.5	1.2	S28
**31ON_U^{SA}**	10033.3	10034.2	0.9	S29
**31ON_C^{CA}G^{PA}U^{SA}A^{OP}**	11529.3	11533.0	3.7	S30
**31ON_C^{CA}G^{PA}U^{EPh}A^{EIn}**	11477.3	11478.5	1.2	S31

2.8. PCR – Multiple incorporation (one modified dN^{R}TP)

The reaction mixture (10 μL) contained template Temp$^{FVL\text{-}A}$ (0.5 μM, 0.5 μL), reverse primer Prim^{LT25TH}-FAM and forward primer Prim^{L20}-Cy5 (10 μM, 1 μL each), appropriate set of natural dNTPs (2 mM, 1 μL), one of the modified **dN^{R}TP**s (R = SA, OP, PA, CA; N = U, A, G, C) (1 μL, conditions specified in Table S7), either Vent(exo-) or KOD XL DNA polymerase (Table S7) and a corresponding reaction buffer (10X, 1 μL) as supplied by the manufacturer. The positive control contained either 1 U of Vent(exo-) or 0.5 U of KOD XL DNA polymerase and natural dNTPs (2 mM, 2 μL). All reaction mixtures were under cycling protocol: 94 °C for 3 min, followed by 30 cycles at 94 °C for 30 sec, 53 °C for 30 sec, and 72 °C for 1 min, followed by a final elongation step at 72 °C for 5 min. Samples were analyzed by PAGE and agarose gel electrophoresis and

visualized using fluorescence imaging (Figure S6A – PCR with KOD XL, Figure S6B – PCR with Vent(exo-)).

Table S7. Reaction condition specifications for PCR (one modified **dN^{R}TP**).

Entry	dN^{R}TP, mM	KOD XL, U	Vent(exo-), U	PCR product
1	**dU^{SA}TP**, 2 mM	2.5 U	5 U	**98PCR_U^{SA}**
2	**dA^{OP}TP**, 0.5 mM	1.25 U	5 U	**98PCR_A^{OP}**
3	**dG^{PA}TP**, 4 mM	1 U	2 U	**98PCR_G^{PA}**
4	**dC^{CA}TP**, 4 mM	0.5 U	1 U	**98PCR_C^{CA}**

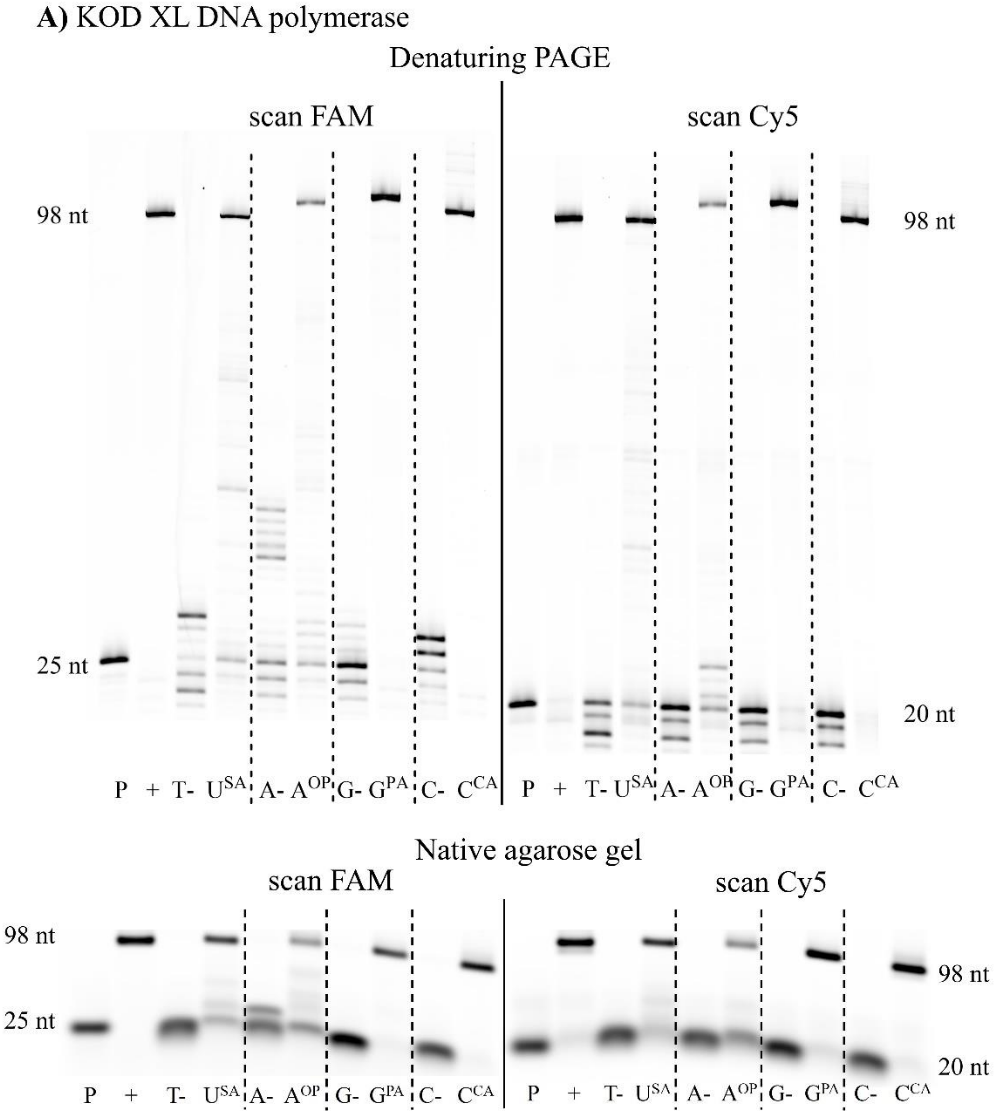

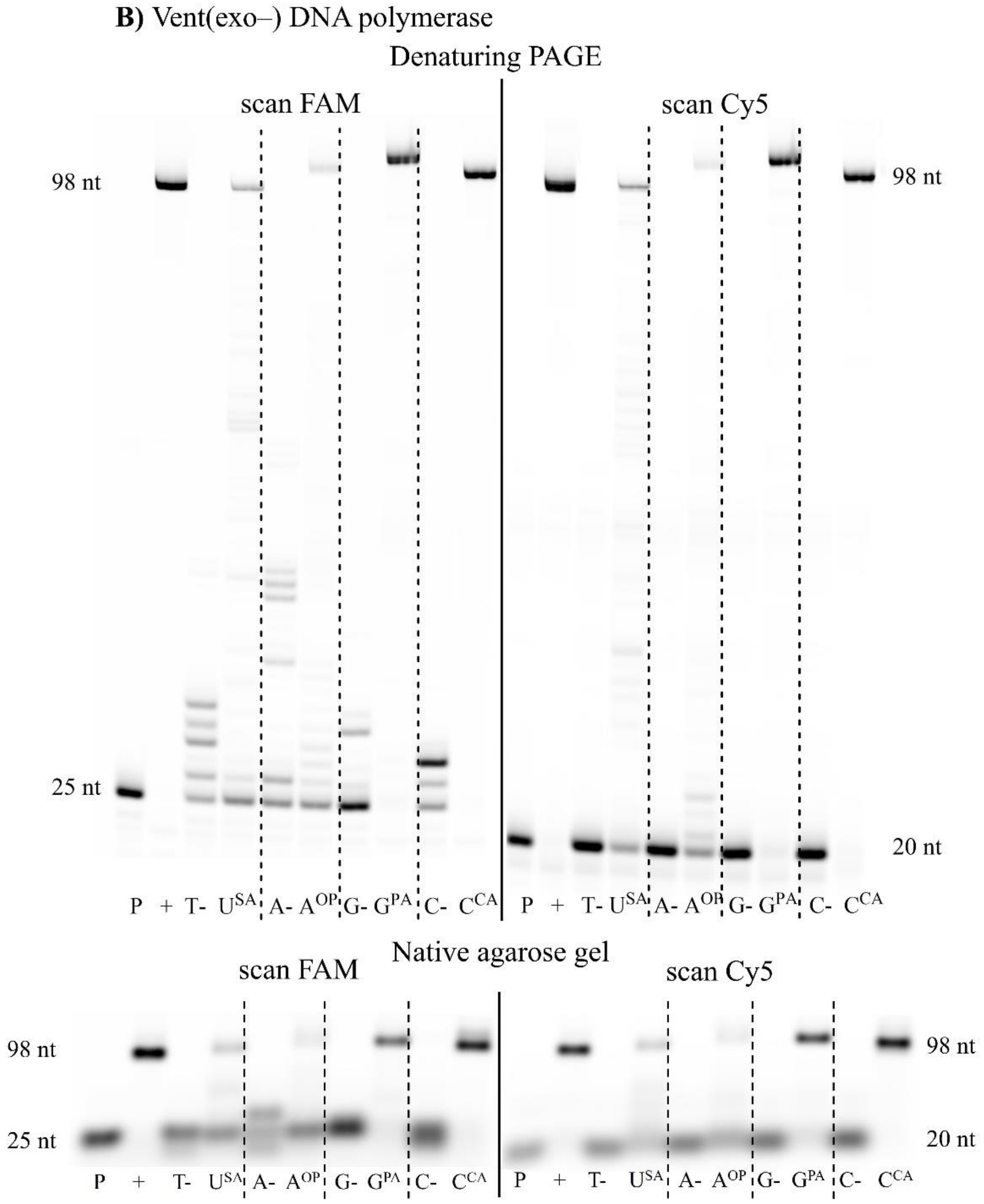

Figure S6. Denaturing PAGE and native agarose gel analysis of PCR reactions with one modified **dN^{R}TP** using 98-mer template Temp^{FVL-A} using: A) KOD XL DNA polymerase, B) Vent(exo-) DNA polymerase: (P) primer; (+) natural dNTPs; (T-) dCTP, dATP, dGTP; (U^{SA}) dCTP, dATP, dGTP, **dU^{SA}TP**; (A-) dCTP, dGTP, dTTP; (A^{OP}) dCTP, **dA^{OP}TP**, dGTP, dTTP; (G-) dCTP, dATP, dTTP; (G^{PA}) dCTP, dATP, **dG^{PA}TP**, dTTP; (C-) dATP, dGTP, dTTP; (C^{CA}) **dC^{CA}TP**, dATP, dGTP, dTTP.

2.9. PCR – Multiple incorporation (two modified dN^{R}TPs)

The reaction mixture (10 µL) contained template Temp$^{FVL\text{-}A}$ (0.5 µM, 0.5 µL), reverse primer Prim^{LT25TH}-FAM and forward primer Prim^{L20}-Cy5 (10 µM, 1 µL each), appropriate set of two natural dNTPs (2 mM, 1 µL), two modified **dN^{R}TP**s (R = SA, OP, PA, CA; N = U, A, G, C) (1 µL each, conditions specified in Table S8), Vent(exo-) or KOD XL DNA polymerase (Table S8) and a corresponding reaction buffer (10X, 1 µL) as supplied by the manufacturer. The positive control contained 0.5 U of KOD XL DNA polymerase and natural dNTPs (2 mM, 2 µL). All reaction mixtures were under cycling protocol: 94 °C for 3 min, followed by 30 cycles at 94 °C for 1 min, 53 °C for 1 min, and 72 °C for 6 min, followed by a final elongation step at 72 °C for 5 min. Samples were analyzed by PAGE and visualized using fluorescence imaging (Figure S7).

Table S8. Reaction condition specifications for PCR (two modified **dN^{R}TP**s).

Lanes in Figure S7	**dN^{R}TP**s, mM	Polymerase, U	PCR product
4	**dA^{OP}TP**, 0.5 mM; **dC^{CA}TP**, 2 mM	KOD XL, 1.25 U	**98PCR_A^{OP}C^{CA}**
7	**dA^{OP}TP**, 0.5 mM; **dG^{PA}TP**, 1 mM	KOD XL, 1.25 U	**98PCR_A^{OP}G^{PA}**
10	**dG^{PA}TP**, 4 mM; **dC^{CA}TP**, 4 mM	Vent(exo-), 2 U	**98PCR_G^{PA}C^{CA}**
13	**dU^{SA}TP**, 2 mM; **dC^{CA}TP**, 2 mM	KOD XL, 2.5 U	**98PCR_U^{SA}C^{CA}**
16	**dU^{SA}TP**, 1 mM; **dA^{OP}TP**, 0.5 mM	KOD XL, 2.5 U	**98PCR_U^{SA}A^{OP}**
19	**dG^{PA}TP**, 2 mM; **dU^{SA}TP**, 2 mM	KOD XL, 2.5 U	**98PCR_G^{PA}U^{SA}**

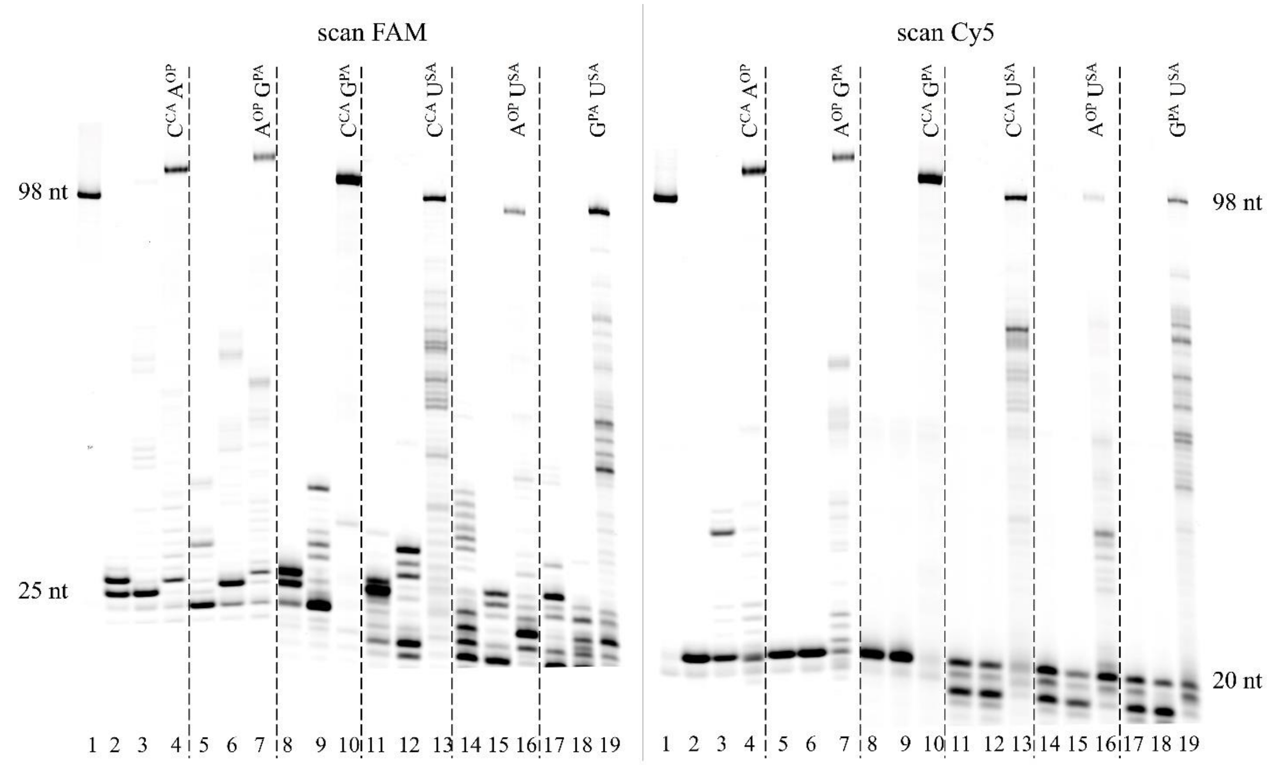

Figure S7. Denaturing PAGE analysis of PCR reactions with two modified **dN$^{\mathbf{R}}$TP**s using 98-mer template Temp^{FVL-A} and either KOD XL or Vent(exo-) DNA polymerase: (lane 1) natural dNTPs; (lane 2) **dA$^{\mathbf{OP}}$TP**, dGTP, dTTP; (lane 3) **dC$^{\mathbf{CA}}$TP**, dGTP, dTTP; (lane 4) **dC$^{\mathbf{CA}}$TP**, **dA$^{\mathbf{OP}}$TP**, dGTP, dTTP; (lane 5) dCTP, **dA$^{\mathbf{OP}}$TP**, dTTP; (lane 6) dCTP, **dG$^{\mathbf{PA}}$TP**, dTTP; (lane 7) dCTP, **dA$^{\mathbf{OP}}$TP**, **dG$^{\mathbf{PA}}$TP**, dTTP; (lane 8) dATP, **dG$^{\mathbf{PA}}$TP**, dTTP; (lane 9) **dC$^{\mathbf{CA}}$TP**, dATP, dTTP; (lane 10) **dC$^{\mathbf{CA}}$TP**, dATP, **dG$^{\mathbf{PA}}$TP**, dTTP; (lane 11) dATP, dGTP, **dU$^{\mathbf{SA}}$TP**; (lane 12) **dC$^{\mathbf{CA}}$TP**, dATP, dGTP; (lane 13) **dC$^{\mathbf{CA}}$TP**, dATP, dGTP, **dU$^{\mathbf{SA}}$TP**; (lane 14) dCTP, dGTP, **dU$^{\mathbf{SA}}$TP**; (lane 15) dCTP, **dA$^{\mathbf{OP}}$TP**, dGTP; (lane 16) dCTP, **dA$^{\mathbf{OP}}$TP**, dGTP, **dU$^{\mathbf{SA}}$TP**; (lane 17) dCTP, dATP, **dG$^{\mathbf{PA}}$TP**; (lane 18) dCTP, dATP, **dU$^{\mathbf{SA}}$TP**; (lane 19) dCTP, dATP, **dG$^{\mathbf{PA}}$TP**, **dU$^{\mathbf{SA}}$TP**;

2.10. PCR – Multiple incorporation (three modified dN$^{\mathbf{R}}$TPs)

The reaction mixture (10 µL) contained template Temp^{FVL-A} (0.5 µM, 0.5 µL), reverse primer Prim^{LT25TH}-FAM and forward primer Prim^{L20}-Cy5 (10 µM, 1 µL each), an appropriate natural dNTP (2 mM, 1 µL), set of modified **dN$^{\mathbf{R}}$TP**s (R = SA, OP, PA, CA; N = U, A, G, C) (1 µL each, conditions specified in Table S9), KOD XL DNA polymerase (2.5 U) and the enzyme reaction

buffer (10X, 1 µL) as supplied by the manufacturer. The positive control contained 0.5 U of KOD XL DNA polymerase and all four natural dNTPs (2 mM, 2 µL). All reaction mixtures were under cycling protocol: 94 °C for 3 min, followed by 30 cycles at 94 °C for 1 min, 53 °C for 1 min, and 72 °C for 6 min, followed by a final elongation step at 72 °C for 5 min. Samples were analyzed by PAGE and visualized using fluorescence imaging (Figure S8).

Table S9. Reaction condition specifications for PCR (three modified **dN^{R}TP**s).

Lanes in Figure S8	**dN^{R}TP**s, mM	PCR product
5	**dC^{CA}TP**, 2 mM; **dG^{PA}TP**, 4 mM; **dA^{OP}TP**, 0.5 mM	**98PCR_C^{CA}G^{PA}A^{OP}**
9	**dA^{OP}TP**, 0.5 mM; **dU^{SA}TP**, 2 mM; **dC^{CA}TP**, 2 mM	**98ON_A^{OP}U^{SA}C^{CA}**
13	**dA^{OP}TP**, 0.5 mM; **dU^{SA}TP**, 2 mM; **dG^{PA}TP**, 4 mM	**98ON_A^{OP}U^{SA}G^{PA}**
17	**dC^{CA}TP**, 4 mM; **dG^{PA}TP**, 4 mM; **dU^{SA}TP**, 2 mM	**98ON_C^{CA}G^{PA}U^{SA}**

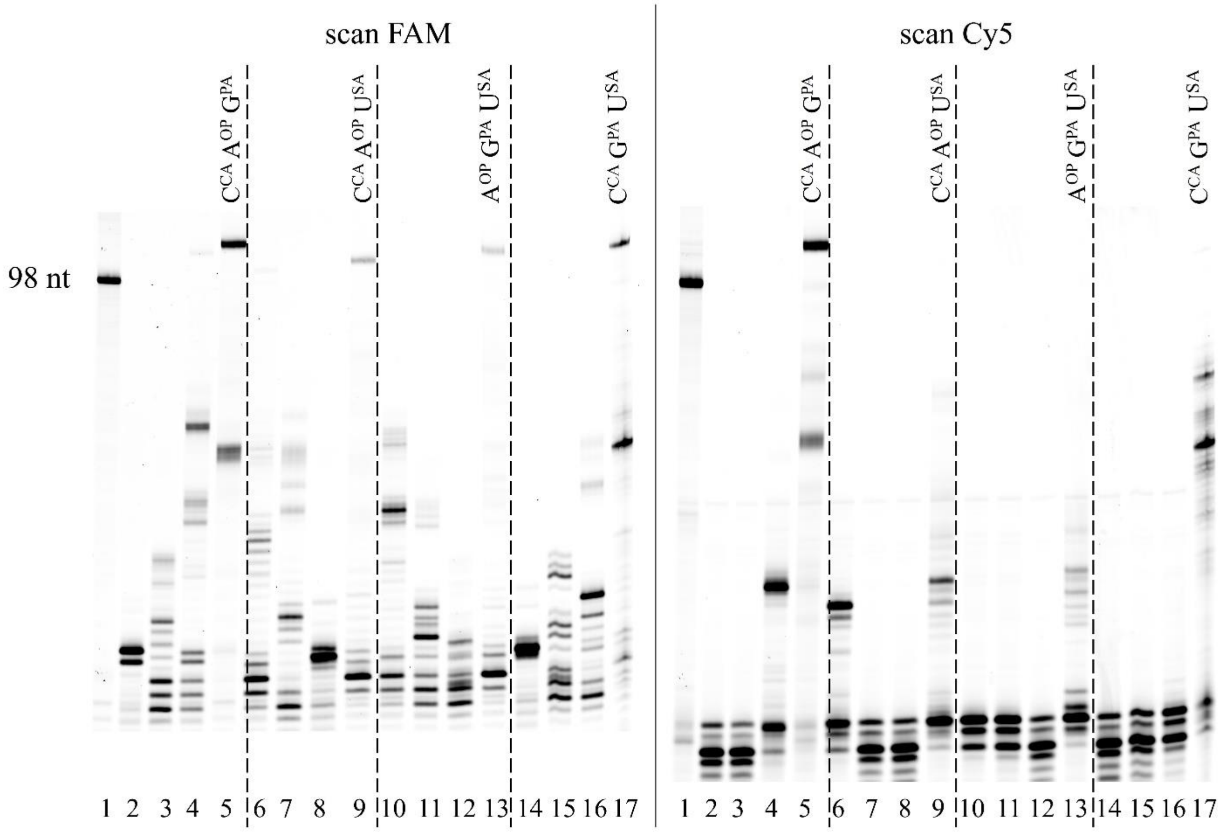

Figure S8. Denaturing PAGE analysis of PCR reactions with three modified **dN^{R}TPs** using 98-mer template Temp^{FVL-A} and KOD XL DNA polymerase: (lane 1) natural dNTPs; (lane 2) **dG^{PA}TP**, **dA^{OP}TP**, dTTP; (lane 3) **dC^{CA}TP**, **dA^{OP}TP**, dTTP; (lane 4) **dC^{CA}TP**, **dG^{PA}TP**, dTTP; (lane 5) **dC^{CA}TP**, **dG^{PA}TP**, **dA^{OP}TP**, dTTP; (lane 6) **dU^{SA}TP**, **dC^{CA}TP**, dGTP; (lane 7) **dA^{OP}TP**, **dC^{CA}TP**, dGTP; (lane 8) **dA^{OP}TP**, **dU^{SA}TP**, dGTP; (lane 9) **dA^{OP}TP**, **dU^{SA}TP**, **dC^{CA}TP**, dGTP; (lane 10) **dU^{SA}TP**, **dG^{PA}TP**, dCTP; (lane 11) **dA^{OP}TP**, **dG^{PA}TP**, dCTP; (lane 12) **dA^{OP}TP**, **dU^{SA}TP**, dCTP; (lane 13) **dA^{OP}TP**, **dU^{SA}TP**, **dG^{PA}TP**, dCTP; (lane 14) **dG^{PA}TP**, **dU^{SA}TP**, dATP; (lane 15) **dC^{CA}TP**, **dU^{SA}TP**, dATP; (lane 16) **dC^{CA}TP**, **dG^{PA}TP**, dATP; (lane 17) **dC^{CA}TP**, **dG^{PA}TP**, **dU^{SA}TP**, dATP.

2.11. PCR – Multiple incorporation (four modified dN^{R}TPs)

The reaction mixture (10 µL) contained template Temp^{FVL-A} (0.5 µM, 0.5 µL), reverse primer Prim^{LT25TH}-FAM and forward primer Prim^{L20}-Cy5 (10 µM, 1 µL each), set of modified **dN^{R}TPs** (1 µL each, conditions specified in Table S10) – **dA^{OP}TP**, **dU^{SA}TP**, **dG^{PA}TP**, **dC^{CA}TP** or **dA^{EIn}TP**, **dU^{EPh}TP**, **dG^{PA}TP**, **dC^{CA}TP**, KOD XL DNA polymerase (2.5 U) and the enzyme reaction buffer (10X, 1 µL) as supplied by the manufacturer. The positive control contained 0.5 U of KOD XL DNA polymerase and all four natural dNTPs (2 mM, 2 µL). All reaction mixtures were under cycling protocol: 94 °C for 3 min, followed by 30 cycles at 94 °C for 1 min, 53 °C for 1 min, and 72 °C for 6 min, followed by a final elongation step at 72 °C for 5 min. Samples were analyzed by PAGE and visualized using fluorescence imaging (Figure S9).

Table S10. Reaction condition specifications for PCR (four modified **dN^{R}TPs**).

Lanes in Figure S9	**dN^{R}TPs**, mM	PCR product
6	**dC^{CA}TP**, 2 mM; **dG^{PA}TP**, 4 mM; **dU^{SA}TP**, 2 mM; **dA^{OP}TP**, 0.5 mM	**98ON_ C^{CA}G^{PA}U^{SA}A^{OP}**
11	**dC^{CA}TP**, 2 mM; **dG^{PA}TP**, 4 mM; **dU^{EPh}TP**, 2 mM; **dA^{EIn}TP**, 2 mM	**98PCR_ C^{CA}G^{PA}U^{EPh}A^{EIn}**

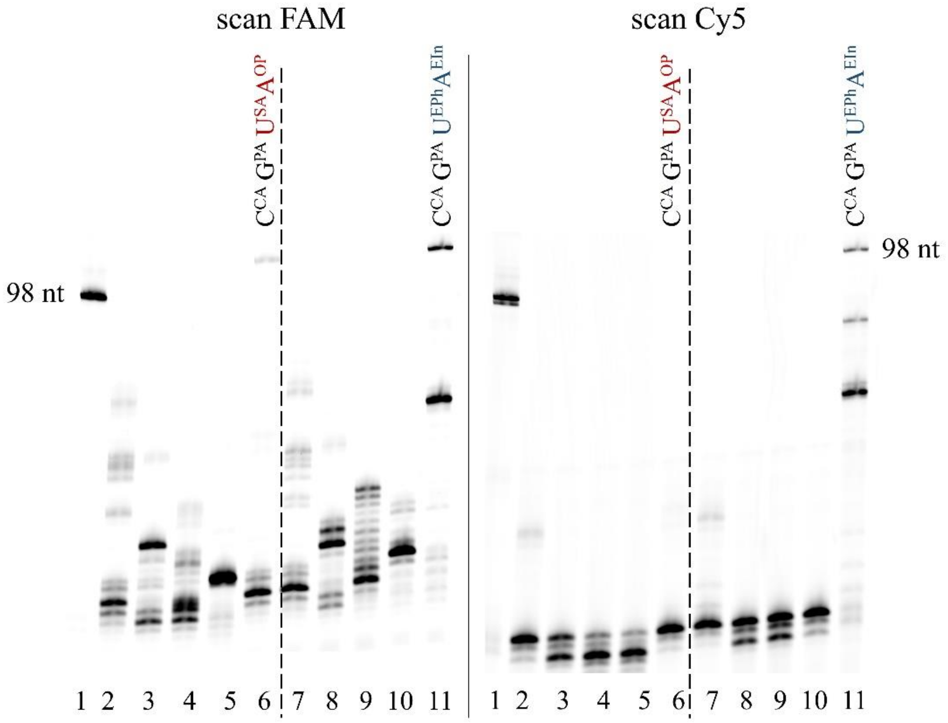

Figure S9. Denaturing PAGE analysis of PCR reactions with four modified **dN^{R}TPs** using 98-mer template Temp$^{FVL\text{-}A}$ and KOD XL DNA polymerase: (lane 1) natural dNTPs; (lane 2) **dU^{SA}TP**, **dG^{PA}TP**, **dC^{CA}TP**; (lane 3) **dA^{OP}TP**, **dG^{PA}TP**, **dC^{CA}TP**; (lane 4) **dA^{OP}TP**, **dU^{SA}TP**, **dC^{CA}TP**; (lane 5) **dA^{OP}TP**, **dU^{SA}TP**, **dG^{PA}TP**; (lane 6) **dA^{OP}TP**, **dU^{SA}TP**, **dG^{PA}TP**, **dC^{CA}TP**; (lane 7) **dU^{EPh}TP**, **dG^{PA}TP**, **dC^{CA}TP**; (lane 8) **dA^{EIn}TP**, **dG^{PA}TP**, **dC^{CA}TP**; (lane 9) **dA^{EIn}TP**, **dU^{EPh}TP**, **dC^{CA}TP**; (lane 10) **dA^{EIn}TP**, **dU^{EPh}TP**, **dG^{PA}TP**; (lane 11) **dA^{EIn}TP**, **dU^{EPh}TP**, **dG^{PA}TP**, **dC^{CA}TP**.

2.12. aPCR – Multiple incorporation (three and four modified dN^{R}TPs)

The reaction mixture (10 µL) contained template Temp$^{FVL\text{-}A}$ (5 µM, 0.5 µL), primer Prim^{LT25TH}-FAM (10 µM, 1 µL), an appropriate natural dNTP (2 mM, 1 µL) or none in the case of four modifications, set of modified **dN^{R}TP**s (1 µL, conditions specified in Table S11), KOD XL DNA polymerase (2.5 U) and the enzyme reaction buffer (10X, 1 µL) as supplied by the manufacturer. The positive control contained 0.5 U of KOD XL DNA polymerase and all four natural dNTPs (2 mM, 2 µL). All reaction mixtures were under cycling protocol: 94 °C for 3 min, followed by 30 cycles at 94 °C for 1 min, 53 °C for 1 min, and 72 °C for 6 min, followed by a final

elongation step at 72 °C for 5 min. Samples were analyzed by PAGE and visualized using fluorescence imaging (Figure S10).

Table S11. Reaction condition specifications for aPCR.

Lanes in Figure S10	**dN^{R}TP**s, mM	aPCR product
5	**dA^{OP}TP**, 0.5 mM; **dU^{SA}TP**, 2 mM; **dC^{CA}TP**, 2 mM	**98ON_A^{OP}U^{SA}C^{CA}**
9	**dA^{OP}TP**, 0.5 mM; **dU^{SA}TP**, 2 mM; **dG^{PA}TP**, 4 mM	**98ON_A^{OP}U^{SA}G^{PA}**
13	**dC^{CA}TP**, 4 mM; **dG^{PA}TP**, 4 mM; **dU^{SA}TP**, 2 mM	**98ON_C^{CA}G^{PA}U^{SA}**
18	**dC^{CA}TP**, 2 mM; **dG^{PA}TP**, 4 mM; **dU^{SA}TP**, 2 mM; **dA^{OP}TP**, 0.5 mM	**98ON_ C^{CA}G^{PA}U^{SA}A^{OP}**
23	**dC^{CA}TP**, 2 mM; **dG^{PA}TP**, 4 mM; **dU^{EPh}TP**, 2 mM; **dA^{EIn}TP**, 2 mM	**98ON_ C^{CA}G^{PA}U^{EPh}A^{EIn}**

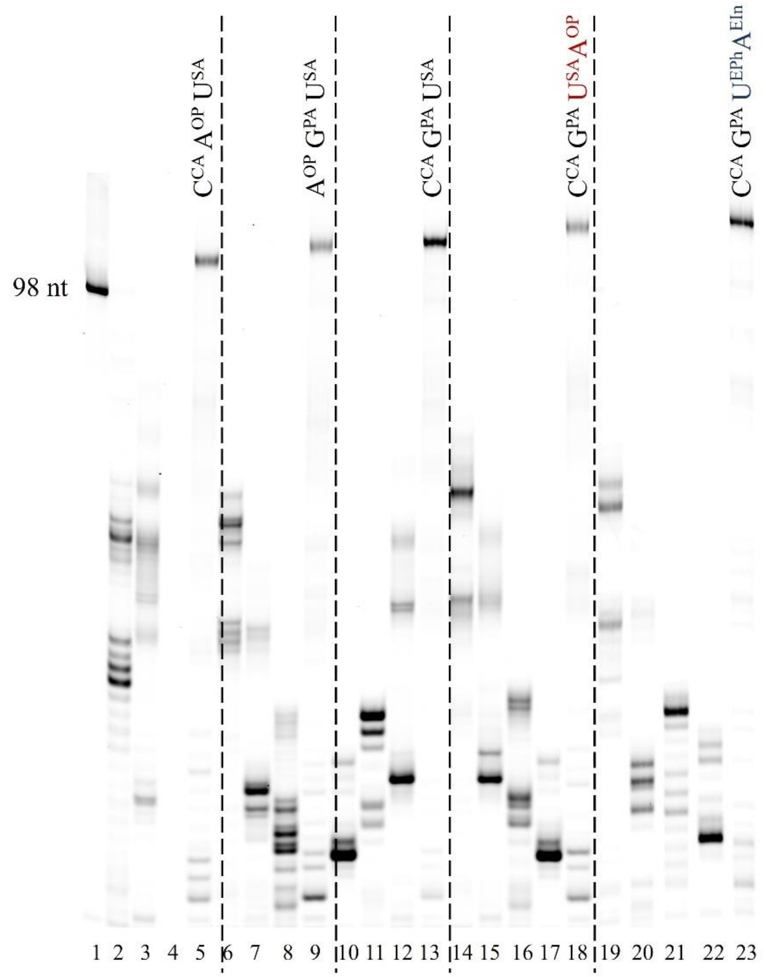

Figure S10. Denaturing PAGE analysis of aPCR reactions with three and four modified **dN^{R}TP**s using 98-mer template Temp^{FVL-A} and KOD XL DNA polymerase: (lane 1) natural dNTPs; (lane 2) **dU^{SA}TP**, **dC^{CA}TP**, dGTP; (lane 3) **dA^{OP}TP**, **dC^{CA}TP**, dGTP; (lane 4) **dA^{OP}TP**, **dU^{SA}TP**, dGTP; (lane 5) **dA^{OP}TP**, **dU^{SA}TP**, **dC^{CA}TP**, dGTP; (lane 6) **dU^{SA}TP**, **dG^{PA}TP**, dCTP; (lane 7) **dA^{OP}TP**, **dG^{PA}TP**, dCTP; (lane 8) **dA^{OP}TP**, **dU^{SA}TP**, dCTP; (lane 9) **dA^{OP}TP**, **dU^{SA}TP**, **dG^{PA}TP**, dCTP; (lane 10) **dG^{PA}TP**, **dU^{SA}TP**, dATP; (lane 11) **dC^{CA}TP**, **dU^{SA}TP**, dATP; (lane 12) **dC^{CA}TP**, **dG^{PA}TP**, dATP; (lane 13) **dC^{CA}TP**, **dG^{PA}TP**, **dU^{SA}TP**, dATP; (lane 14) **dU^{SA}TP**, **dG^{PA}TP**, **dC^{CA}TP**; (lane 15) **dA^{OP}TP**, **dG^{PA}TP**, **dC^{CA}TP**; (lane 16) **dA^{OP}TP**, **dU^{SA}TP**, **dC^{CA}TP**; (lane 17) **dA^{OP}TP**, **dU^{SA}TP**, **dG^{PA}TP**; (lane 18) **dA^{OP}TP**, **dU^{SA}TP**, **dG^{PA}TP**, **dC^{CA}TP**; (lane 19) **dU^{EPh}TP**, **dG^{PA}TP**, **dC^{CA}TP**; (lane 20) **dA^{EIn}TP**, **dG^{PA}TP**, **dC^{CA}TP**; (lane 21) **dA^{EIn}TP**, **dU^{EPh}TP**, **dC^{CA}TP**; (lane 22) **dA^{EIn}TP**, **dU^{EPh}TP**, **dG^{PA}TP**; (lane 23) **dA^{EIn}TP**, **dU^{EPh}TP**, **dG^{PA}TP**, **dC^{CA}TP**.

2.13. Application of fully-modified ssONs for sequencing

2.13.1. Re-PCR of fully-modified ssONs obtained by **aPCR**

To be able to sequence fully-modified ssONs after aPCR, the following approach has been used (Scheme S2): aPCR was performed using 45-nt 5’-(6-FAM)-labelled extended primer Prim$^{\text{Flank-LT25TH}}$-FAM and 98-mer template Temp$^{\text{FVL-A}}$-sC3 modified at 3’-end with three carbon spacer (sC3) preventing any unwanted extension during aPCR (section 2.13.2). The obtained fully-modified 118-mer ssONs **118ON_C^{CA}G^{PA}U^{SA}A^{OP}** and **118ON_C^{CA}G^{PA}U^{EPh}A^{EIn}** (for sequences see Table S1 and S2) were used as templates for re-PCR reaction with 20-nt primers Prim^{Flank}-FAM and Prim$^{\text{L20}}$ and natural dNTPs (section 2.13.6).

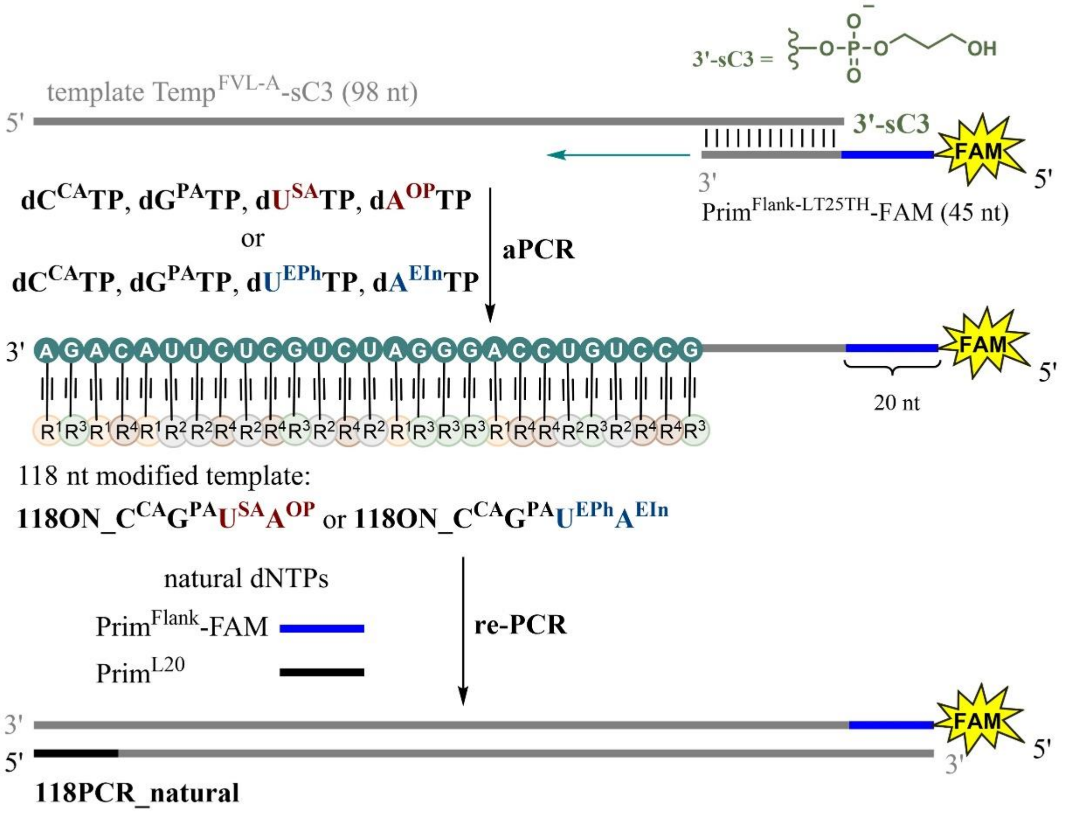

Scheme S2. Asymmetric PCR synthesis with an extended primer Prim$^{\text{Flank-LT25TH}}$-FAM and a set of four modified **dN^{R}TP**s followed by re-PCR with natural dNTPs.

2.13.2. aPCR – synthesis of **118ON_C^{CA}G^{PA}U^{SA}A^{OP}** and **118ON_C^{CA}G^{PA}U^{EPh}A^{EIn}**

The reaction was performed using template Temp^{FVL-A}-sC3, primer Prim$^{Flank-LT25TH}$-FAM, set of modified **dN^{R}TPs** (**dC^{CA}TP**, **dG^{PA}TP**, **dU^{SA}TP**, **dA^{OP}TP** in the case of **118ON_C^{CA}G^{PA}U^{SA}A^{OP}**; and **dC^{CA}TP**, **dG^{PA}TP**, **dU^{EPh}TP**, **dA^{EIn}TP** in the case of **118ON_C^{CA}G^{PA}U^{EPh}A^{EIn}**), and KOD XL DNA polymerase in the amounts described in section 2.12. Reaction mixtures were under cycling protocol: 94 °C for 3 min, followed by 30 cycles at 94 °C for 30 sec, 58 °C for 30 sec, and 72 °C for 7 min, followed by a final elongation step at 72 °C for 5 min. Purification was performed using Agencourt AMPure XP magnetic particles according to the manufacturer's protocol. Samples were analyzed by PAGE and visualized using fluorescence imaging (Figure S11, lanes 3, 5) and further used for re-PCR with natural dNTPs (section 2.13.6).

2.13.3. Re-PCR of fully-modified ssONs obtained by **PEX**

To prove that sequences of fully-modified ssONs used for CD samples preparation (section 3.1) are correct, the following approach has been used (Scheme S3): modified **118DNA_C^{CA}G^{PA}U^{SA}A^{OP}** and **118cDNA_C^{CA}G^{PA}U^{SA}A^{OP}** were obtained by PEX using a primer extended by 20-nt (either 45nt Prim$^{Flank-LT25TH}$-FAM or 40nt Prim$^{Flank-L20}$-FAM) and a 98-mer template (either Temp^{FVL-A}-sC3 or Temp$^{FVL-A_comp}$-sC3) modified at 3'-end with three carbon spacer (sC3) preventing any unwanted extension during PEX (section 2.13.4). Then modified ssONs **118ON_C^{CA}G^{PA}U^{SA}A^{OP}** and **118cON_C^{CA}G^{PA}U^{SA}A^{OP}** were separated from templates using gel extraction method (section 2.13.5) and used as templates for further re-PCR reaction with natural dNTPs and primers Prim^{Flank}-FAM and either Prim^{L20} or Prim^{LT25TH} (section 2.13.6).

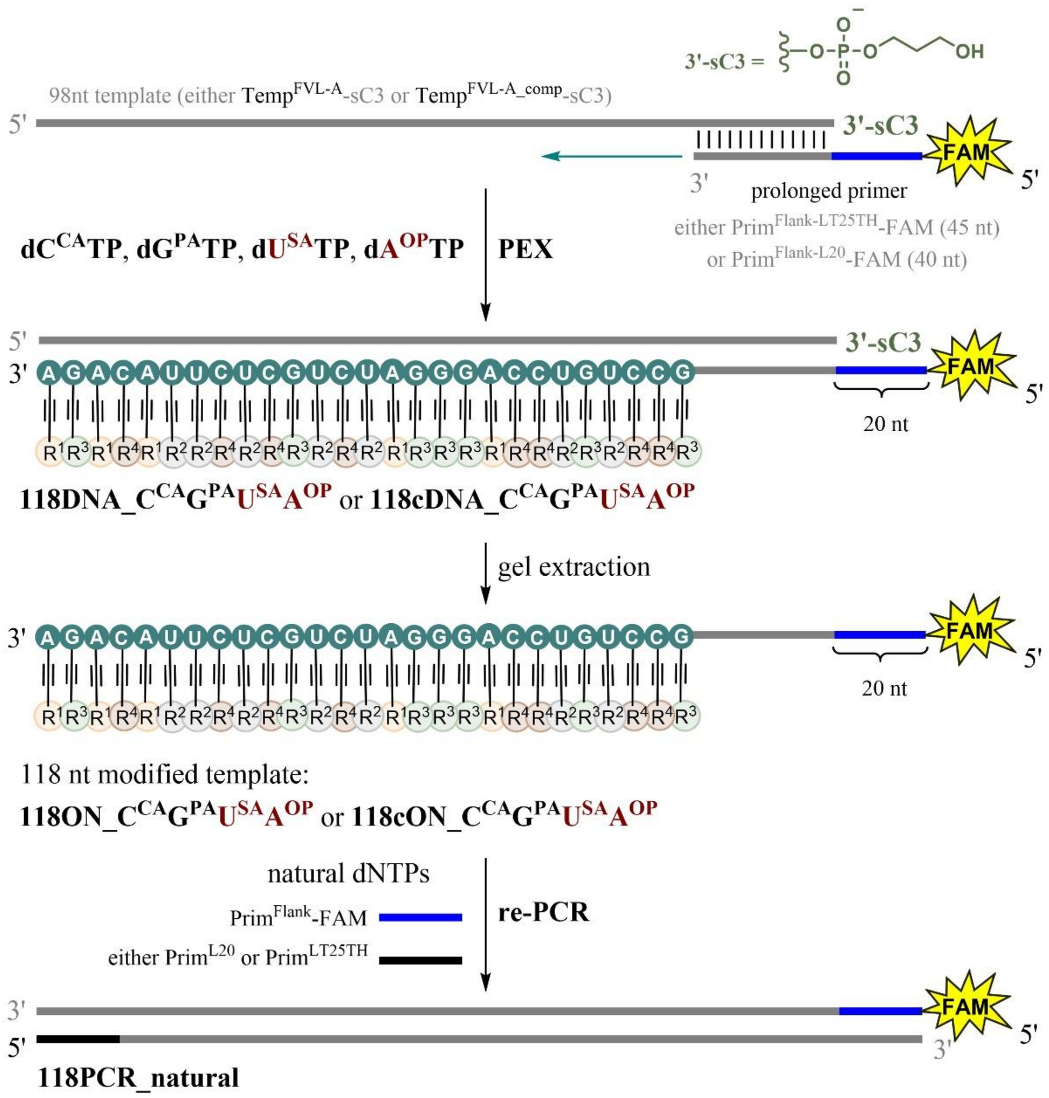

Scheme S3. PEX with an extended primer and a set of four modified **dN^{R}TP**s followed by re-PCR with natural dNTPs.

2.13.4. PEX – synthesis of **118DNA_C^{CA}G^{PA}U^{SA}A^{OP}** and **118cDNA_C^{CA}G^{PA}U^{SA}A^{OP}**

The reaction mixture (10 µL) containing a template (100 µM, 0.5 µL) and a primer (100 µM, 0.5 µL) (Temp$^{FVL\text{-}A}$-sC3 and Prim$^{Flank\text{-}LT25TH}$-FAM in the case of **118DNA_C^{CA}G^{PA}U^{SA}A^{OP}**, Temp$^{FVL\text{-}A_comp}$-sC3 and Prim$^{Flank\text{-}L20}$-FAM in the case of **118cDNA_C^{CA}G^{PA}U^{SA}A^{OP}**), set of four modified **dN^{R}TP**s (R = SA, OP, PA, CA; N = U, A, G, C) (4 mM, 1.25 µL each), KOD XL DNA polymerase (2.5 U), and the enzyme reaction buffer (10X, 1 µL) as supplied by the manufacturer was incubated for 40 minutes at 60 °C. The reaction was stopped by cooling to 8 °C. Further, modified ssONs **118ON_C^{CA}G^{PA}U^{SA}A^{OP}** and **118cON_C^{CA}G^{PA}U^{SA}A^{OP}** (for sequences see

Table S2) were obtained by separation from templates using gel extraction method (section 2.13.5) and visualized using fluorescence imaging (Figure S11, lanes 7, 9).

2.13.5. Gel extraction method

An obtained PEX reaction product was mixed with PAGE stop solution (see General remarks) and loaded on 12.5% preparative denaturing polyacrylamide gel (1.5 mm thick) under denaturing conditions (1 h, 50 °C, 1X TBE buffer). After the run, a modified strand and a template were visualized by UV lamp, and the area containing modified product was cut out. In order to extract the product, Pur-A-Lyze Maxi Dialysis Kit was used. The cut-out piece of gel was placed into a prehydrated Pur-A-Lyze Maxi Dialysis Kit containing 3 mL of 1X TAE buffer. Further, the dialysis kit was placed into a horizontal electrophoretic system in a way that its membrane was in a perpendicular position to the current direction. Then, current was applied (45 min, 100 V) and the product was eluted from the piece of gel and trapped on the dialysis column membrane. To detach DNA molecules from the dialysis column membrane, the current was applied in the reverse direction (1 min, 100 V). The obtained DNA solution was desalted and concentrated using Amicon Ultra-0.5 Centrifugal Filters.

2.13.6. Re-PCR – synthesis of **118PCR_natural** (for further sequencing)

The reaction mixtures (20 µL) contained either **118ON_C^{CA}G^{PA}U^{SA}A^{OP}**, **118ON_C^{CA}G^{PA}U^{EPh}A^{EIn}** or **118cON_C^{CA}G^{PA}U^{SA}A^{OP}** as template (0.5 µM, 1 µL), forward primer Prim^{Flank}-FAM and a reverse primer (Prim^{L20} in the case of **118ON_C^{CA}G^{PA}U^{SA}A^{OP}** and **118ON_C^{CA}G^{PA}U^{EPh}A^{EIn}**, Prim^{LT25TH} in the case of **118cON_C^{CA}G^{PA}U^{SA}A^{OP}**) (10 µM, 2 µL each), all four natural dNTPs (1 mM, 4.5 µL), KOD XL DNA polymerase (1.25 U), and the enzyme reaction buffer (10X, 2 µL) as supplied by the manufacturer. The positive control contained 1.25 U of KOD XL DNA polymerase, template Temp^{FVL-A} (0.5 µM, 1 µL), primers Prim$^{Flank-LT25TH}$-FAM and Prim^{L20} (10 µM, 2 µL each), and natural dNTPs (1 mM, 4.5 µL). All reaction mixtures were under cycling protocol: 94 °C for 3 min, followed by 15 cycles at 94 °C for 30 sec, 50 °C for 1 min, and 72 °C for 1 min, followed by a final elongation step at 72 °C for 5 min. The obtained **118PCR_natural** DNA duplexes were purified using Agencourt AMPure XP

magnetic particles, analyzed by PAGE and visualized using fluorescence imaging (Figure S11, lanes 4, 6, 8, 10; for specifications of **118PCR_natural** samples see Table S12 in section 2.14).

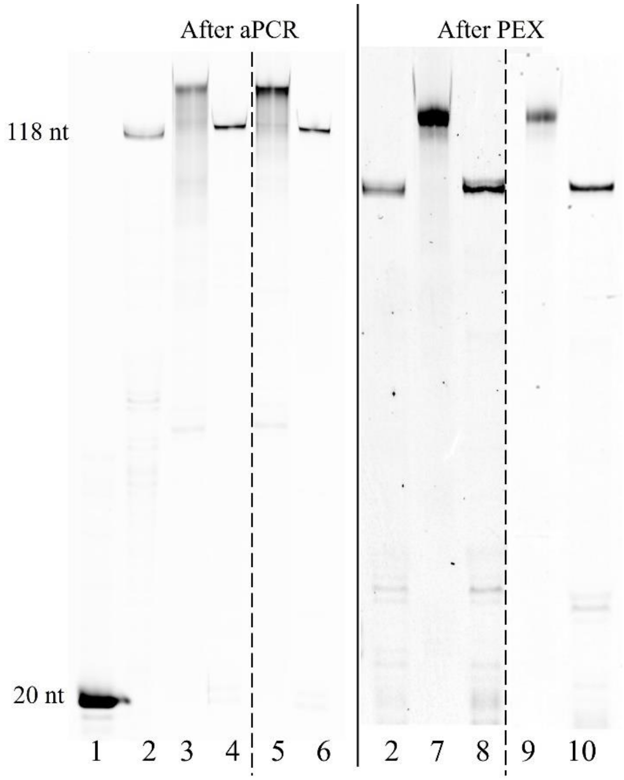

Figure S11. Denaturing PAGE analysis of the performed re-PCRs: (lane 1) primer Prim^{Flank}-FAM; (lane 2) positive control; (lane 3) **118ON_C^{CA}G^{PA}U^{SA}A^{OP}** template obtained by aPCR; (lane 4) **118PCR_natural_1**; (lane 5) **118ON_C^{CA}G^{PA}U^{EPh}A^{EIn}** template obtained by aPCR; (lane 6) **118PCR_natural_2**; (lane 7) **118ON_C^{CA}G^{PA}U^{SA}A^{OP}** template obtained by PEX; (lane 8) **118PCR_natural_3**; (lane 9) **118cON_C^{CA}G^{PA}U^{SA}A^{OP}** template obtained by PEX; (lane 8) **118PCR_natural_4**.

2.14. Sanger sequencing

In order to prepare dsDNA for Sanger sequencing, **118PCR_natural** was obtained using either **118ON_C^{CA}G^{PA}U^{SA}A^{OP}**, **118ON_C^{CA}G^{PA}U^{EPh}A^{EIn}** or **118cON_C^{CA}G^{PA}U^{SA}A^{OP}** as templates by the procedure described in section 2.13 (re-PCRs were performed with non-labelled primers). Samples were purified by Agencourt AMPure XP magnetic particles according to the manufacturer's protocol. Resulting natural 118bp DNA samples (40 ng) were sent for Sanger

sequencing using corresponding primers (5 µL, 5 µM) (Table S12). Primers Prim^{L20}-Seq+ and Prim^{Flank}-Seq+ were used in order to improve sequencing results.

Table S12. Specifications of **118PCR_natural** samples, sent for sequencing.

118PCR_natural	Template	Obtained by	Forward primer	Reverse primer
**118PCR_natural_1**	**118ON_C^{CA}G^{PA}U^{SA}A^{OP}**	aPCR	Prim^{Flank}-Seq+	Prim^{L20}-Seq+
**118PCR_natural_2**	**118ON_C^{CA}G^{PA}U^{EPh}A^{EIn}**	aPCR	Prim^{Flank}-Seq+	Prim^{L20}-Seq+
**118PCR_natural_3**	**118ON_C^{CA}G^{PA}U^{SA}A^{OP}**	PEX	Prim^{Flank}	Prim^{L20}
**118PCR_natural_4**	**118cON_C^{CA}G^{PA}U^{SA}A^{OP}**	PEX	Prim^{Flank}	Prim^{LT25TH}

2.15. Results of Sanger sequencing

2.15.1. 118PCR_natural_1

A) Sequencing chromatogram with forward primer Prim^{Flank}-Seq+

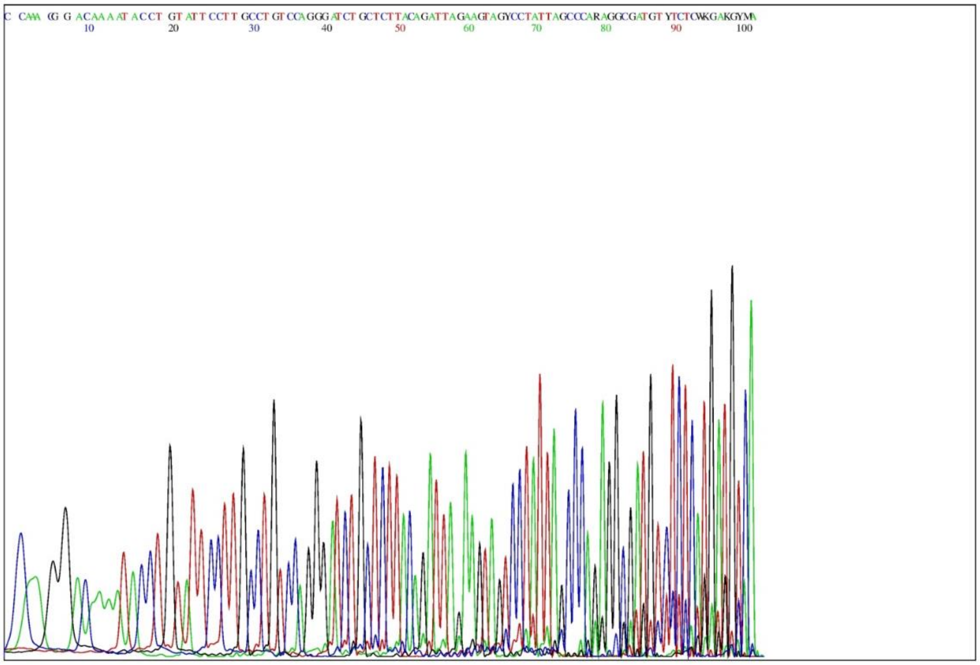

B) Sequencing chromatogram with reverse primer Prim^{L20}-Seq+

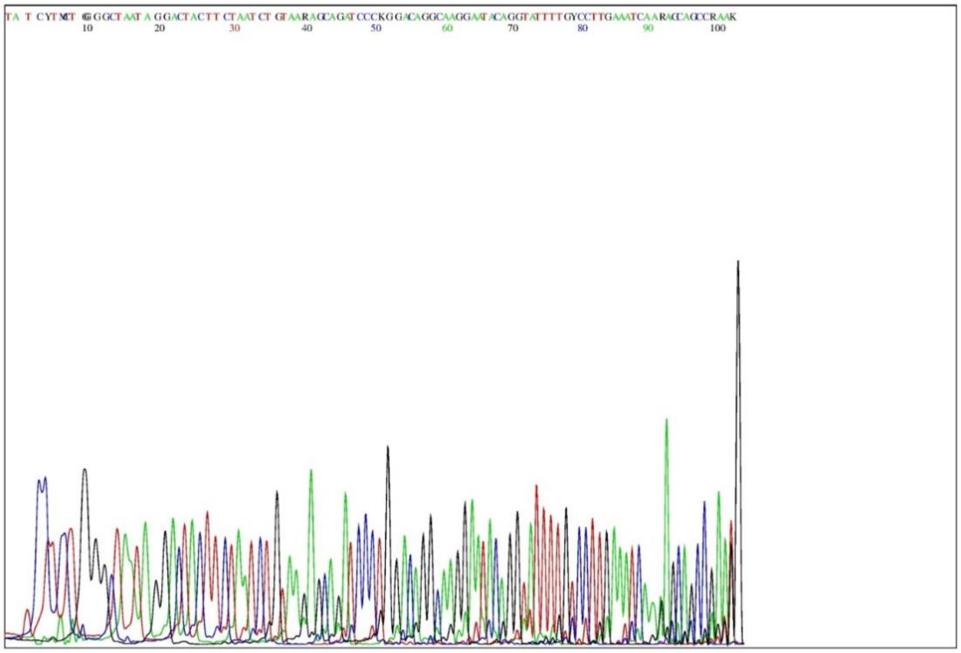

C) Region of properly analyzed sequence shown in blue box

CATTCGGCTGCTCTTGATTTCAAGGACAAAATACCTGTATTCCTTGCCTGTCCAG GGATCTGCTCTTACAGATTAGAAGTAGTCCTATTAGCCCAGAGGCGATGTCTCTC ATGATGTC

2.15.2. 118PCR_natural_2

A) Sequencing chromatogram with forward primer Prim^{Flank}-Seq+

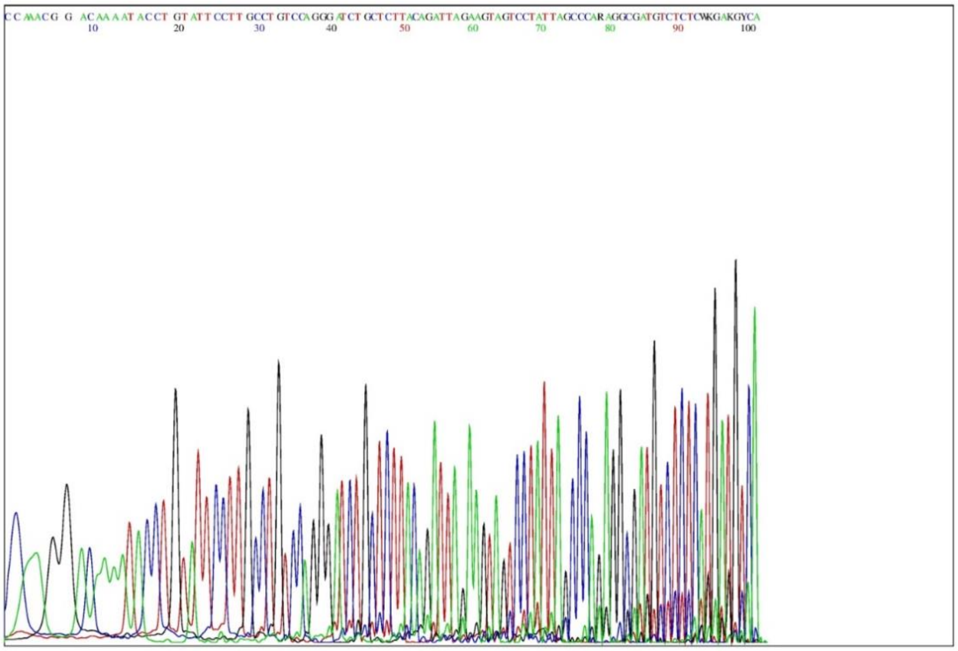

B) Sequencing chromatogram with reverse primer Prim[L20]-Seq+

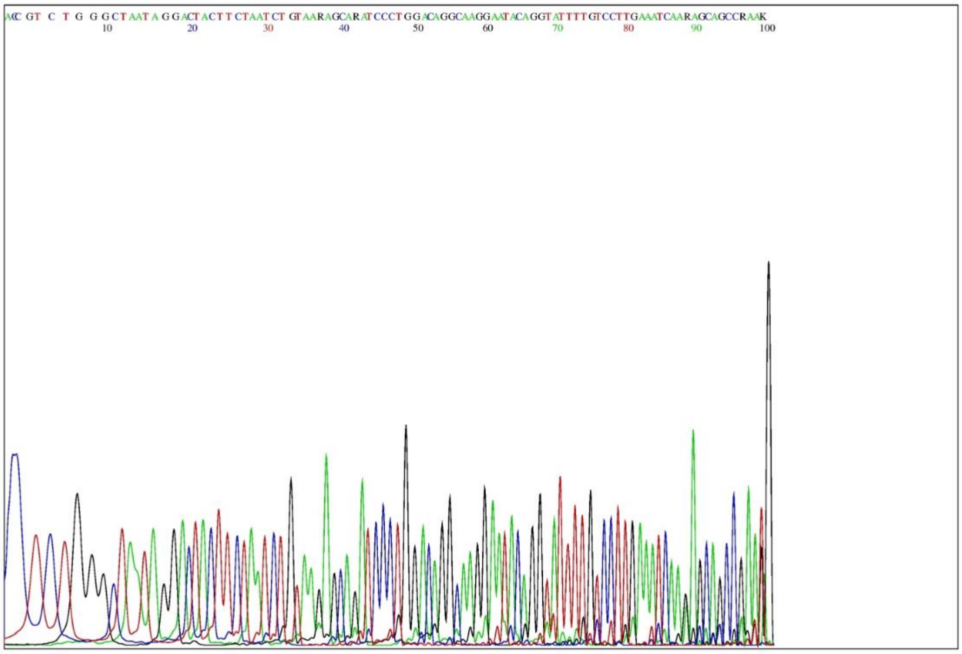

C) Region of properly analyzed sequence shown in blue box

CATTCGGCTGCTCTTGATTTCAAGGACAAAATACCTGTATTCCTTGCCTGTCCAG
GGATCTGCTCTTACAGATTAGAAGTAGTCCTATTAGCCCAGAGGCGATGTCTCTC
ATGATGTC

2.15.3. 118PCR_natural_3

A) Sequencing chromatogram with forward primer Prim^{Flank}

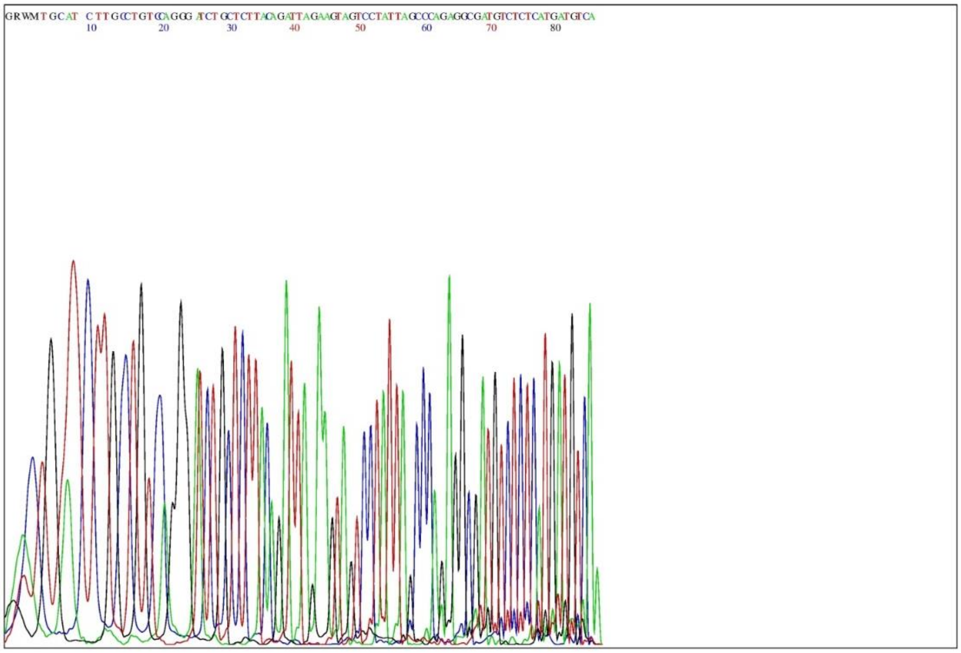

B) Sequencing chromatogram with reverse primer Prim^{L20}

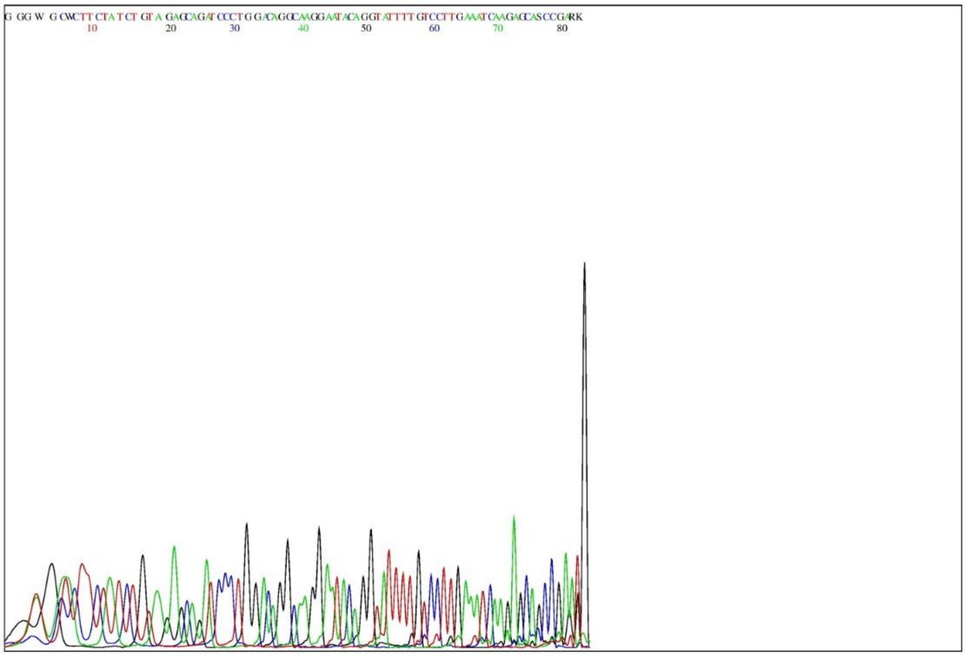

C) Region of properly analyzed sequence shown in blue box

CATTCGGCTGCTCTTGATTTCAAGGACAAAATACCTGTATTCCTTGCCTGTCCAG
GGATCTGCTCTTACAGATTAGAAGTAGTCCTATTAGCCCAGAGGCGATGTCTCTC
ATGATGTC

2.15.4. 118PCR_natural_4

A) Sequencing chromatogram with forward primer Prim^{Flank}

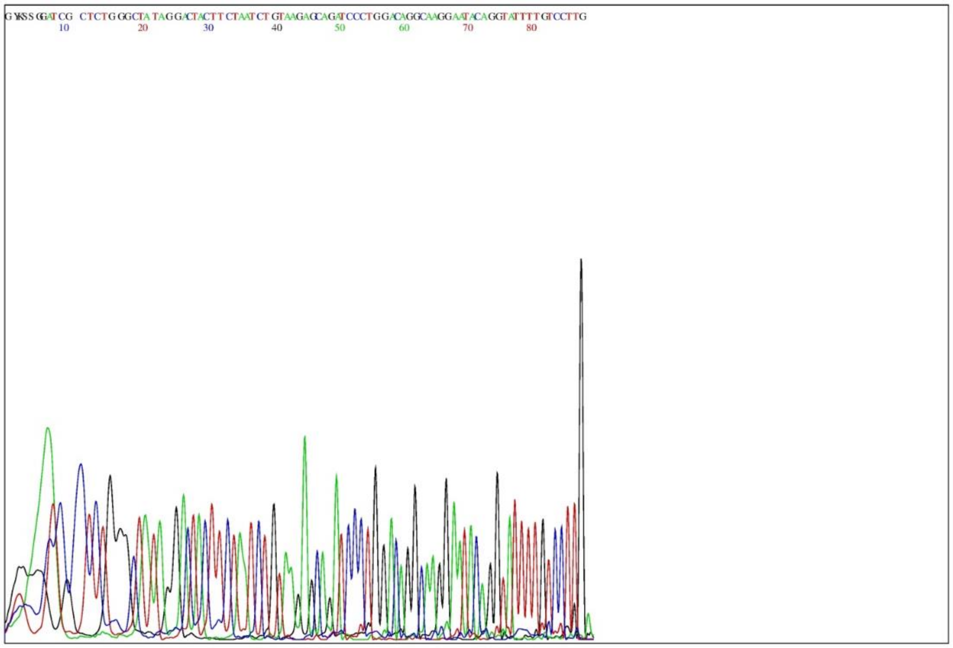

B) Sequencing chromatogram with reverse primer Prim^{LT25TH}

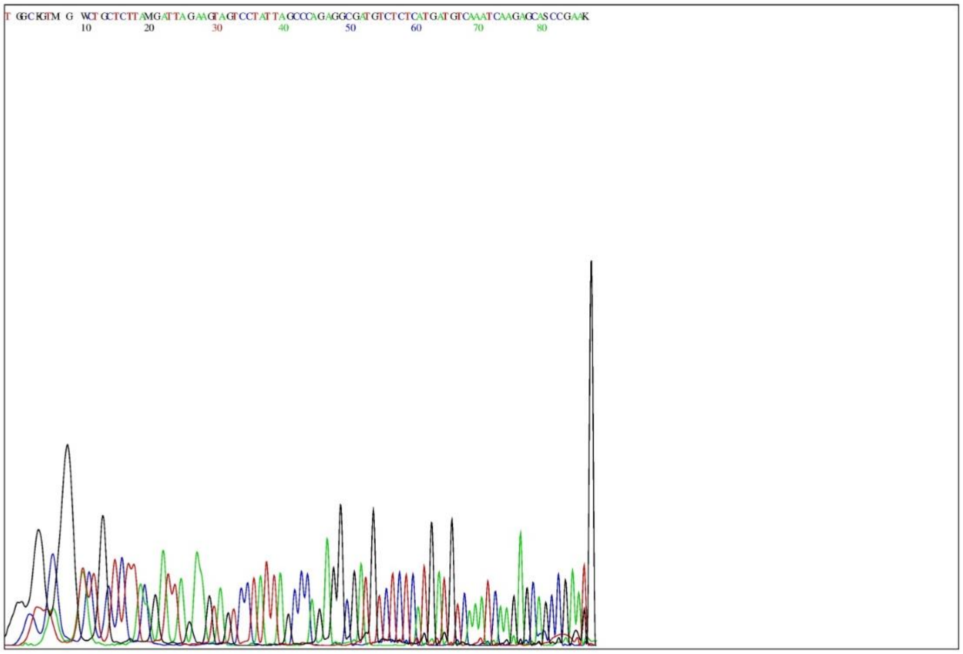

C) Region of properly analyzed sequence shown in blue box

<u>CATTCGGCTGCTCTTGATTTGACATCATGAGAGACATCGC</u>CTCTGGGCTAATAGG
ACTACTTCTAATCTGTAAGAGCAGATCCCTGGACAGGC<u>AAGGAATACAGGTATT</u>
<u>TTGTCCTTG</u>

3. Experimental section – CD spectroscopy and melting temperatures determination

General remarks

The circular dichroism (CD) measurements were performed on a Jasco-1500 spectropolarimeter equipped with Peltier thermostated holder PTC-517 (JASCO Inc. Easton, MD, USA). Firstly, CD spectra were recorded at room temperature in spectral range from 200 nm to 400 nm in rectangular quartz cell with path length 2 mm without Peltier thermostated holder with following experimental setup: standard instrument sensitivity, 1 nm bandwidth, a scanning speed of 10 nm/min, a response time of 8 s and with three accumulations. Temperature dependencies for all samples were measured at temperature range 20 °C – 95 °C with temperature increment 5 °C in spectral range from 200 nm to 350 nm in rectangular quartz cell with path length 2 mm with following experimental setup: standard instrument sensitivity, 1 nm bandwidth, a scanning speed of 10 nm/min, a response time of 8 s and one accumulation. The temperature of the sample was kept constant during each data accumulation and the same experimental set-up was used for temperature increase and decrease. After baseline subtraction, the final data were expressed in differential absorption ΔA. The melting temperatures were calculated using program Sigmaplot 12.5 (Systat software) when sigmoid fitting was applied.

Melting temperature measurements performed using UV-absorption were carried out on Cary 100 Bio UV/VIS Spectrophotometer with temperature controller (Varian). The spectra were recorded in 1 cm rectangular quartz cell, in temperature range 25 °C - 95 °C with temperature increment 1 °C/min under 260 nm detection and were obtained from three cycles (6 ramps in total). Tm values (in °C) were calculated using first negative derivative of intensity over temperature.

3.1. Preparation of the samples

3.1.1. Preparation of **98DNA**

The reaction mixture (20 µL) contained template $Temp^{FVL\text{-}A}$ (0.5 µM, 0.5 µL), reverse primer $Prim^{LT25TH}$ and forward primer $Prim^{L20}$ (10 µM, 4 µL each), natural dNTPs (3 mM, 1 µL), KOD XL DNA polymerase (1.25 U) and a corresponding reaction buffer (10X, 2 µL) as supplied by the manufacturer. The reaction mixture was under cycling protocol: 94 °C for 3 min, followed by 30 cycles at 94 °C for 30 sec, 53 °C for 30 sec, and 72 °C for 1 min, followed by a final elongation step at 72 °C for 5 min. The reaction was repeated 8 times to obtain the sufficient DNA

concentration for measurements. Joined fractions of **98DNA** were purified using QIAquick PCR Purification Kit (Qiagen), concentrated, diluted to 200 μL final volume with an annealing buffer of choice, and set for measurements.

3.1.2. Preparation of **98DNA_C^{CA}G^{PA}U^{SA}A^{OP}**

The reaction mixture (10 μL) containing template Temp^{FVL-A} (100 μM, 0.5 μL), primer Prim^{LT25TH} (100 μM, 0.5 μL), set of four modified **dN^{R}TP**s (R = SA, OP, PA, CA; N = U, A, G, C) (4 mM, 1.25 μL each), KOD XL DNA polymerase (1.25 U), and the enzyme reaction buffer (10X, 1 μL) as supplied by the manufacturer was incubated for 40 min at 60 °C and stopped by cooling to 8 °C. The reaction was repeated 40 times to obtain the sufficient DNA concentration for measurements. Joined fractions of **98DNA_C^{CA}G^{PA}U^{SA}A^{OP}** were purified using Agencourt AMPure XP magnetic particles, concentrated, diluted to 200 μL final volume with an annealing buffer of choice, and set for measurements.

3.1.3. Preparation of **98DNA_dsC^{CA}G^{PA}U^{SA}A^{OP}**

Oligonucleotides **98ON_C^{CA}G^{PA}U^{SA}A^{OP}** and **98cON_C^{CA}G^{PA}U^{SA}A^{OP}** (preparation described below) were annealed together in an annealing buffer of choice under following protocol: 95 °C for 5 min, followed by gradual cooling to 25 °C for 90 min. The annealed product was analyzed by agarose gel containing GelRed intercalator and visualized using fluorescence imaging (Figure S12). The sample was diluted to 200 μL final volume with an annealing buffer of choice and set for measurements.

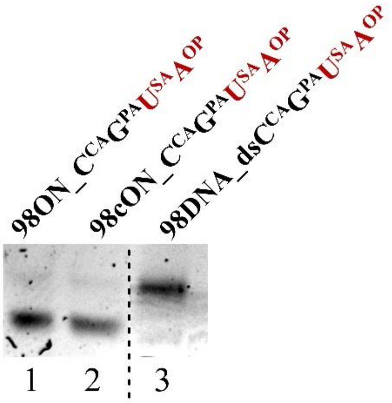

Figure S12. Agarose gel displaying (1) single-stranded **98ON_C^{CA}G^{PA}U^{SA}A^{OP}**, (2) single-stranded **98cON_C^{CA}G^{PA}U^{SA}A^{OP}**, (3) annealed double-stranded **98DNA_dsC^{CA}G^{PA}U^{SA}A^{OP}**.

Preparation of **98ON_C^{CA}G^{PA}U^{SA}A^{OP}** and **98cON_C^{CA}G^{PA}U^{SA}A^{OP}**: To obtain double-stranded products **98DNA_C^{CA}G^{PA}U^{SA}A^{OP}** and **98cDNA_C^{CA}G^{PA}U^{SA}A^{OP}**, PEX reaction, described in section 3.1.2, was repeated 63 times (in the case of **98cDNA_C^{CA}G^{PA}U^{SA}A^{OP}** template Temp$^{FVL\text{-}A_comp}$ and primer Prim^{L20} were used). Then modified strands were separated from the templates using gel extraction method (section 2.13.5).

3.1.4. Preparation of **98DNA_C^{CA}G^{PA}U^{EPh}A^{EIn}**

The reaction mixture (50 µL) containing template Temp$^{FVL\text{-}A}$ (100 µM, 2.13 µL), primer Prim^{LT25TH} (100 µM, 2.13 µL), set of four modified **dN^{R}TP**s (R = EPh, EIn, PA, CA; N = U, A, G, C) (4 mM, 5.2 µL each), KOD XL DNA polymerase (2.5 U), and the enzyme reaction buffer (10X, 5 µL) as supplied by the manufacturer was incubated for 40 min at 60 °C and stopped by cooling to 8 °C. The reaction was repeated 10 times to obtain the sufficient DNA concentration for measurements. Joined fractions of **98DNA_C^{CA}G^{PA}U^{EPh}A^{EIn}** were purified using Agencourt AMPure XP magnetic particles, concentrated, diluted to 200 µL final volume with Tris-HCl buffer (10 mM, 1 mM EDTA, 65 mM NaCl, pH 7.5-8.0), and set for measurements.

3.1.5. Preparation of 98DNA_dsC^{CA}G^{PA}U^{EPh}A^{EIn}

Oligonucleotides **98ON_C^{CA}G^{PA}U^{EPh}A^{EIn}** and **98cON_C^{CA}G^{PA}U^{EPh}A^{EIn}** (preparation described below) were annealed together in Tris-HCl buffer (10 mM, 1 mM EDTA, 100 mM NaCl, pH 7.5-8.0) under following protocol: 95 °C for 5 min, followed by gradual cooling to 25 °C for 90 min. The annealed product was analyzed by agarose gel containing GelRed intercalator and visualized using fluorescence imaging (Figure S13). The sample was diluted to 200 µL final volume with Tris-HCl buffer (10 mM, 1 mM EDTA, 100 mM NaCl, pH 7.5-8.0) and set for measurements.

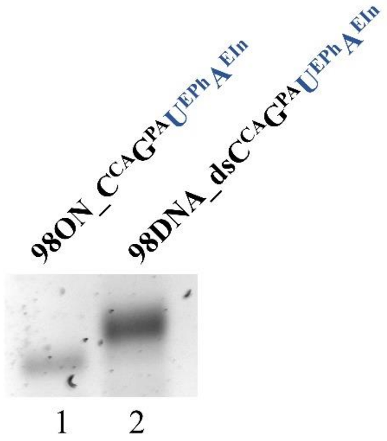

Figure S13. Agarose gel displaying (1) single-stranded **98ON_C^{CA}G^{PA}U^{EPh}A^{EIn}**, (2) annealed double-stranded **98DNA_dsC^{CA}G^{PA}U^{EPh}A^{EIn}**.

Preparation of **98ON_C^{CA}G^{PA}U^{EPh}A^{EIn}** and **98cON_C^{CA}G^{PA}U^{EPh}A^{EIn}**: To obtain double-stranded products **98DNA_C^{CA}G^{PA}U^{EPh}A^{EIn}** and **98cDNA_C^{CA}G^{PA}U^{EPh}A^{EIn}**, PEX reaction, described in section 3.1.4, was repeated 10 times (in the case of **98cDNA_C^{CA}G^{PA}U^{EPh}A^{EIn}** template Temp$^{FVL\text{-}A_comp}$ and primer Prim^{L20} were used). Then modified strands were separated from the templates using gel extraction method (section 2.13.5).

3.2. Circular dichroism (CD) spectroscopy

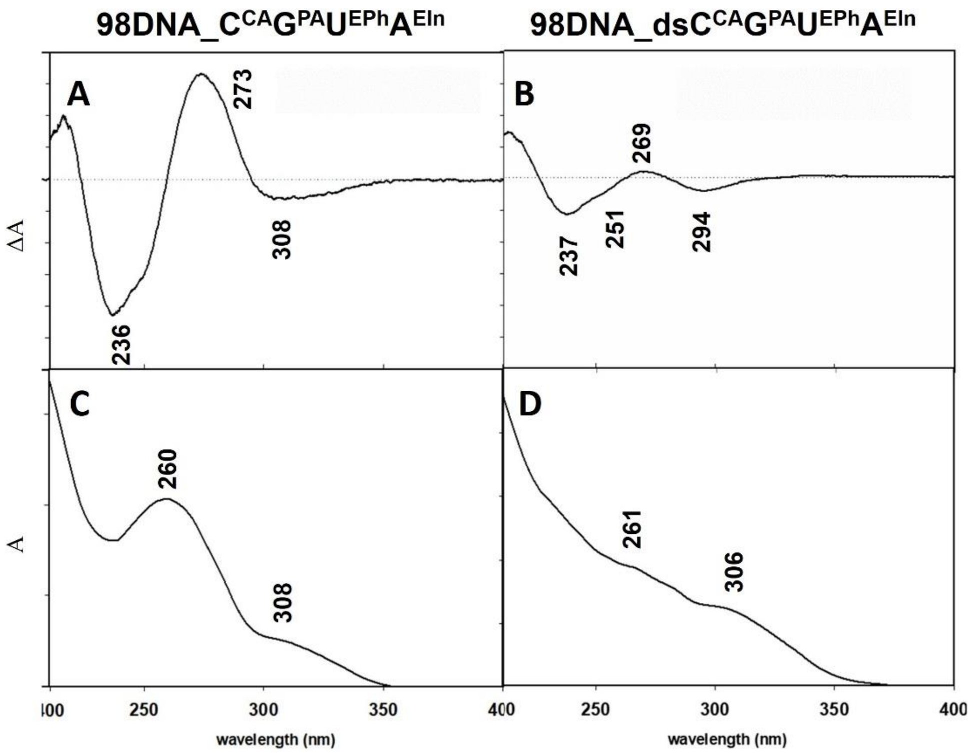

Figure S14. CD spectra (A, B) and UV absorption spectra (C, D) of **98DNA_C^{CA}G^{PA}U^{EPh}A^{EIn}** and **98DNA_dsC^{CA}G^{PA}U^{EPh}A^{EIn}** (buffer composition: 10 mM Tris, 65 mM NaCl, 1 mM EDTA, pH 7.5-8.0; in the case of **98DNA_dsC^{CA}G^{PA}U^{EPh}A^{EIn}** 100 mM NaCl was added to the buffer).

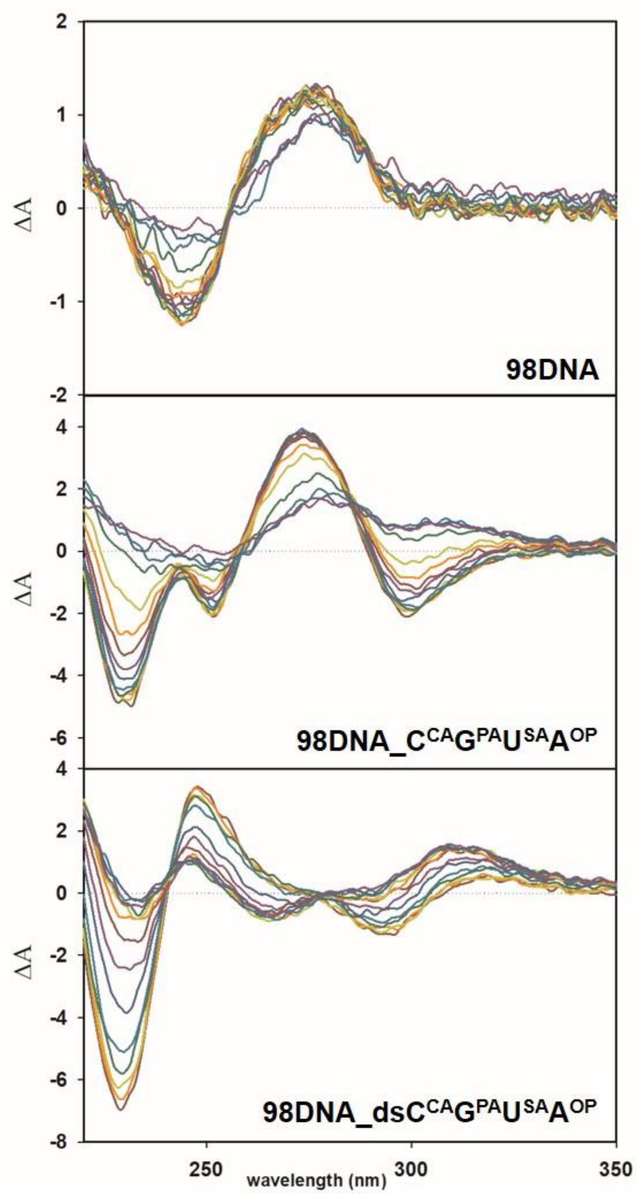

Figure S15. CD spectra reflecting changes within **98DNA**, **98DNA_C^{CA}G^{PA}U^{SA}A^{OP}**, **98DNA_dsC^{CA}G^{PA}U^{SA}A^{OP}** duplexes with increasing temperature (buffer composition: 10 mM Tris, 65 mM NaCl, 1 mM EDTA, pH 7.5-8.0; in the case of **98DNA_dsC^{CA}G^{PA}U^{SA}A^{OP}** 100 mM NaCl was added to the buffer).

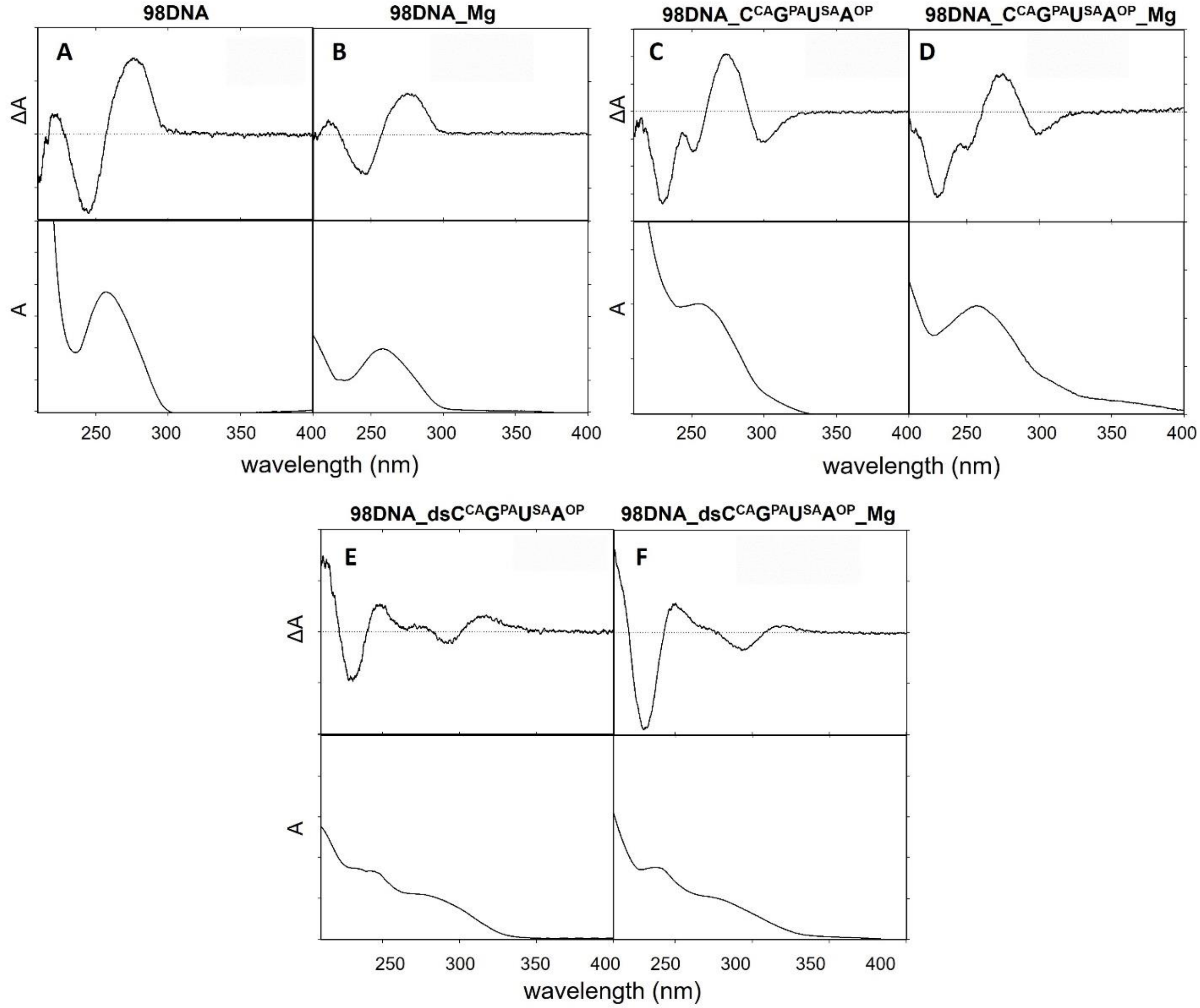

Figure S16. Comparison of CD and UV absorption spectra of **98DNA**, **98DNA_C^{CA}G^{PA}U^{EPh}A^{EIn}**, and **98DNA_dsC^{CA}G^{PA}U^{EPh}A^{EIn}** recorded in the buffer with 2 mM Mg^{2+} (10 mM Tris, 65 mM NaCl, 2 mM $MgSO_4$, pH 7.5-8.0) (B, D, F) and without Mg^{2+} (A, C, E).

3.3. UV-VIS spectroscopy

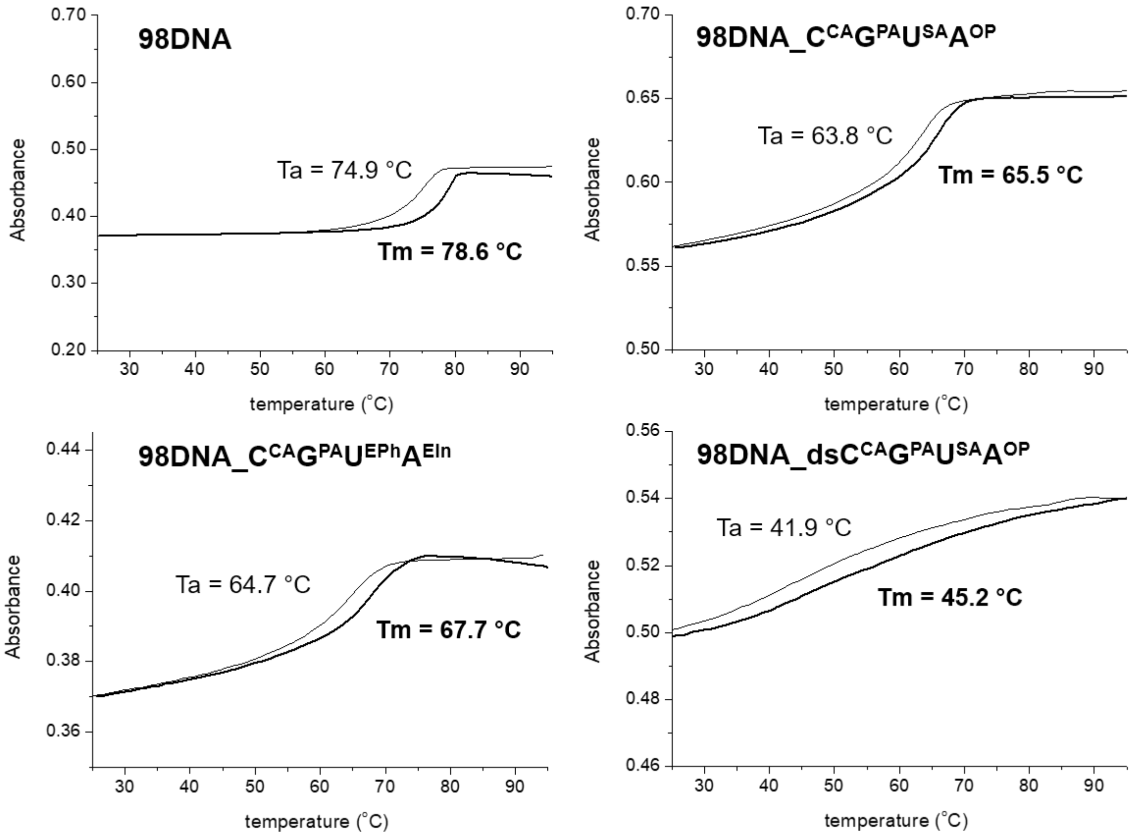

Figure S17. Melting curves with calculated melting (Tm) and annealing (Ta) temperatures of **98DNA**, **98DNA_C^{CA}G^{PA}U^{SA}A^{OP}**, **98DNA_C^{CA}G^{PA}U^{EPh}A^{EIn}**, **98DNA_dsC^{CA}G^{PA}U^{SA}A^{OP}** obtained from UV spectroscopy at 260 nm absorption (buffer composition: 10 mM Tris, 65 mM NaCl, 1 mM EDTA, pH 7.5-8.0; in the case of **98DNA_dsC^{CA}G^{PA}U^{SA}A^{OP}** 100 mM NaCl was added to the buffer).

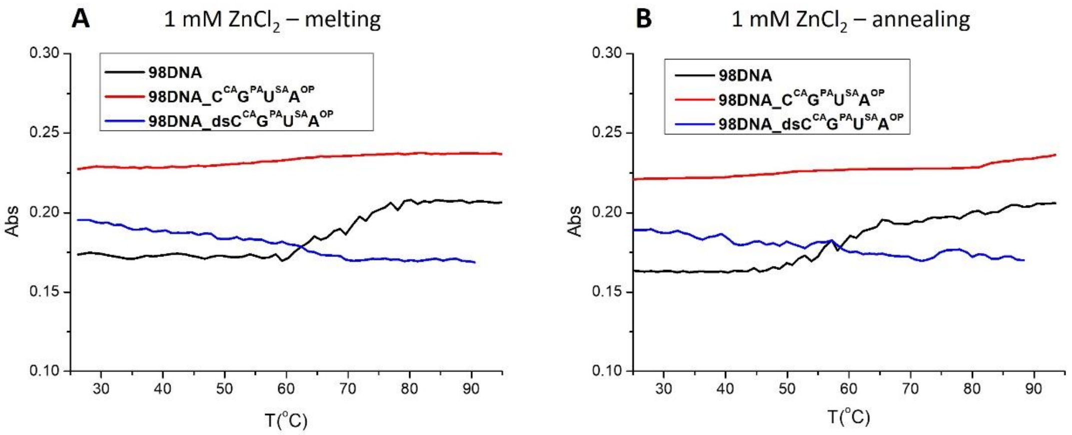

Figure S18. Melting (A) and annealing (B) curves of **98DNA**, **98DNA_C^{CA}G^{PA}U^{SA}A^{OP}**, and **98DNA_dsC^{CA}G^{PA}U^{SA}A^{OP}** in the presence of 1 mM $ZnCl_2$ (buffer composition: 10 mM Tris, 65 mM NaCl, 1 mM $ZnCl_2$, pH 7.5-8.0).

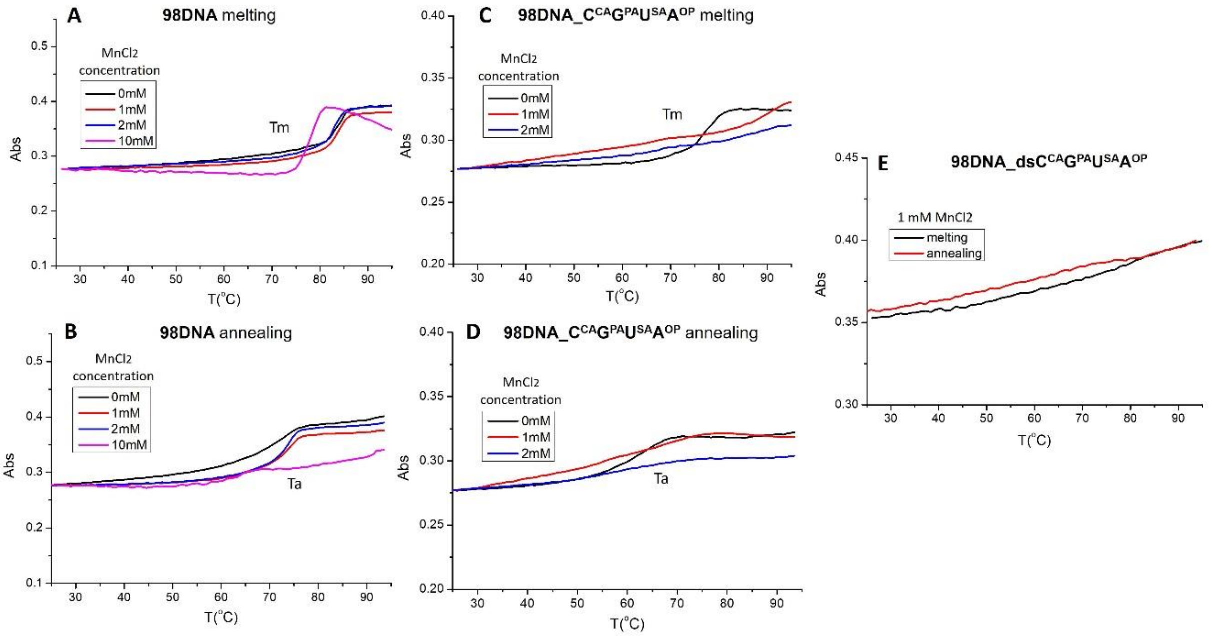

Figure S19. Melting (A, C) and annealing (B, D) curves of **98DNA** and **98DNA_C^{CA}G^{PA}U^{SA}A^{OP}**, and **98DNA_dsC^{CA}G^{PA}U^{SA}A^{OP}** (E) in the presence of $MnCl_2$ (buffer composition: 10 mM Tris, 65 mM NaCl, 1 or 2 mM $MnCl_2$, pH 7.5-8.0)

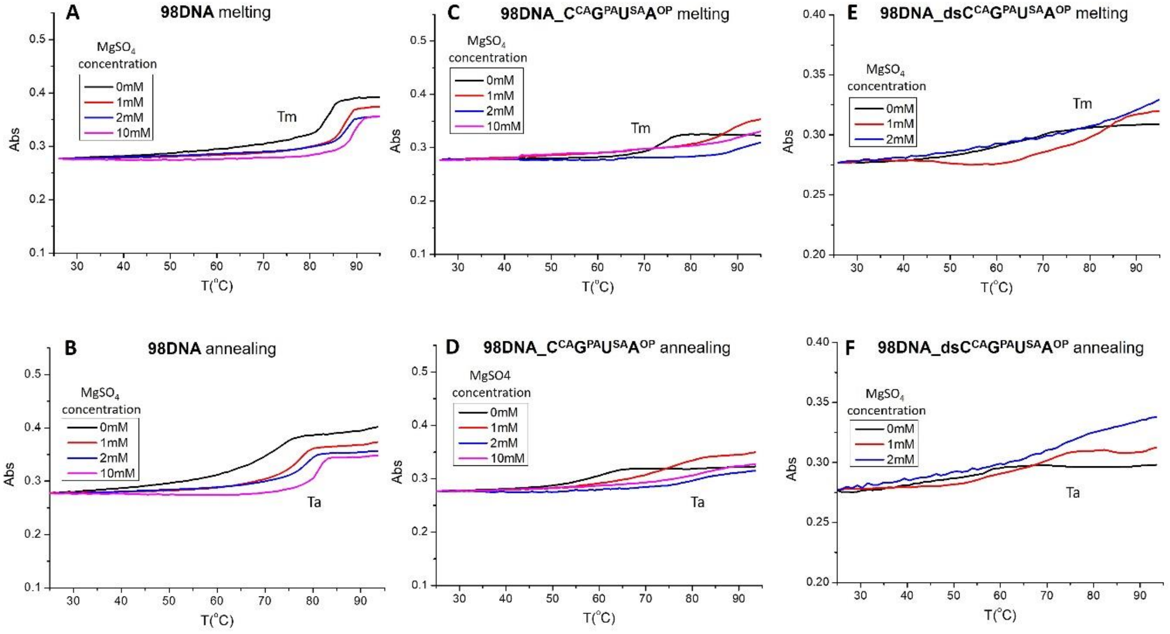

Figure S20. Melting (A, C, E) and annealing (B, D, F) curves of **98DNA**, **98DNA_C^{CA}G^{PA}U^{SA}A^{OP}**, and **98DNA_dsC^{CA}G^{PA}U^{SA}A^{OP}** in the presence of $MgSO_4$ (buffer composition: 10 mM Tris, 65 mM NaCl, 1, 2 or 10 mM $MgSO_4$, pH 7.5-8.0).

Table S13. Melting (Tm) and annealing (Ta) temperatures of natural and modified DNA in presence of different divalent cations (Zn^{2+}, Mn^{2+}, Mg^{2+}) or in the absence of those determined by UV spectroscopy

Divalent cation and its concentration		**98DNA**		**98DNA_C^{CA}G^{PA}U^{SA}A^{OP}**		**98DNA_dsC^{CA}G^{PA}U^{SA}A^{OP}**	
		Tm (°C)	Ta (°C)	Tm (°C)	Ta (°C)	Tm (°C)	Ta (°C)
–		84.1	73.8	73.1	60.1	65.7	55.3
Zn^{2+}	1 mM	61.8	55.2	–	–	–	–
Mn^{2+}	1 mM	84.2	74.4	85.7	52.1	–	–
	2 mM	83.1	73.8	85.2	53.4	–	–
	10 mM	78	64.2	–	–	–	–
Mg^{2+}	1 mM	87.4	77.2	87.6	74.3	84.1	71.4
	2 mM	88.1	78.8	89.7	81.2	85.2	70.3
	10 mM	89.6	80.8	90	82.2	–	–

4. Molecular Dynamics

4.1. Computational models

The native DNA dodecamer sequence, 5'-TCCAGGGATCTG-3', was built in Pymol and subsequently parameterized with the CHARMM36m forcefield[9] using CHARMM-GUI[10]. Modifications to the DNA bases were incorporated using custom scripts based on the doGlycans tool[11], and the corresponding parameters were obtained from CHARMM-GUI for individual modified bases.

The unmodified DNA dodecamer **12DNA**, **12DNA_C^{CA}G^{PA}U^{SA}A^{OP}** containing one strand fully-modified with anionic modifications, and **12DNA_dsC^{CA}G^{PA}U^{SA}A^{OP}** containing both strands fully-modified with anionic modifications were placed in a simulation box of volume 7 nm^3 and solvated with 10738 TIP3P water molecules[12]. In all cases 100 mM NaCl were added to the simulation box, and additional Na^+ ions were added accordingly to maintain electrostatic neutrality.

4.2. Simulation protocol

All systems were initially energy-minimized and equilibrated in the NPT ensemble while maintaining positional restraints on the DNA backbone. Afterwards, twenty 40 ns molecular dynamics simulations (MD) were conducted without restraints in the NPT ensemble to simulate the non-equilibrium unfolding event. Initial velocities were assigned randomly from a Maxwell-Boltzmann distribution at 298.15 K to ensure replica independence. The temperature was maintained at 298.15 K using the V-rescale thermostat[13] with a coupling time of 1 ps, while the pressure was maintained at 1 bar using the C-rescale barostat[14] with a coupling time of 5 ps. The cut-off for electrostatic interactions was set at 1.2 nm, and the long-range contribution was calculated using the PME method[15]. The cut-off for Lennard-Jones interactions was also set at 1.2 nm, and was smoothly switched off between 1 to 1.2 nm using the force-based switching function[16]. The SETTLE algorithm[17] was employed to constrain water molecule geometry, while the LINCS algorithm[18, 19] was used to constrain all other covalent bonds involving hydrogens. All simulations were performed using the Gromacs software, version 2022.2[20].

4.3. Simulation analysis

MD trajectories were analyzed using both the MDAnalysis Python package[21, 22] and Gromacs analysis tools. The time evolution of the root mean squared deviation (RMSD) with respect to an equilibrated unmodified DNA reference was computed for all simulations, considering only backbone heavy atoms. Additionally, the radius of gyration was calculated for all systems. The average value and standard deviation for these two quantities in the 20 replicas of each system are reported in Figure 5. Starting coordinates, topologies, and trajectories, as well as all files needed to reproduce the simulations are available free of charge at https://zenodo.org/record/7998610.

5. Nuclease degradation experiments

For nuclease degradation experiments we have prepared four 31-mer ONs – **31ON**, **31ON_C^{EAlk}G^{EiPr}U^{EPh}A^{EIn}**, **31ON_C^{CA}G^{PA}U^{EPh}A^{EIn}**, and **31ON_C^{CA}G^{PA}U^{SA}A^{OP}** (for sequences see Table S2). Preparation of **31ON_C^{CA}G^{PA}U^{EPh}A^{EIn}** and **31ON_C^{CA}G^{PA}U^{SA}A^{OP}** is described in section 2.6. **31ON_C^{EAlk}G^{EiPr}U^{EPh}A^{EIn}** is a fully-modified ON bearing following hydrophobic modifications:

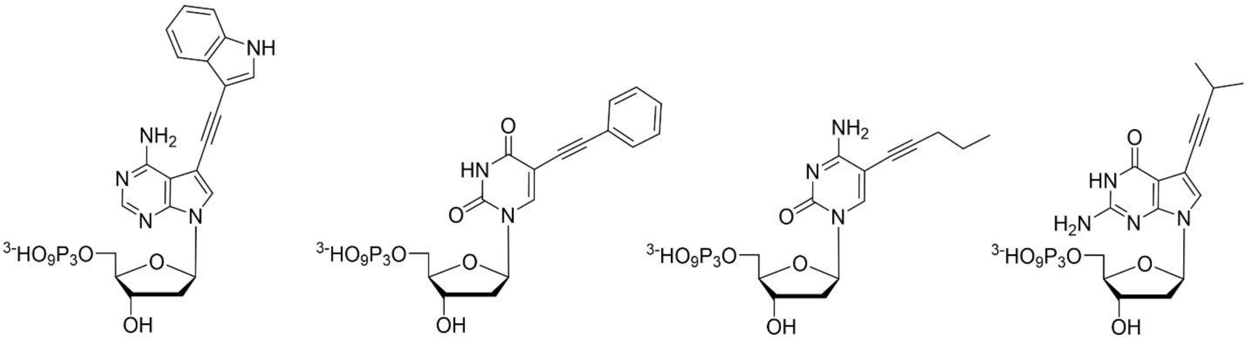

dA^{EIn}TP **dU^{EPh}TP** **dC^{EAlk}TP** **dG^{EiPr}TP**

The synthesis of **31ON_C^{EAlk}G^{EiPr}U^{EPh}A^{EIn}** has been reported previously[8]. **31ON** was synthesized as follows: The reaction mixture (50 µL) containing template Temp^{Prb4basII}-bio (100 µM, 2.13 µL), primer Prim^{248short} (100 µM, 2.13 µL), natural dNTPs (2 mM, 2.5 µL), and Vent(exo-) DNA polymerase (2 U) in the enzyme reaction buffer (10×, 5 µL) as supplied by the manufacturer was incubated for 40 minutes at 60 °C and then stopped by cooling to 8 °C. Product was purified using the DBStv magnetoseparation procedure (see section 2.6).

Further, four 31-mer ONs (0.1µg) – **31ON**, **31ON_C^{EAlk}G^{EiPr}U^{EPh}A^{EIn}**, **31ON_C^{CA}G^{PA}U^{EPh}A^{EIn}**, and **31ON_C^{CA}G^{PA}U^{SA}A^{OP}** were mixed with DNase I (0.01 U), supplied reaction buffer (10X, 1µL) and diluted to total volume of 10µL with DNase-free water. Reaction mixture was incubated at 37°C for 5, 30 and 60 min and terminated by addition of 1µL of 50 mM EDTA followed by incubation at 70 °C for 10 min. The experiment was repeated three times. Results were analyzed by PAGE and visualized using fluorescence imaging (Figure S21). The values of DNA recoveries in each experiment and the average values are calculated in Table S14.

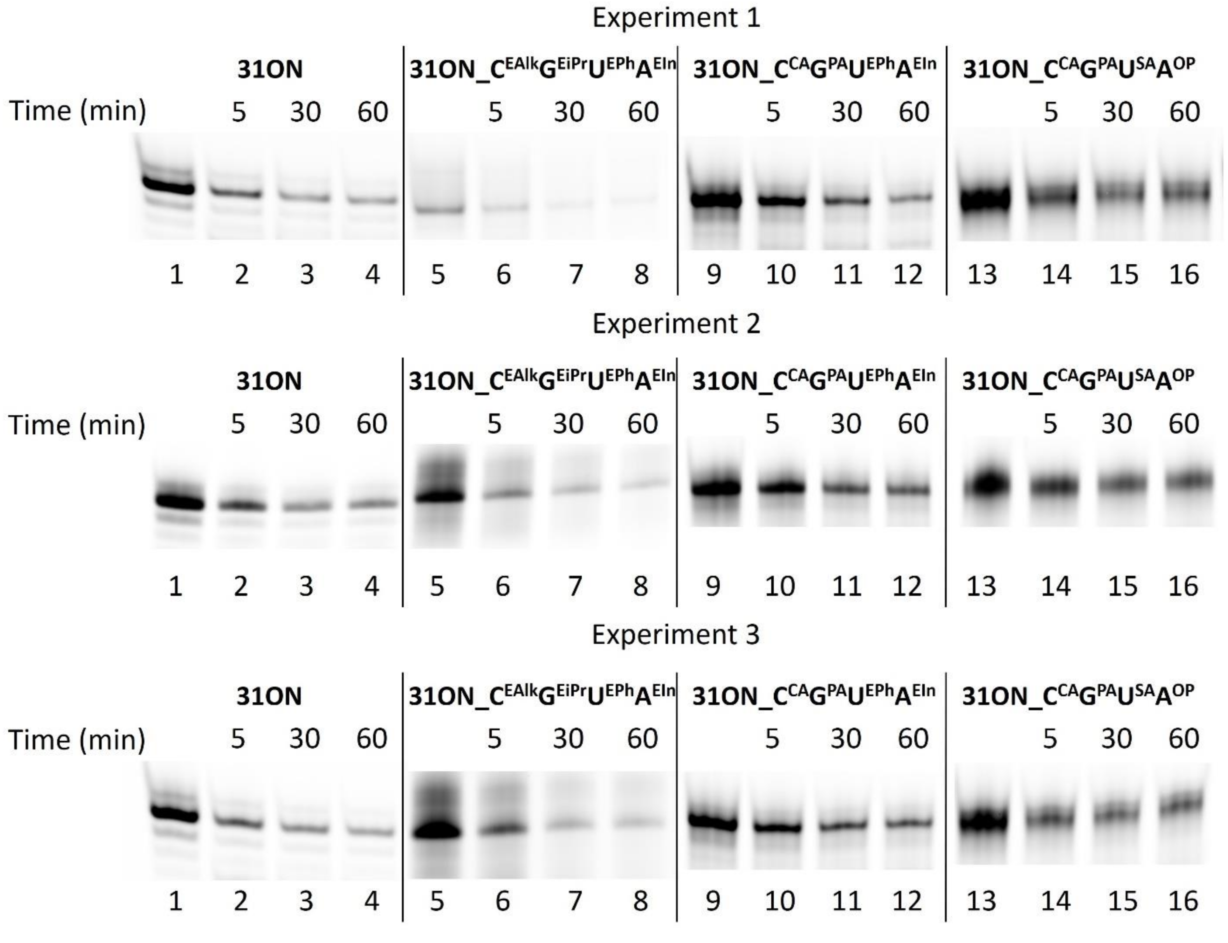

Figure S21. PAGE analysis of stability of natural **31ON** (lanes 2-4), hydrophobic **31ON_C^{EAlk}G^{EiPr}U^{EPh}A^{EIn}** (lanes 6-8), mixed **31ON_C^{CA}G^{PA}U^{EPh}A^{EIn}** (lanes 10-12), and anionic **31ON_C^{CA}G^{PA}U^{SA}A^{OP}** (lanes 14-16) in the presence of 0.01U of DNase I for 5 min (lanes 2, 6, 10, 14), 30 min (lanes 3, 7, 11, 15), and 60 min (lanes 4, 8, 12, 16). Lanes 1, 5, 9, and 13 were not incubated with DNase I. Data presented for three experiments.

Table S14. Recovery (expressed in percent) of natural and hypermodified DNA after incubation with 0.01U of DNase I after a set of three experiment.

DNA title	The number of the experiment	Incubation time with DNase I (0.01U)		
		5 min	30 min	60 min
**31ON**	1	39%	22%	19%
	2	33%	19%	18%
	3	34%	22%	16%
	average	35±3%	21±1%	18±1%
**31ON_C^{EAlk}G^{EiPr}U^{EPh}A^{EIn}**	1	40%	16%	11%
	2	31%	16%	11%
	3	43%	16%	14%
	average	38±5%	16%	12±1%
**31ON_C^{CA}G^{PA}U^{EPh}A^{EIn}**	1	63%	35%	23%
	2	60%	35%	29%
	3	59%	40%	35%
	average	61±2%	37±2%	29±5%
**31ON_C^{CA}G^{PA}U^{SA}A^{OP}**	1	66%	50%	52%
	2	70%	54%	52%
	3	56%	47%	48%
	average	64±6%	50±3%	50±2%

6. Copies of MALDI-TOF mass spectra

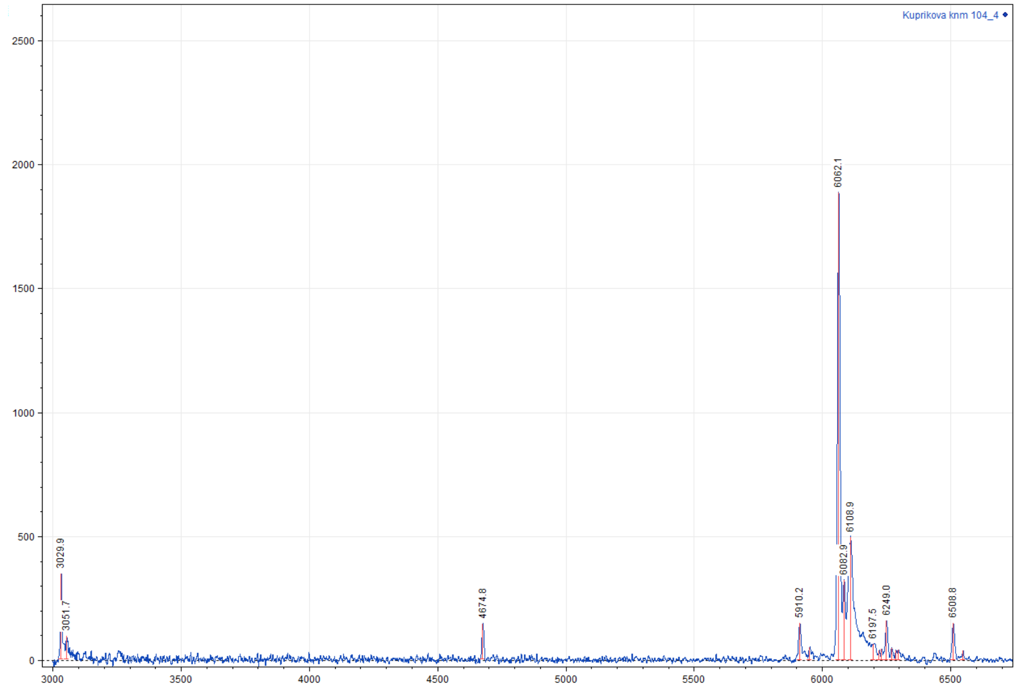

Figure S22. MALDI-TOF spectrum of **19ON_C^CA^**: calculated: 6060.9 Da; found: 6062.1 Da; Δ = 1.2 Da.

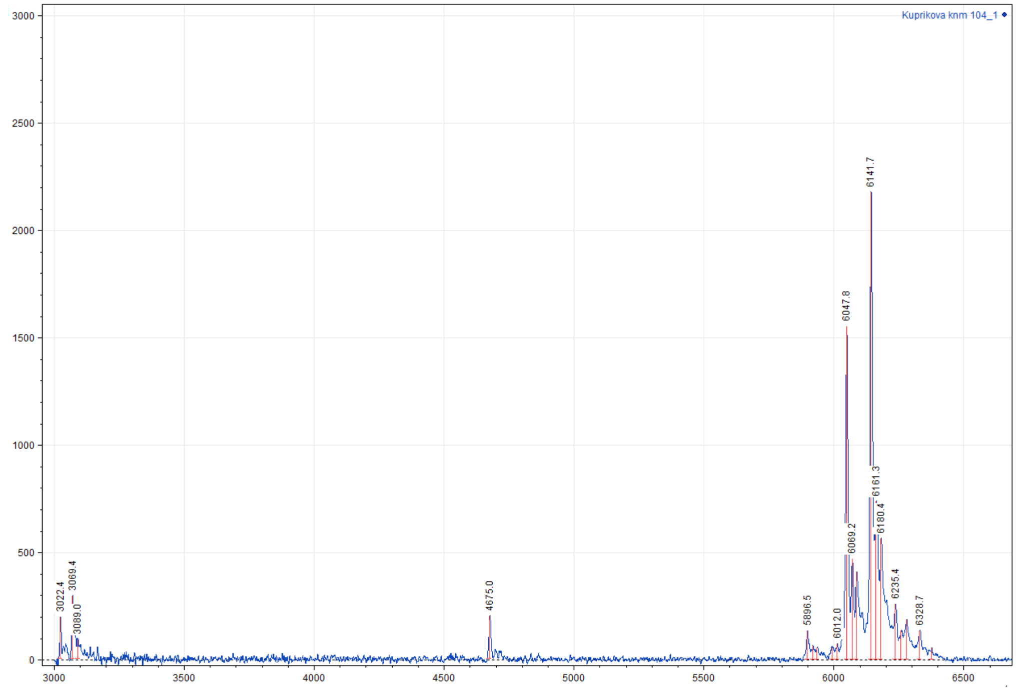

Figure S23. MALDI-TOF spectrum of **19ON_G^PA^**: calculated: 6046.9 Da; found: 6047.8 Da; Δ = 0.9 Da.

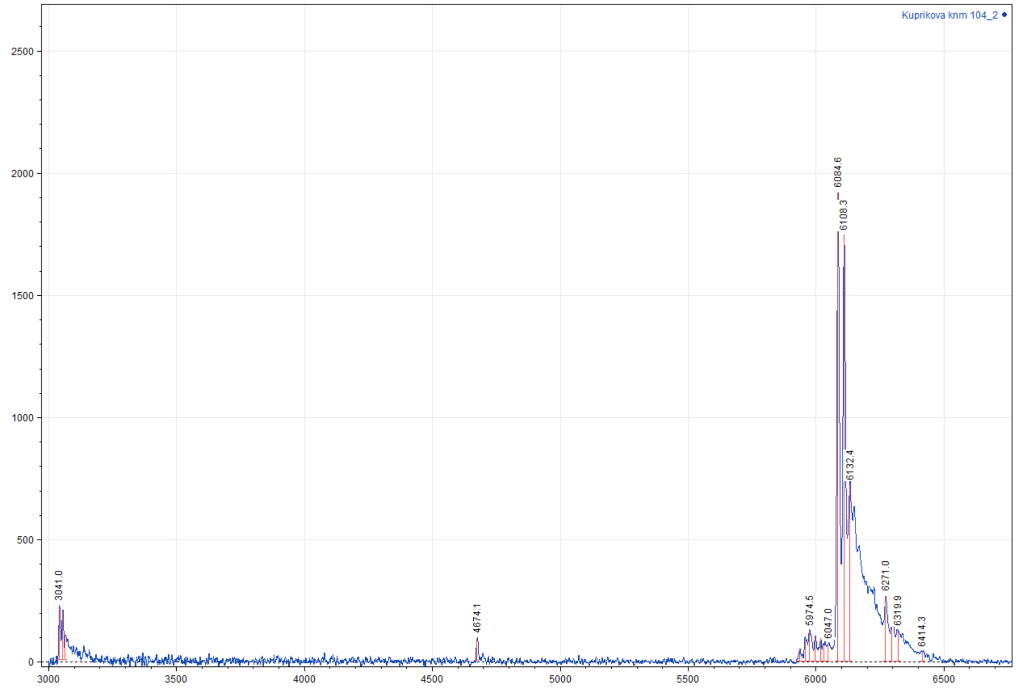

Figure S24. MALDI-TOF spectrum of **19ON_A^{OP}**: calculated: 6107.9 Da; found: 6108.3 Da; Δ = 0.4 Da.

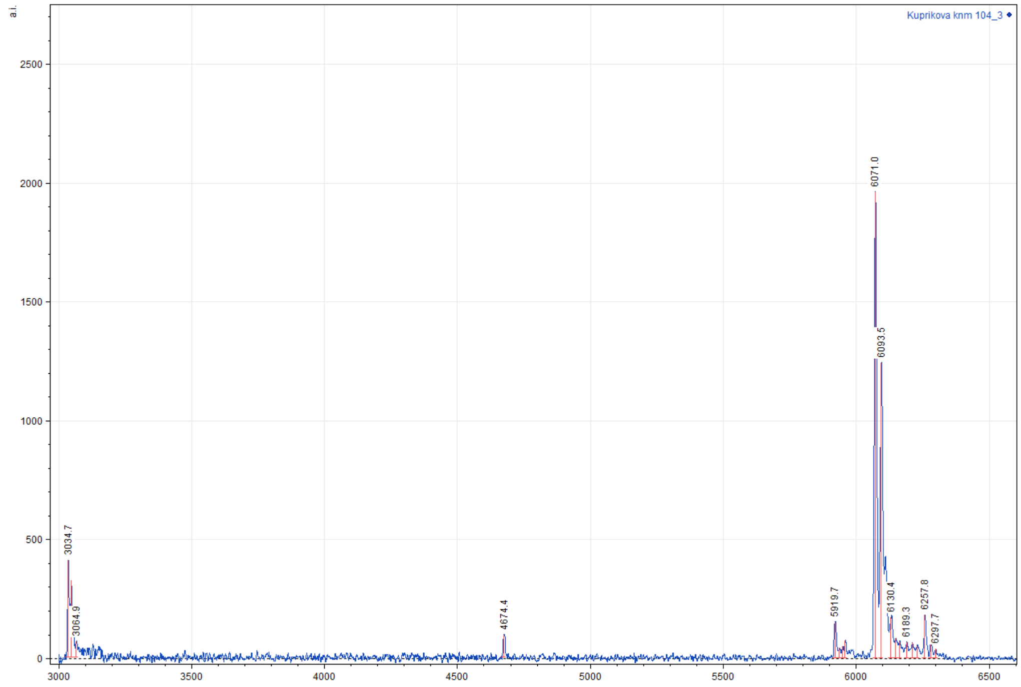

Figure S25. MALDI-TOF spectrum of **19ON_U^{SA}**: calculated: 6069.9 Da; found: 6071.0 Da; Δ = 1.1 Da.

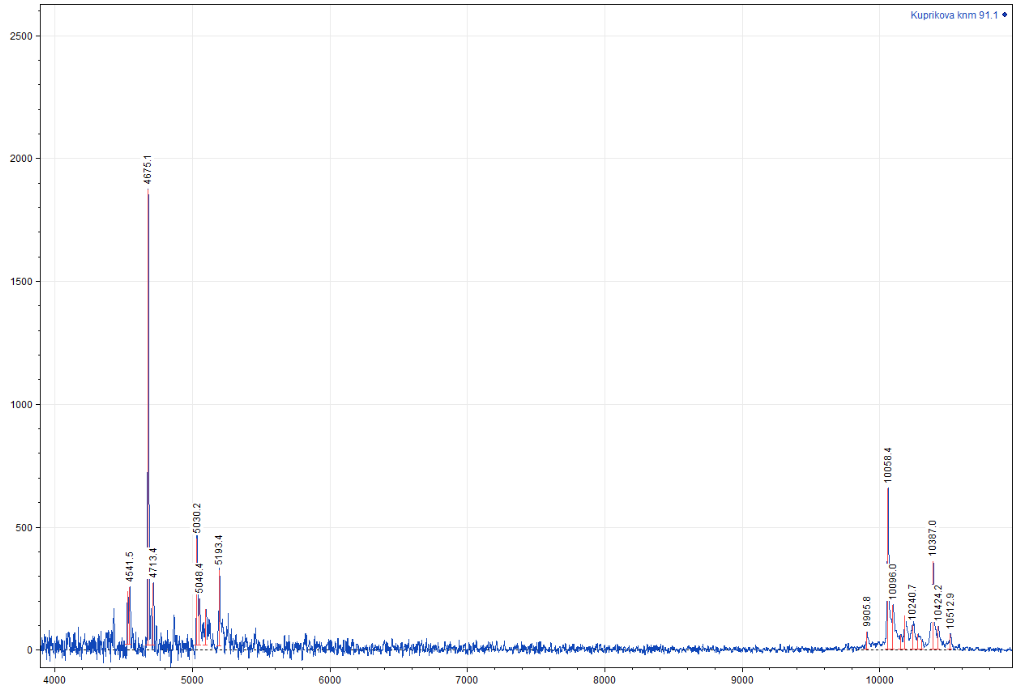

Figure S26. MALDI-TOF spectrum of **31ON_C^{CA}**: calculated: 10057.3 Da; found: 10058.4 Da; Δ = 1.1 Da.

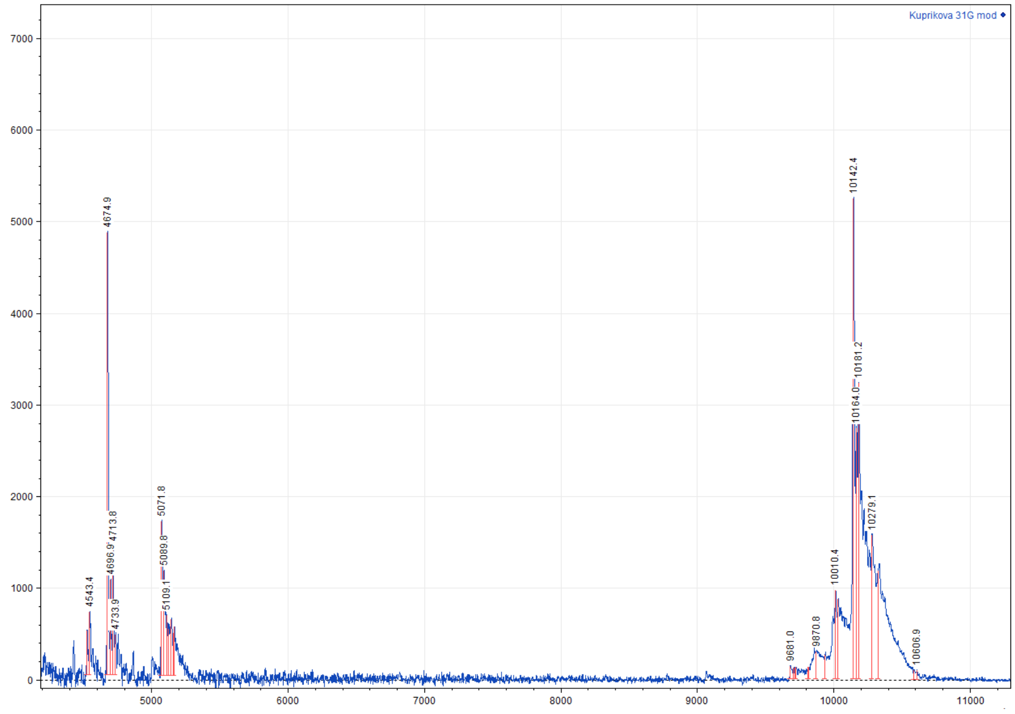

Figure S27. MALDI-TOF spectrum of **31ON_G^{PA}**: calculated: 10141.3 Da; found: 10142.4 Da; Δ = 1.1 Da.

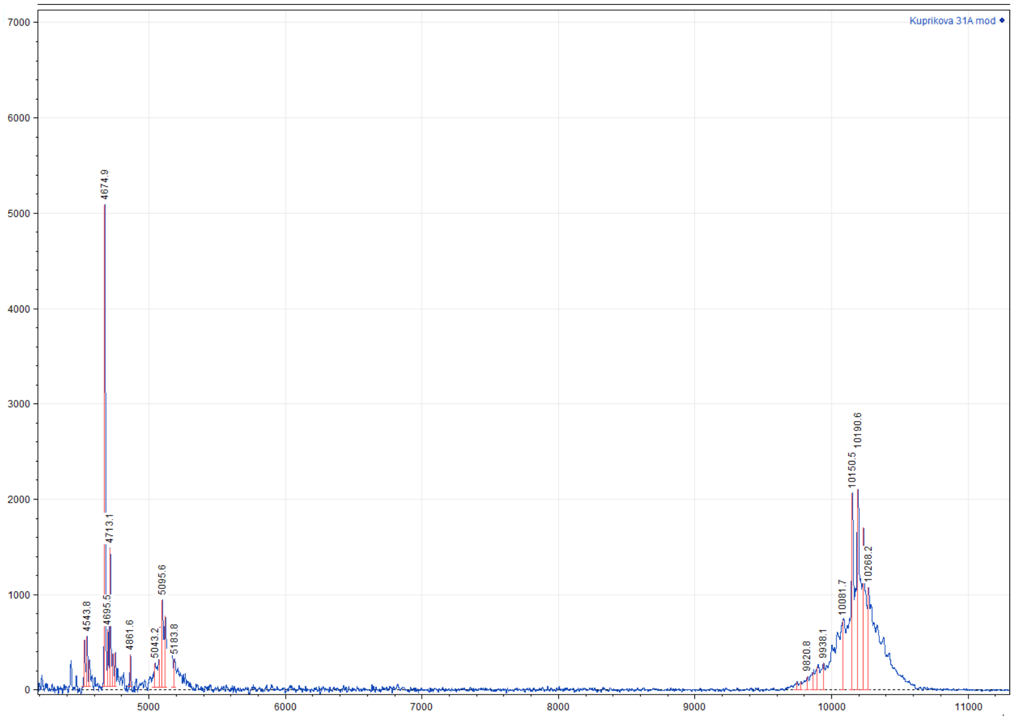

Figure S28. MALDI-TOF spectrum of **31ON_A**$^{\mathbf{OP}}$: calculated: 10149.3 Da; found: 10150.5 Da; Δ = 1.2 Da.

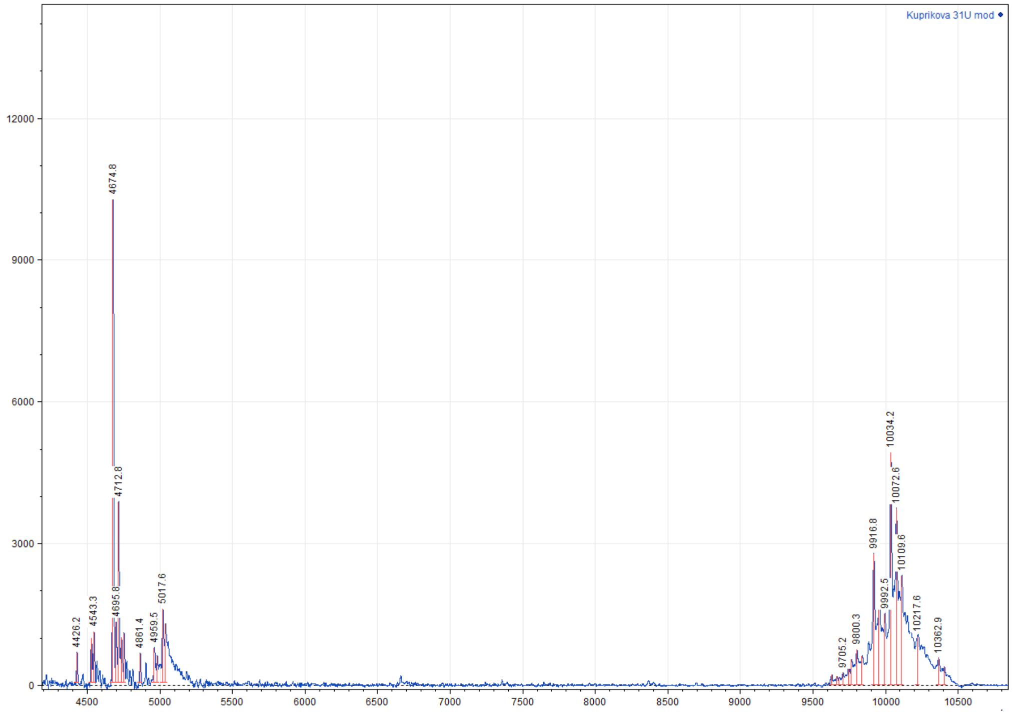

Figure S29. MALDI-TOF spectrum of **31ON_U**$^{\mathbf{SA}}$: calculated: 10033.3 Da; found: 10034.2 Da; Δ = 0.9 Da.

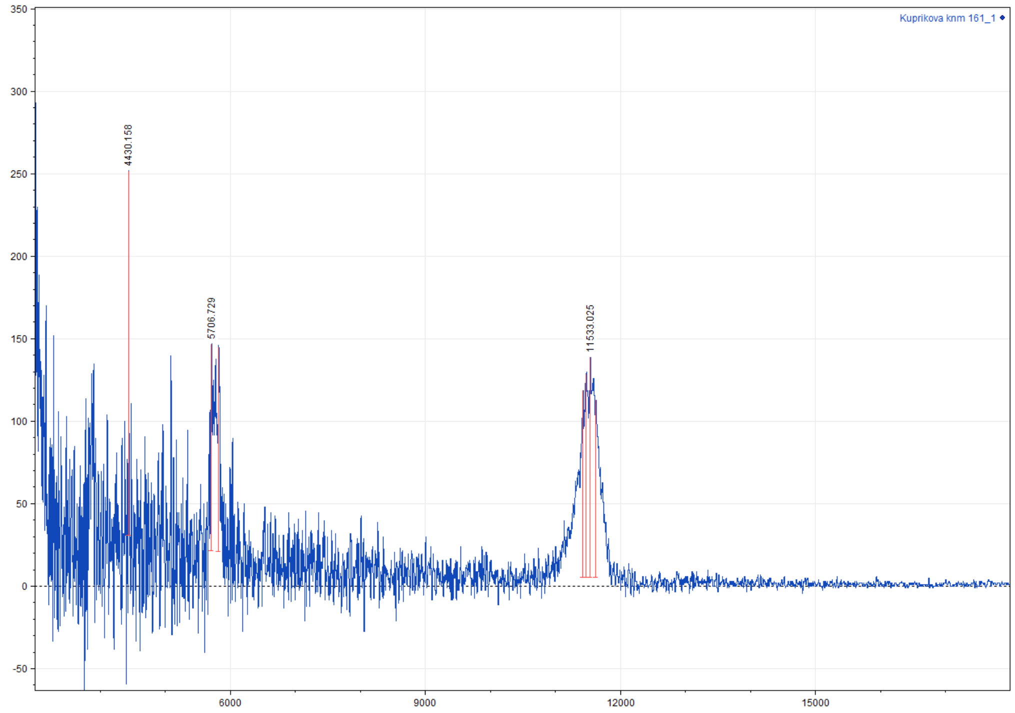

Figure S30. MALDI-TOF spectrum of **31ON_C**$^{\mathbf{CA}}$**G**$^{\mathbf{PA}}$**U**$^{\mathbf{SA}}$**A**$^{\mathbf{OP}}$: calculated: 11529.3 Da; found: 11533.0 Da; Δ = 3.7 Da.

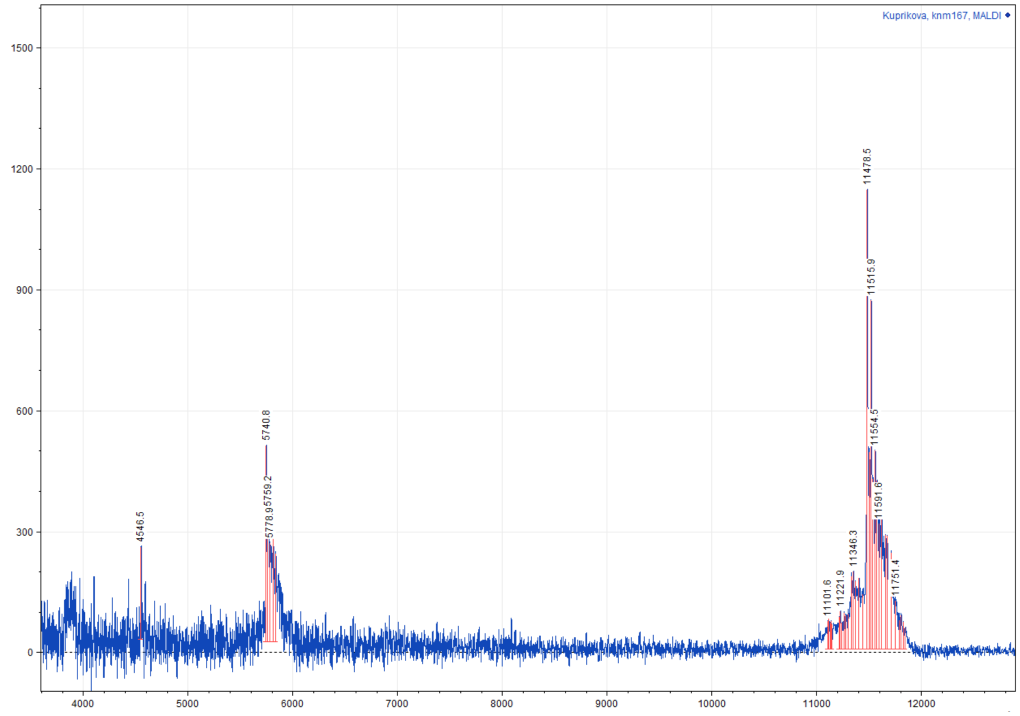

Figure S31. MALDI-TOF spectrum of **31ON_C**$^{\mathbf{CA}}$**G**$^{\mathbf{PA}}$**U**$^{\mathbf{EPh}}$**A**$^{\mathbf{EIn}}$: calculated: 11477.3 Da; found: 11478.5 Da; Δ = 1.2 Da.

7. Copies of NMR spectra

7.1. ^{1}H, ^{13}C and $^{31}P\{^{1}H\}$NMR spectra of dA^{OP}TP

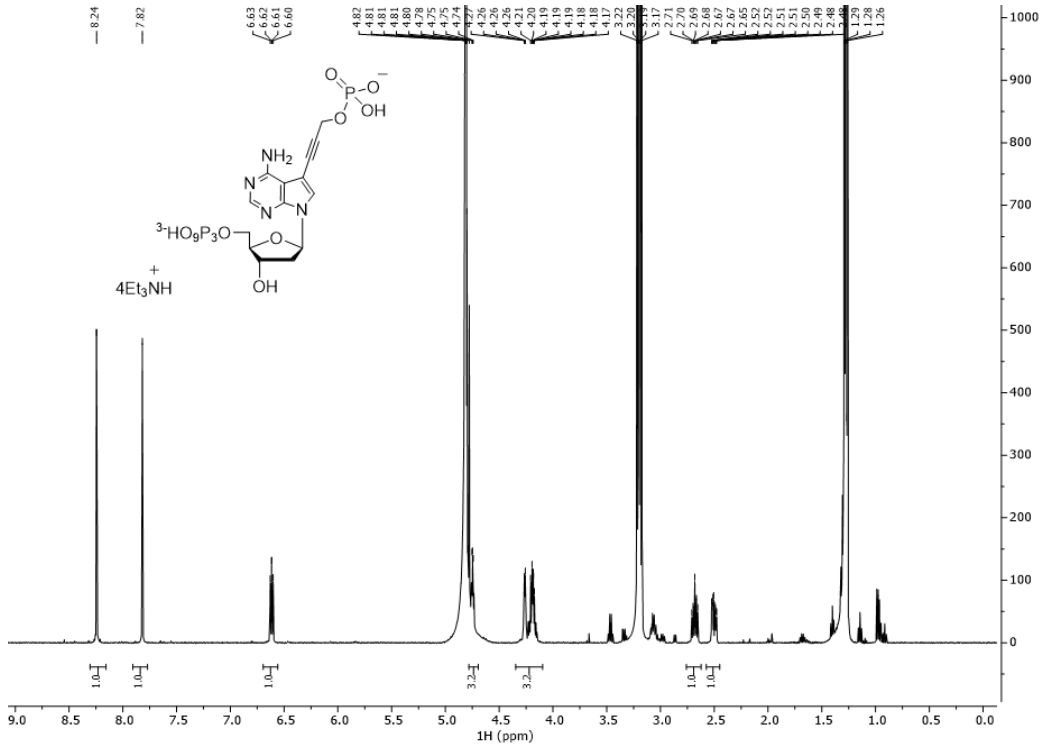

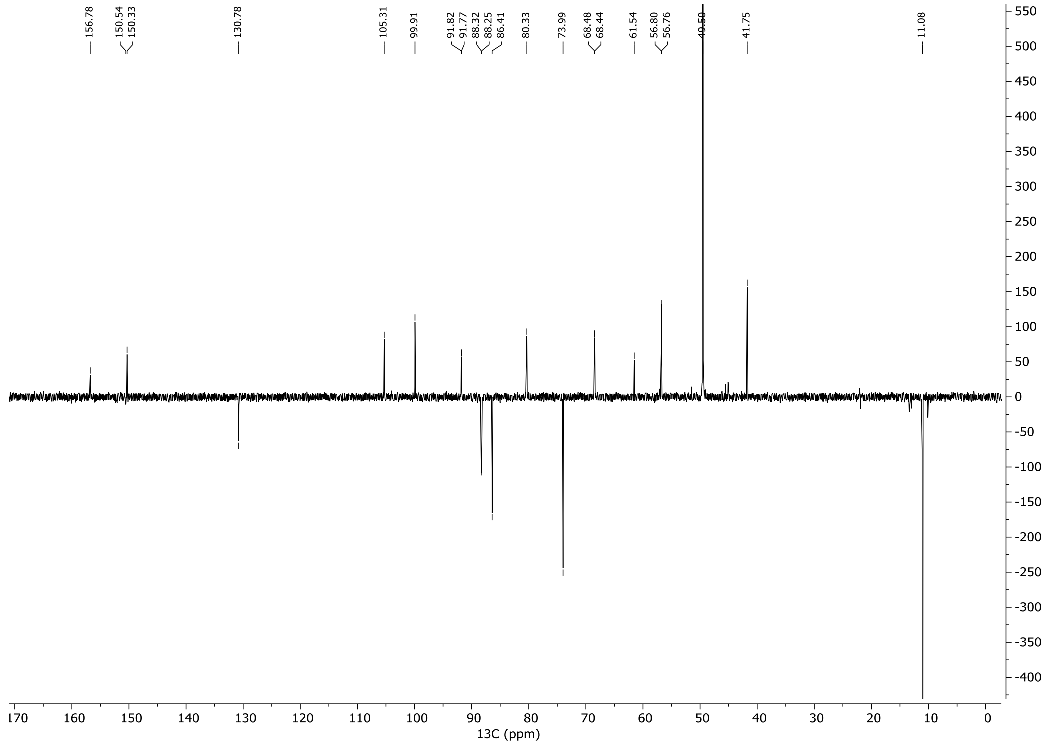

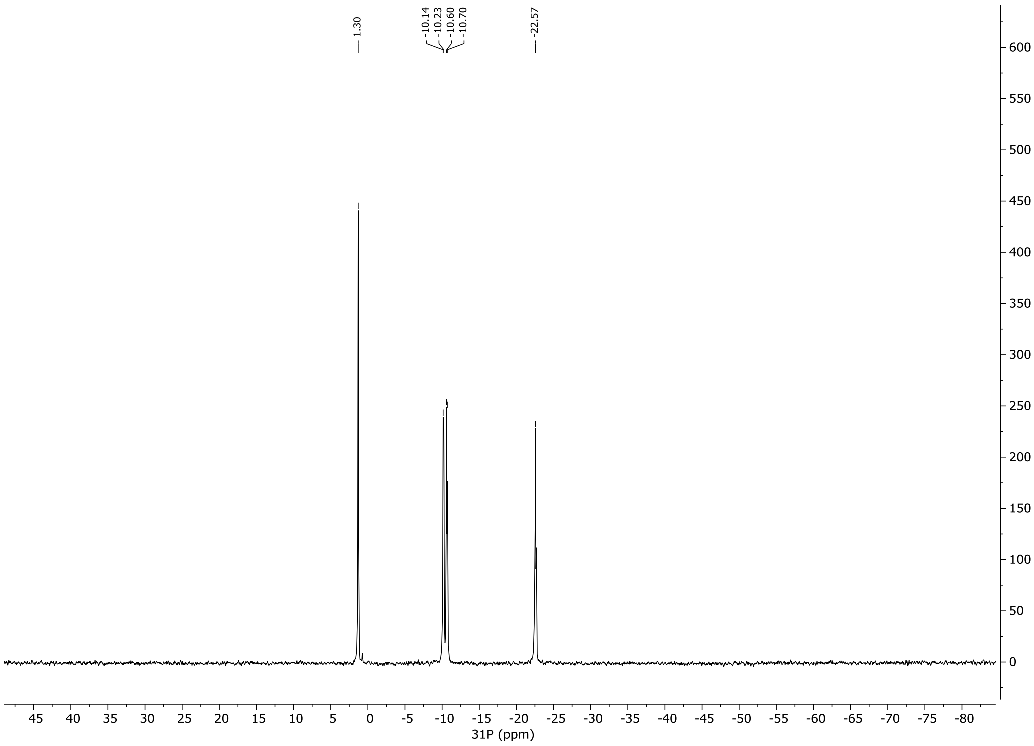

7.2. ^{1}H, ^{13}C and $^{31}P\{^{1}H\}$ NMR spectra of dG^{PA}TP

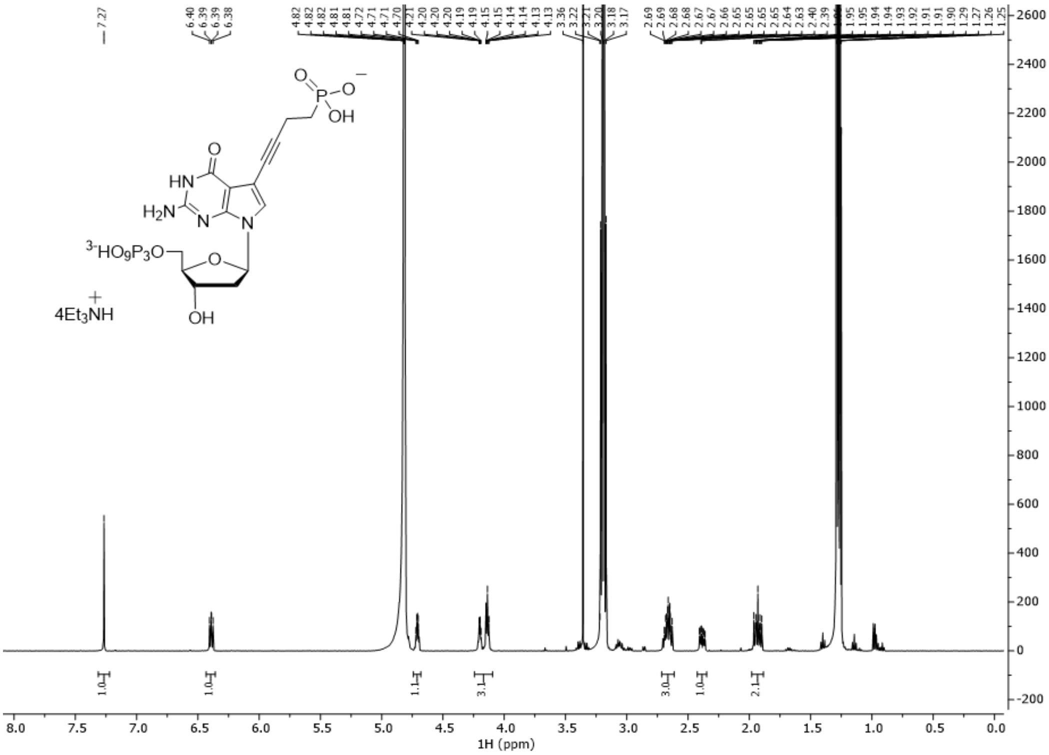

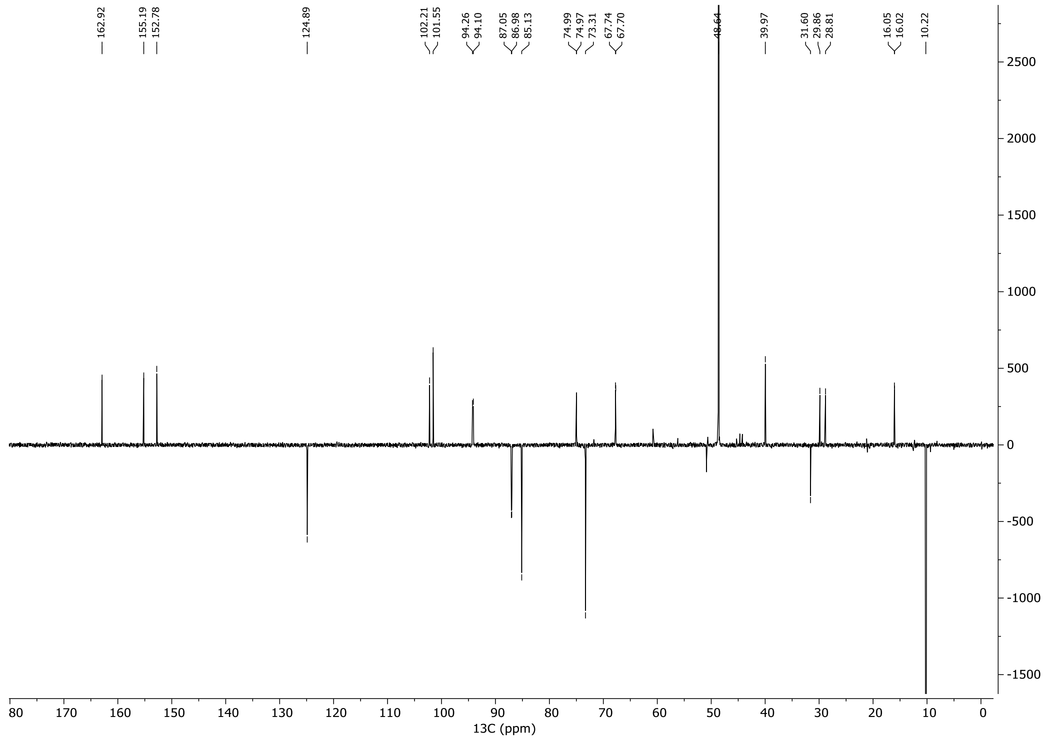

162.92
155.19
152.78
124.89
102.21
101.55
94.26
94.10
87.05
86.98
85.13
74.99
74.97
73.31
67.74
67.70
48.64
39.97
31.60
29.86
28.81
16.05
16.02
10.22
13C (ppm)

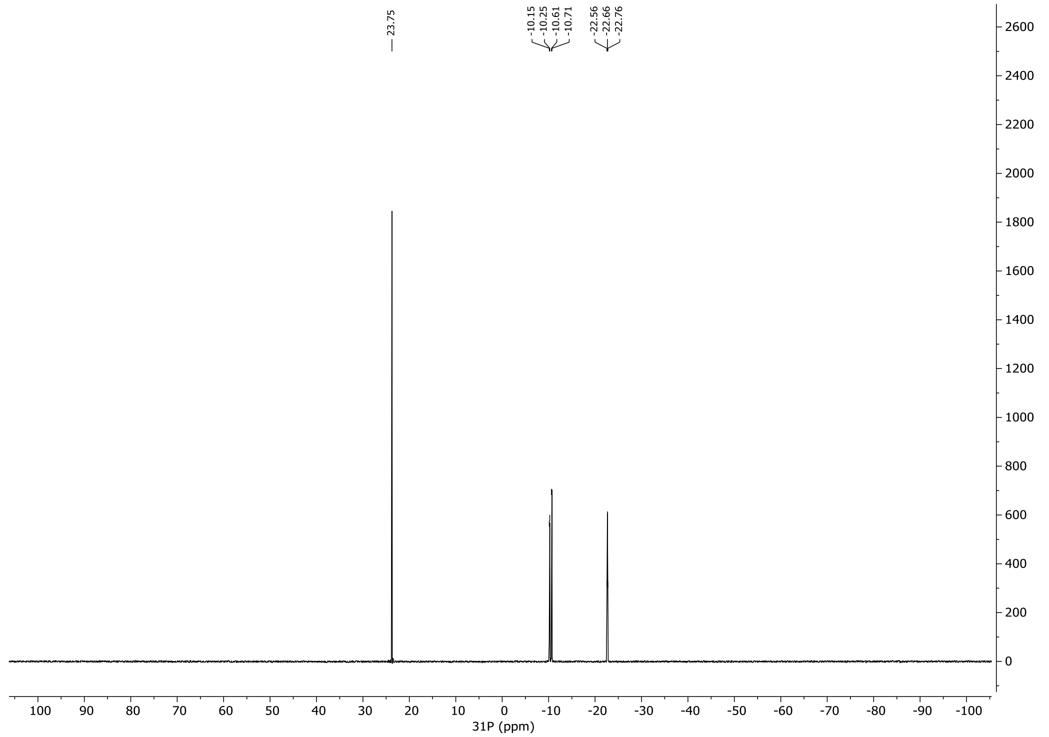

23.75
-10.15
-10.25
-10.61
-10.71
-22.56
-22.66
-22.76
31P (ppm)

7.3. ^{1}H, ^{13}C and $^{31}P\{^{1}H\}$ NMR spectra of dU^{SA}TP

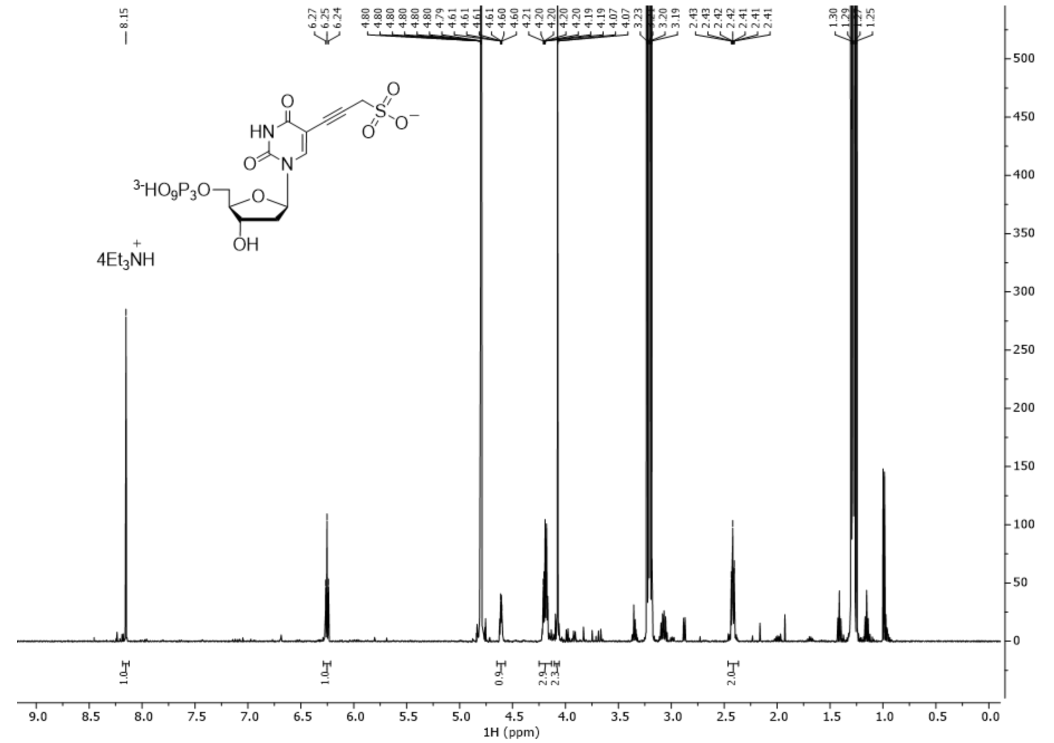

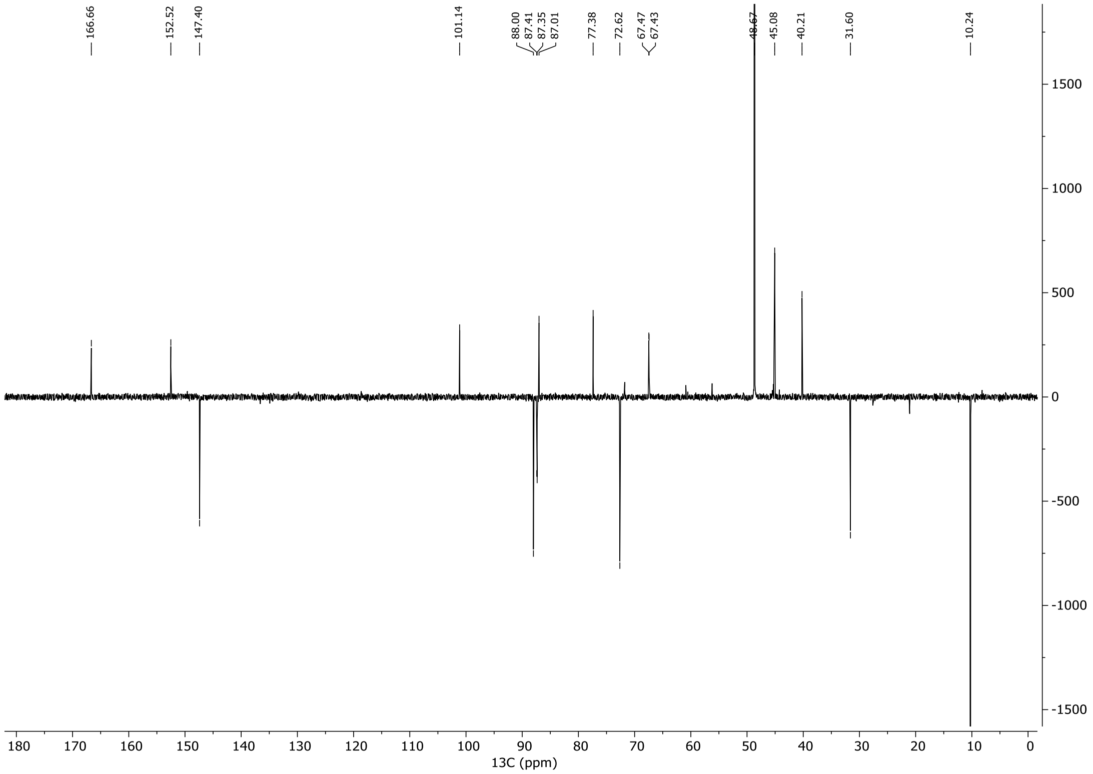

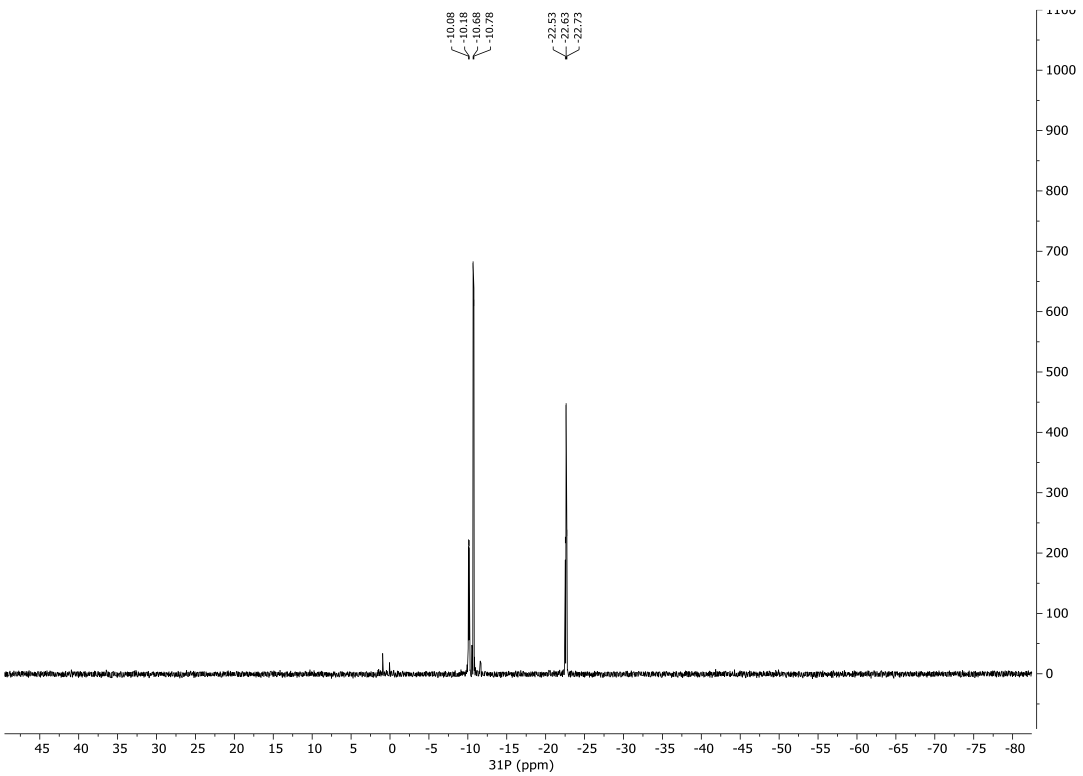

7.4. ^{1}H, ^{13}C and $^{31}P\{^{1}H\}$ NMR spectra of dC^{CA}TP

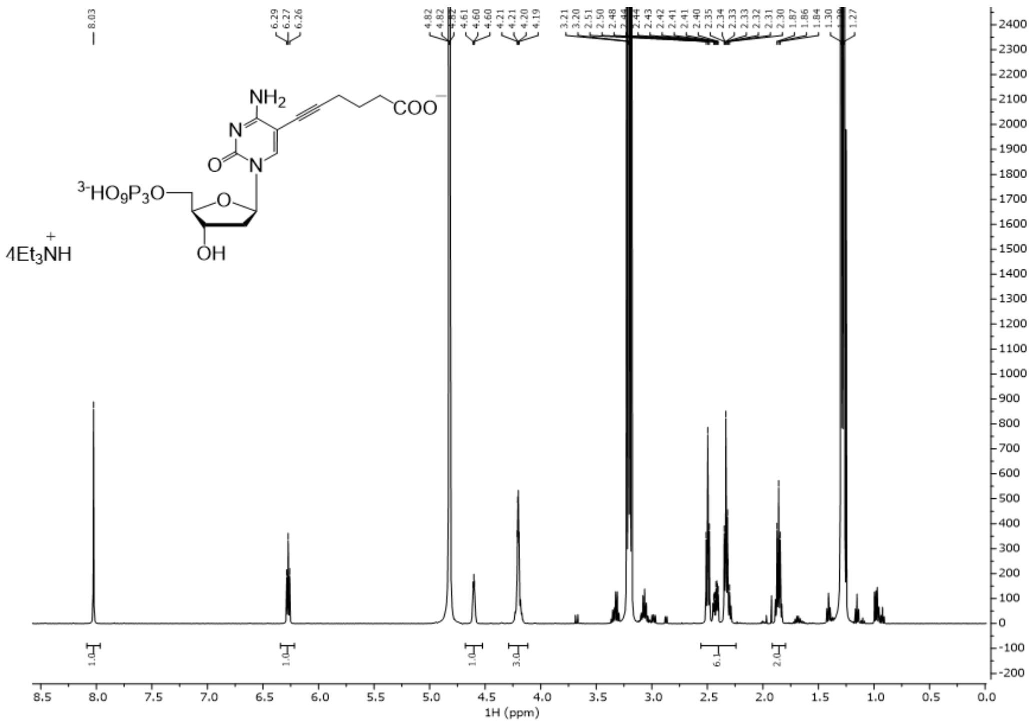

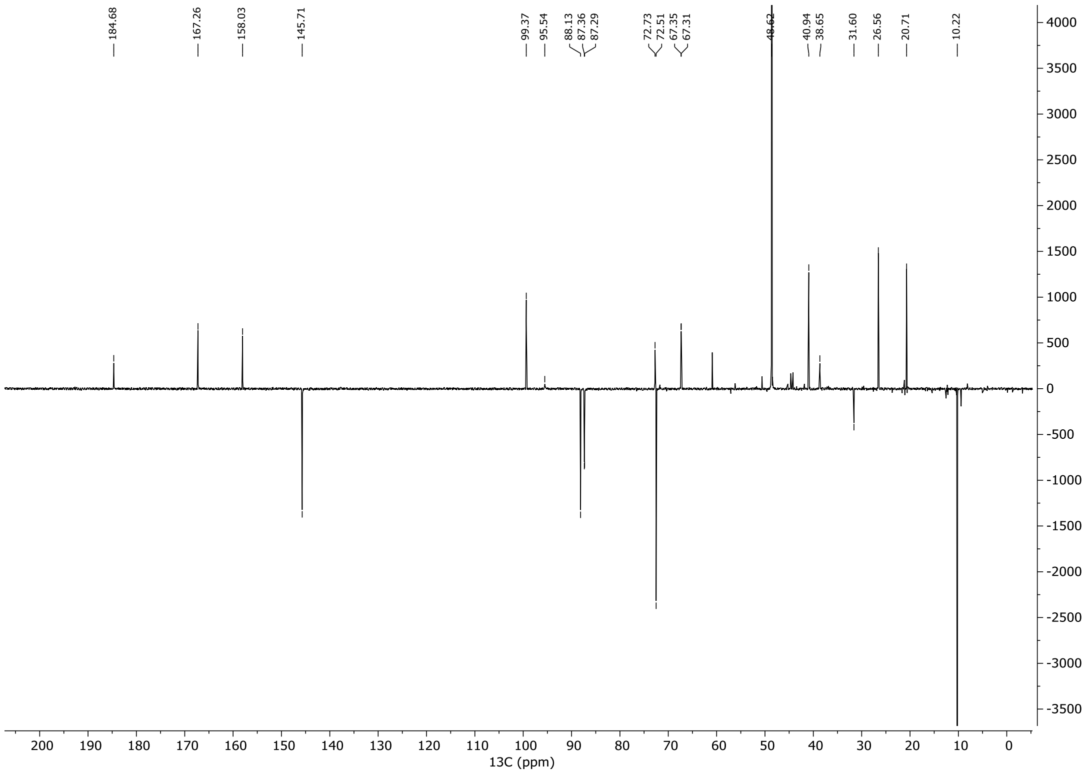
184.68
167.26
158.03
145.71
99.37
95.54
88.13
87.36
87.29
72.73
72.51
67.35
67.31
48.62
40.94
38.65
31.60
26.56
20.71
10.22
13C (ppm)

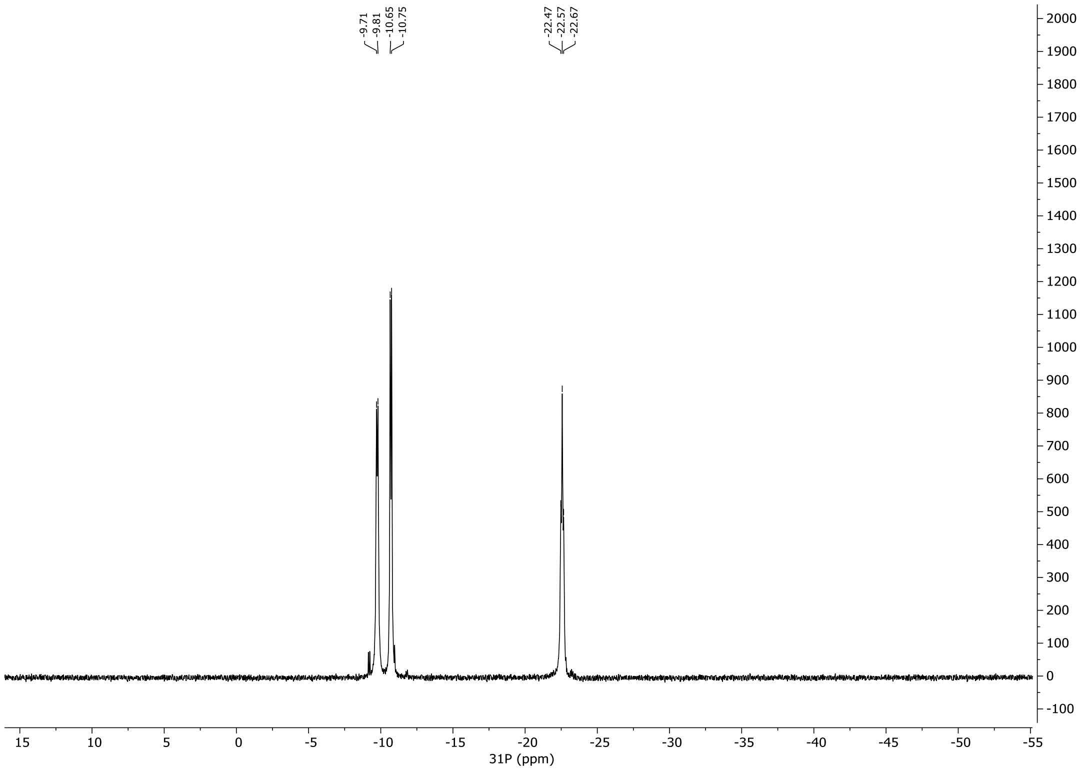
-9.71
-9.81
-10.65
-10.75
-22.47
-22.57
-22.67
31P (ppm)

8. References

1. Čapek,P., Cahová,H., Pohl,R., Hocek,M., Gloeckner,C., Marx,A. (2007) An Efficient Method for the Construction of Functionalized DNA Bearing Amino Acid Groups through Cross-Coupling Reactions of Nucleoside Triphosphates Followed by Primer Extension or PCR. *Chem. Eur. J.*, **13**, 6196-6203.
2. Cahová,H., Havran,L., Brázdilová,P., Pivoňková,H., Pohl,R., Fojta,M., Hocek,M. (2008) Aminophenyl- and Nitrophenyl-Labeled Nucleoside Triphosphates: Synthesis, Enzymatic Incorporation, and Electrochemical Detection. *Angew. Chem. Int. Ed.*, **47**, 1433-7851.
3. Le,B.H., Koo,J.Ch., Joo,H.N., Seo,Y.J. (2017) Diverse size approach to incorporate and extend highly fluorescent unnatural nucleotides into DNA. *Bioorg. Med. Chem.*, **25**, 3591-3596.
4. Sýkorová,V., Tichý,M., Hocek,M. (2022) Polymerase Synthesis of DNA Containing Iodinated Pyrimidine or 7-Deazapurine Nucleobases and Their Post-synthetic Modifications through the Suzuki-Miyaura Cross-Coupling Reactions. *ChemBioChem*, **23**, e202100608.
5. Wanat,P., Walczak,S., Wojtczak,B.A., Nowakowska,M., Jemielity,J., Kowalska,J. (2015) Ethynyl, 2-Propynyl, and 3-Butynyl C-Phosphonate Analogues of Nucleoside Di- and Triphosphates: Synthesis and Reactivity in CuAAC. *Org. Lett.*, **17**, 3062-3065.
6. Ouadahi,K., Allard,E., Oberleitner,B., Larpent,C. (2012) Synthesis of azide-functionalized nanoparticles by microemulsion polymerization and surface modification by click chemistry in aqueous medium. *Polym. Chem.*, **50**, 314–328.
7. Lee,L.V., Mitchell,M.L., Huang,S., Fokin,V.V., Sharpless,K.B., Wong,C. (2003) A Potent and Highly Selective Inhibitor of Human α-1,3-Fucosyltransferase via Click Chemistry. *J. Am. Chem. Soc.*, **125**, 9588-9589.
8. Ondruš,M., Sýkorová,V., Bednárová,L., Pohl,R., Hocek,M. (2020) Enzymatic synthesis of hypermodified DNA polymers for sequence-specific display of four different hydrophobic groups. *Nucleic Acids Res.*, **48**, 11982-11993.
9. Huang,J., Rauscher,S., Nawrocki,G., Ran,T., Feig,M., De Groot,B.L., Grubmüller,H., MacKerell,A.D. (2016) CHARMM36m: An improved force field for folded and intrinsically disordered proteins. *Nat. Methods.*, **14**, 71–73.
10. Lee,J., Cheng,X., Swails,J.M., Yeom,M.S., Eastman,P.K., Lemkul,J.A., Wei,S., Buckner,J., Jeong,J.C., Qi,Y., Jo,S., Pande,V.S., Case,D.A., Brooks,C.L., MacKerell,A.D., Klauda,J.B., Im,W. (2016) CHARMM-GUI Input Generator for NAMD, GROMACS, AMBER, OpenMM,

and CHARMM/OpenMM Simulations Using the CHARMM36 Additive Force Field. *J. Chem. Theory Comput*., **12**, 405–413 (2016).

11. Danne,R., Poojari,C., Martinez-Seara,H., Rissanen,S., Lolicato,F., Róg,T., Vattulainen,I. (2017) doGlycans–tools for preparing carbohydrate structures for atomistic simulations of glycoproteins, glycolipids, and carbohydrate polymers for gromacs. *J. Chem. Inf. Model*., **57**, 2401–2406.
12. Jorgensen,W.L., Chandrasekhar,J., Madura,J.D., Impey,R.W., Klein,M.L. (1983) Comparison of simple potential functions for simulating liquid water. *J. Chem. Phys*., **79**, 926–935.
13. Bussi,G., Donadio,D., Parrinello,M. (2007) Canonical sampling through velocity rescaling. *J. Chem. Phys*., **126**, 014101.
14. Bernetti,M., Bussi,G. (2020) Pressure control using stochastic cell rescaling. *J. Chem. Phys*., **153**, 114107.
15. Essmann,U., Perera,L., Berkowitz,M.L., Darden,T., Lee,H., Pedersen,L.G. (1995) A smooth particle mesh Ewald method. *J. Chem. Phys*., **103**, 8577–8593.
16. Steinbach,P.J., Brooks,B.R. (1994) New spherical-cutoff methods for long range forces in macromolecular simulation. *J. Comput. Chem*., **15**, 667–683.
17. Miyamoto,S., Kollman,P.A. (1992) Settle: An analytical version of the SHAKE and RATTLE algorithm for rigid water models. *J. Comput. Chem*., **13**, 952–962.
18. Hess,B., Bekker,H., Berendsen,H.J., Fraaije,J.G. (1997) LINCS: A Linear Constraint Solver for molecular simulations. *J. Comput. Chem*., **18**, 1463–1472.
19. Hess,B. (2008) P-LINCS: A parallel linear constraint solver for molecular simulation. *J. Chem. Theory Comput*., **4**, 116–122.
20. Abraham,M.J., Murtola,T., Schulz,R., Páll,S., Smith,J.C., Hess,B., Lindah,E. (2015) GROMACS: High performance molecular simulations through multilevel parallelism from laptops to supercomputers. *SoftwareX*, **1-2**, 19–25.
21. Michaud-Agrawal,N., Denning,E.J., Woolf, T.B., Beckstein,O. (2011) MDAnalysis: a toolkit for the analysis of molecular dynamics simulations. *J. Comput. Chem*., **32**, 2319–2327.
22. Gowers,R.J., Linke,M., Barnoud,J., Reddy,T.J.E., Melo,M.N., Seyler,S.L., Domański,J., Dotson,D.L., Buchoux,S., Kenney,I.M., Beckstein,O. (2016) MDAnalysis: A Python Package for the Rapid Analysis of Molecular Dynamics Simulations, in Proceedings of the 15th Python in Science Conference, 98–105.
